# Digital interventions to promote psychological resilience: a systematic review and meta-analysis

**DOI:** 10.1038/s41746-024-01017-8

**Published:** 2024-02-08

**Authors:** Sarah K. Schäfer, Lisa von Boros, Lea M. Schaubruch, Angela M. Kunzler, Saskia Lindner, Friederike Koehler, Tabea Werner, Federico Zappalà, Isabella Helmreich, Michèle Wessa, Klaus Lieb, Oliver Tüscher

**Affiliations:** 1 Leibniz Institute for Resilience Research, Mainz, Germany; 2 Department of Clinical Psychology, Psychotherapy and Diagnostics - Child and Adolescent Psychology and Psychotherapy, Technische Universität Braunschweig, Braunschweig, Germany; 3 Institute for Evidence in Medicine, Medical Center – University of Freiburg, Faculty of Medicine, University of Freiburg, Freiburg, Germany; 4 Department of Psychiatry and Psychotherapy, University Medical Center of Johannes Gutenberg University Mainz, Mainz, Germany; 5 Department of Clinical Psychology and Neuropsychology, Institute for Psychology, Johannes Gutenberg University Mainz, Mainz, Germany; 6 Centre of Excellence in Music, Mind, Body and Brain, University of Jyväskylä, Jyväskylä, Finland; 7 Institute for Molecular Biology, Johannes Gutenberg University Mainz, Mainz, Germany

**Keywords:** Lifestyle modification, Public health

## Abstract

Societies are exposed to major challenges at an increasing pace. This underscores the need for preventive measures such as resilience promotion that should be available in time and without access barriers. Our systematic review summarizes evidence on digital resilience interventions, which have the potential to meet these demands. We searched five databases for randomized-controlled trials in non-clinical adult populations. Primary outcomes were mental distress, positive mental health, and resilience factors. Multilevel meta-analyses were performed to compare intervention and control groups at post-intervention and follow-up assessments. We identified 101 studies comprising 20,010 participants. Meta-analyses showed small favorable effects on mental distress, SMD = –0.24, 95% CI [–0.31, –0.18], positive mental health, SMD = 0.27, 95% CI [0.13, 0.40], and resilience factors, SMD = 0.31, 95% CI [0.21, 0.41]. Among middle-aged samples, older age was associated with more beneficial effects at follow-up, and effects were smaller for active control groups. Effects were comparable to those of face-to-face interventions and underline the potential of digital resilience interventions to prepare for future challenges.

## Introduction

An increasing number of environmental and socioeconomic challenges and major disruptive events worldwide poses a significant threat to public mental health^1^. Recently, evidence for adverse mental health effects of stressors like the COVID-19 pandemic or armed conflicts has resulted in an increased interest in (psychological) resilience^2–4^. Resilience as an outcome describes the maintenance of stable good mental health or the quick recovery of mental health during or after stressor exposure^5^. However, rather than being a categorical outcome, resilience varies between different domains of life and fluctuates over time^6,7^. Promoting resilience at a population level may help societies to be better prepared for future disruptions^8^.

Resilience-promoting interventions describe a heterogeneous category of interventions aiming to promote resilience as an outcome mostly by fostering so-called resilience factors and, less common though, higher-level, neurocognitive resilience mechanisms^9^. Resilience factors are internal and external resources that come into play when coping with various stressors^1^. These factors include dispositional variables such as resilience-promoting traits (e.g., optimism), beliefs (e.g., self-efficacy), and coping strategies (e.g., flexible or active coping)^9,10^, social and cultural factors (e.g., perceived social support, community cohesion)^6^. Recent approaches in resilience research suggest that these resilience factors show substantial interrelations^10^ and may converge into a smaller number of higher-level resilience mechanisms (e.g., positive appraisal style^11^; regulatory flexibility^12^), which mediate their association with resilient outcomes^11^. Most resilience-promoting interventions use approaches adapted from psychotherapy (e.g., cognitive-behavioral or mindfulness-based interventions) to enhance these factors with a broad set of exercises^13,14^ (e.g., psychoeducation, relaxation, training of cognitive strategies).

Previous research provided evidence for small to moderate favorable effects of resilience-promoting interventions in high-risk groups (e.g., healthcare workers, police staff^13,15,16^), non-clinical (e.g., students^17,18^) and clinical populations (e.g., diabetes or cancer patients^19,20^). However, many of those interventions were delivered in face-to-face settings using individual or group trainings. Those interventions may have favorable effects, but at the same time they can only be delivered to a small number of people at a time, require substantial staff and financial resources and cannot be easily tailored to participants’ needs, individual time constraints^21^, and demands in low-resource settings^22^. Moreover, especially the COVID-19 pandemic highlighted that stressor exposure itself may stop the availability of in-person interventions resulting in situations where resilience promotion would be of major importance but cannot be delivered^23^.

Digital resilience interventions may help to address these shortcomings as they can be delivered to a large number of people at the same time, require a lot less staff and (in the long run) fewer financial resources^21^, making them promising for low-resource settings^24^. Digital resilience interventions may also be tailored to participants’ needs and allow for flexible time plans (e.g., shift schedules)^25^. Moreover, digital resilience interventions may still be available when stressors like the pandemic prevent in-person meetings. Thus, developing effective digital resilience interventions might be a key component of preparedness for future pandemics and other types of disruptions and challenges.

Previous systematic reviews combined digital and in-person resilience-promoting interventions^13,15,26^ or examined a very small number of studies with highly restrictive inclusion criteria^21,27^ (e.g., only studies that employed stand-alone online interventions). Moreover, those reviews primarily examined the effects of digital resilience interventions on self-reported resilience^21,27^. However, most of these measures fail to meet state-of-the-art resilience conceptualizations, which define resilient outcomes rather as a trajectory of stable good mental health in face of stress than a dispositional variable^5^. Thus, following these state-of-the-art approaches, effects on mental distress and positive mental health are even more important than changes in self-reported resilience^9^.

The present systematic review and meta-analysis aim at addressing these gaps by applying a broader and more comprehensive definition of resilience-promoting interventions, as used in two recent Cochrane reviews^13,15,28^, also including interventions that build on the resilience concept or aim at enhancing hardiness or growth from stress exposure as related concepts. Moreover, we also examine interventions with blended designs that combine in-person interventions with digital components. In line with recent conceptualizations of resilience^1,5,9^, we examine mental distress and positive mental health as primary outcomes and study resilience factors as secondary outcomes. With this focus on resilience factors, we provide a proof of concept that has not been done in previous reviews on digital resilience interventions^21,27^. Based on a large number of studies, we also examine a broad range of potential moderators including sociodemographic sample characteristics (i.e., age, gender, population type), intervention characteristics (i.e., delivery format, theoretical foundation, availability of guidance, degree of individualization, intervention intensity, availability of in-person components), and aspects of study design (i.e., type of control group). We compare our findings with previous reviews on in-person resilience interventions and derive recommendations for the use of digital resilience interventions to prepare for future major disruptions.

## Results

### Search outcomes

**Fig. 1:**
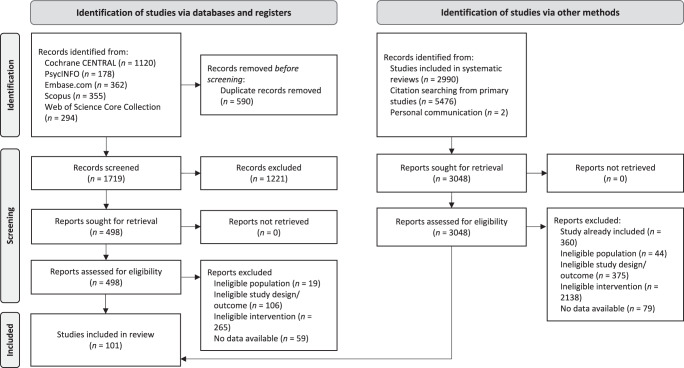
PRISMA flowchart. *Note*. Flowchart according to the Preferred Reporting Items for Systematic Reviews and Meta-Analyses^77^. *n* = number of studies/records/reports.

Our search for primary studies in electronic databases yielded 2309 eligible records, with 590 duplicates being removed. Of 1719 records screened at title/abstract level, 498 were assessed at full text level, of which 49 were identified as eligible. Another 52 eligible records were identified by our search in systematic reviews, references cited in eligible primary studies and personal communication. Taken together, this resulted in 101 eligible primary studies for the quantitative synthesis (see Fig. 1).

### Study characteristics

Supplementary Data 3 presented the characteristic of 101 included studies (comprising 20,010 participants) published between 2007 and 2023, with the vast majority being published from 2015 onwards (83.2%). Most studies were performed in the United States (31 studies, 30.7%), Germany (11 studies, 10.9%), and the United Kingdom (7 studies, 6.9%; see Supplementary Data 3 for all countries). The vast majority of studies was conducted in high-income countries, with only 11 studies (10.9%) being carried out in middle- or low-income countries that are more likely to represent low-resource settings.

On average participants were 34.4 years old (*SD* 9.78; range: 17.9–57.6 years) and 71.5% of the participants were female (range: 0–100%). Thirty-six samples (35.6%) were recruited at their workplaces, 24 studies (23.8%) used student or university samples, 23 studies (22.8%) examined populations with increased stressor exposure in private life (e.g., informal caregivers), seven studies (6.9%) recruited samples from non-clinical military populations (e.g., military members and their partners), and 11 studies (10.9%) reported on not further specified non-clinical populations.

Sixty-five studies (64.3%) reported on online interventions, 21 studies (20.8%) employed mobile-based interventions, seven studies (6.9%) used mixed interventions comprising both web- and app-based components, and four studies (4.0%) reported on interventions using (at least partly) a lab-based delivery via computers. Only four studies (4.0%) used blended delivery formats combining digital and in-person components. Twenty-three studies (22.8%) combined different theoretical concepts for their interventions (e.g., CBT and mindfulness), 22 studies (21.8%) employed solely CBT-based interventions, followed by 17 studies (16.8%) reporting on mindfulness-based interventions, and 11 studies (10.9%) employing interventions that were based on positive psychology. The remaining 28 studies (27.7%) used other theoretical approaches (see Supplementary Data 3), with only three studies (3.0%) employing interventions grounded in specific resilience theories. Intervention duration ranged between 1-session interventions and delivery over 52 weeks (average duration: 6.2 weeks [*SD* 6.7]). Most interventions were unguided (69 studies; 68.3%), while 32 interventions (31.7%) were at least partly guided by trained lays or professionals.

Fifty-eight studies (57.4%) used passive comparators (i.e., waitlist, no intervention), while 16 studies (15.8%) employed low-intensity active comparators (i.e., some kind of control intervention, but of shorter duration and less attention) and 27 studies used high-intensity active comparators (26.7%; i.e., interventions with similar duration and attention).

Of the 43 studies reporting follow-up data, 29 studies (67.4%) reported on follow-up assessments 1-to-3 months after the end of the intervention, while 8 studies (18.6%) performed follow-ups between 3 and 6 months, and 6 studies (14.0%) between 6 and 12 months.

### Risk of bias

**Fig. 2:**
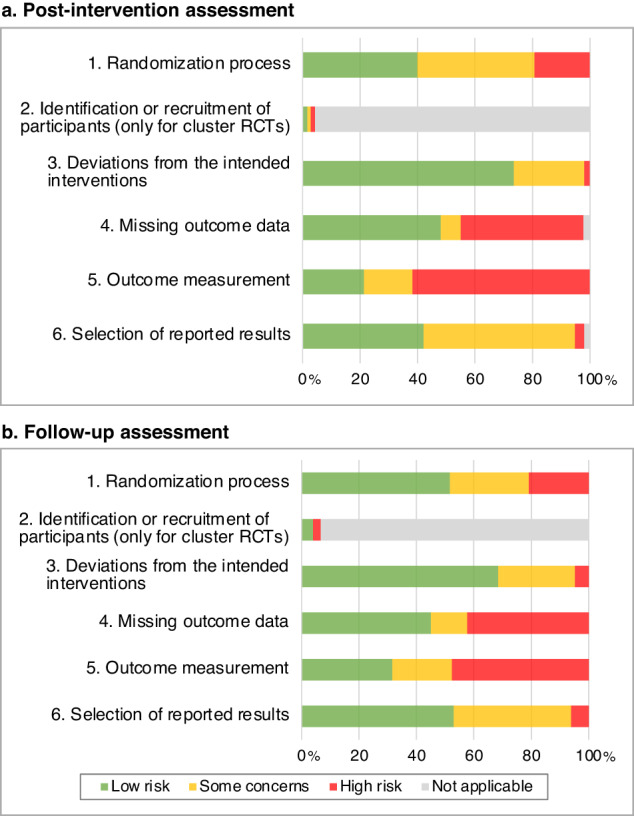
Risk of bias across effect estimates at post-intervention and follow-up assessments. *Note*. Risk of bias in percentages across effect estimates assessed using the Cochrane risk-of-bias tool for randomized trials (RoB2)^30^. Percentages at post-intervention assessment relate to 337 effect estimates from 101 studies included in our review (**a**), while percentages at follow-up assessment relate to 146 effect estimates from 43 studies (**b**).

There was a moderate to high risk of bias (see Fig. 2 and Supplementary Data 4). Main flaws (≥20% of some concerns or high risk) across included effect estimates were found for measurement of outcome (post-intervention: 79% some concerns or high risk; follow-up: 55%), randomization (post-intervention: 60%; follow-up: 48%), selection of reported results (post-intervention: 56%; follow-up: 47%), missing outcome data (post-intervention: 50%; follow-up: 55%), and deviations from intended interventions (post-intervention: 27%; follow-up: 32%). The number of cRCTs was low, thus, the impact of bias from the recruitment of participants was low (post-intervention/follow-up: 3%).

### Publication bias

Neither contour-enhanced funnel plots (see Supplementary Data 5) nor meta-regression models provided evidence for a publication bias at post-intervention assessments for mental distress, *QM*(1) = 0.77, *p* = 0.384, and positive mental health, *QM*(1) = 1.08, *p* = 0.302, while the meta-regression model on resilience factors suggested funnel plot asymmetry, *QM*(1) = 4.52, *p* = 0.039. Also, contour-enhanced funnel plots showed that some effect estimates fell into the significance border areas. At follow-up assessments, we found no evidence for a publication bias neither using a regression-based approach [mental distress: *QM*(1) = 0.05, *p* = 0.821; positive mental health: *QM*(1) = 0.49 *p* = 0.488; resilience factors: *QM*(1) = 3.61, *p* = 0.076], nor based on the inspection of contour-enhanced funnel plots (see Supplementary Data 6).

### Effects of post-intervention assessment

Eighty-five studies (reporting 150 effect estimates) contributed to the meta-analysis on *mental distress* (see Supplementary Data 7 for forest plot). Across five outcome types, we found evidence for a small favorable effect of digital resilience interventions, SMD = –0.24, 95% CI [–0.31, –0.18], with substantial heterogeneity, *Q*(149) = 366.22, *p* <0.001 (see Table 1). Heterogeneity was partly accounted for by between-outcome differences, *QM*(4) = 5.12, *p* = 0.001. Small favorable effects were found consistently across all outcome types ranging from SMD = –0.14, 95% CI [–0.25, –0.04], for general distress to SMD = –0.33, 95% CI [–0.41, –0.24], for stress symptoms, with moderate to substantial heterogeneity across outcome types (41.4 ≤ *I*^*2*^ ≥ 66.8). Heterogeneity remained significant after accounting for between-outcome differences, *Q*(145) = 323.71, *p* <0.001.

**Table 1:** Results of main analyses for primary outcomes comparing digital resilience interventions and comparators.

Analysis	*n*	*k*	SMDs	95% CI	95% PI	*p*	*Q*	*df*	*p(Q)*	*I*^*2*^
***(A) Post-intervention assessment***										
***Mental distress***	85	150	–0.24	[–0.31, –0.18]	[–0.71, 0.22]	<0.001	366.22	149	<0.001	
Anxiety symptoms	34	34	–0.19	[–0.27, –0.11]	[–0.63, 0.25]	<0.001				62.8
Depressive symptoms	48	48	–0.25	[–0.31, –0.19]	[–0.69, 0.19]	<0.001				66.8
General distress	13	13	–0.14	[–0.25, –0.04]	[–0.59, 0.30]	0.009				41.4
PTSD symptoms	15	15	–0.19	[–0.29, –0.09]	[–0.64, 0.25]	<0.001				42.2
Stress symptoms	40	40	–0.33	[–0.41, –0.24]	[–0.77, 0.12]	<0.001				60.9
***Positive mental health***	77	123	0.27	[0.13, 0.40]	[–0.85, 1.38]	<0.001	850.59	122	<0.001	
Happiness	6	6	0.07	[–0.17, 0.30]	–	0.586				85.4
Life satisfaction	9	9	0.26	[0.10, 0.41]	–	0.002				82.2
Mental health	5	5	0.56	[0.36, 0.76]	–	<0.001				77.7
Positive emotions/affect	14	14	0.27	[0.10, 0.45]	[–0.87, 1.42]	0.003				84.3
Stress-related/posttraumatic growth	8	8	0.31	[0.06, 0.56]	–	0.017				76.0
Quality of life	9	9	0.28	[–0.03, 0.59]	[–0.89, 1.46]	0.073				80.6
Resilience	46	46	0.22	[0.05, 0.39]	[–0.92, 1.37]	0.013				90.5
Vitality	3	3	0.39	[0.17, 0.61]	–	<0.001				66.6
Wellbeing	23	23	0.34	[0.17, 0.51]	[–0.81, 1.48]	<0.001				87.9
***Resilience factors***	45	64	0.31	[0.21, 0.41]	[–0.22, 0.84]	<0.001	229.66	63	<0.001	
Active coping	8	8	0.43	[0.08, 0.77]	–	0.017				58.1
Mindfulness	15	15	0.27	[0.13, 0.41]	[–0.28, 0.82]	<0.001				68.2
Optimism	7	7	0.39	[0.17, 0.62]	–	0.001				71.6
Self-compassion	9	9	0.47	[0.21, 0.73]	[–0.12, 1.06]	<0.001				67.2
Self-efficacy	12	12	0.18	[–0.03, 0.40]	[–0.39, 0.76]	0.090				74.5
Self-esteem	3	3	0.24	[0.04, 0.43]	–	0.019				73.3
Social support	10	10	0.26	[0.09, 0.44]	[–0.30, 0.82]	0.005				67.7
***B) Follow-up assessments***										
***Mental distress***	38	64	–0.24	[–0.35, –0.13]	[–0.82, 0.35]	<0.001	205.32	63	<0.001	
Anxiety symptoms	14	14	–0.21	[–0.34, –0.08]	[–0.80, 0.38]	0.002				72.3
Depressive symptoms	25	25	–0.23	[–0.35, –0.07]	[–0.81, 0.35]	<0.001				78.4
PTSD symptoms	10	10	–0.21	[–0.35, –0.07]	[–0.80, 0.38]	0.004				61.0
Stress symptoms	15	15	–0.29	[–0.46, –0.12]	[–0.89, 0.31]	0.001				69.3
***Positive mental health***	36	57	0.19	[0.11, 0.26]	[–0.20, 0.57]	<0.001	139.66	56	<0.001	
Happiness	3	3	0.10	[–0.04, 0.24]	–	0.169				80.7
Life satisfaction	5	5	0.15	[0.05, 0.25]	–	0.006				77.9
Mental health	3	3	0.31	[–0.08, 0.70]	–	0.118				68.9
Positive emotions/affect	5	5	0.25	[0.05, 0.46]	–	0.018				72.0
Stress-related/posttraumatic growth	5	5	–0.06	[–0.37, 0.26]	–	0.719				56.7
Quality of life	5	5	0.02	[–0.19, 0.23]	–	0.854				66.3
Resilience	20	20	0.23	[0.10, 0.36]	[–0.17, 0.62]	0.001				63.3
Wellbeing	11	11	0.27	[0.08, 0.46]	[–0.15, 0.69]	0.008				68.7
***Resilience factors***	18	25	0.19	[0.08, 0.30]	[–0.25, 0.62]	0.002	83.62	24	<0.001	
Mindfulness	4	4	0.10	[–0.13, 0.34]	–	0.350				43.2
Optimism	3	3	0.30	[–0.04, 0.64]	–	0.079				50.4
Self-compassion	6	6	0.33	[–0.02, 0.68]	–	0.063				42.7
Self-efficacy	4	4	0.01	[–0.39, 0.41]	–	0.938				42.4
Self-esteem	3	3	0.23	[0.10, 0.35]	–	0.002				40.3
Social support	5	5	0.15	[0.04, 0.25]	–	0.010				41.5

*Note*. The multilevel meta-analysis on distress indicators included anxiety symptoms, depressive symptoms, general distress, PTSD symptoms, and stress symptoms. Due to qualitative differences, positive mental health and resilience factors were analyzed separately. Positive mental health comprised measures of happiness, life satisfaction, mental health, positive emotions/affect, stress-related/posttraumatic growth, quality of life, resilience, vitality, and wellbeing. Resilience factors comprised active coping, mindfulness, optimism, self-compassion, self-efficacy, self-esteem, and social support. For distress indicators, negative SMDs indicate favorable effects of the intervention [i.e., lower distress in the digital resilience intervention group compared to the control group]. For positive mental health and resilience factors, positive SMDs indicate favorable intervention effects [i.e., higher levels of positive mental health and resilience factors in the digital resilience intervention group compared to the control group]. All tests and reported statistics use cluster-robust estimates to account for non-independent effect estimates within studies. df = degrees of freedom; *I*^*2*^ = heterogeneity index in percentage (range: 0–100%).

*k* number of effect estimates, *n* number of studies, *PTSD* posttraumatic stress disorder, *Q* Cochran’s Q statistic with *p* value, *SMD* standardized mean difference, *95% CI* 95% confidence interval, *95% PI* 95% prediction interval.

Seventy-seven studies (reporting 123 effect estimates) contributed to the meta-analysis on *positive mental health outcomes* (see Supplementary Data 8 for forest plot). Across nine outcome types, there was evidence for a small favorable effect of digital resilience interventions over comparators, SMD = 0.27, 95% CI [0.13, 0.40], *p* < 0.001, with substantial heterogeneity, *Q*(122) = 850.59, *p* <0.001. Heterogeneity was partly accounted for by between-outcome differences, *QM*(8) = 4.37, *p* < 0.001. Except for happiness, SMD = 0.07, 95% CI [–0.17, 0.30], and quality of life, SMD = 0.28, 95% CI [–0.03, 0.59], there was evidence for favorable effects for all outcome types ranging from small effects for self-reported resilience, SMD = 0.22, 95% CI [0.05, 0.39], to small-to-moderate effects for overall mental health, SMD = 0.56, 95% CI [0.36, 0.76], with substantial heterogeneity for all outcome types (66.6 ≤ *I*^*2*^ ≥ 90.5), Q(114) = 766.34, *p* < 0.001.

Forty-five studies reported 64 effect estimates for *resilience factors* (see Supplementary Data 9 for forest plot). Across seven resilience factors, there was evidence for small favorable effects, SMD = 0.31, 95% CI [0.21, 0.41], with substantial heterogeneity, *Q*(63) = 229.66, *p* <0.001, which mostly derived from between-study differences, while between-outcome differences accounted for a close-to-significant small proportion of heterogeneity, *QM*(6) = 2.13, *p* = 0.066. Effect estimates were non-significant for self-efficacy, SMD = 0.18, 95% CI [–0.03, 0.40]. For the remaining resilience factors, estimates ranged between small favorable effects for self-esteem, SMD = 0.24, 95% CI [0.04, 0.43], and small to moderate effects for self-compassion, SMD = 0.47, 95% CI [0.21, 0.73]. For single outcome types, heterogeneity was substantial (58.1 ≤ *I*^*2*^ ≥ 74.5), *Q*(57) = 203.95, *p* < 0.001.

According to GRADE ratings^29^, certainty of evidence was very low for all outcome categories (see Supplementary Data 10).

### Effects of follow-up assessment

Analyses on *mental distress* at follow-up assessments were based on 38 studies (comprising 64 effect estimates; see Supplementary Data 11 for forest plot) and yielded again evidence for a small favorable effect of digital resilience interventions over comparators, SMD = –0.24, 95% CI [–0.35, –0.13], with substantial heterogeneity, *Q*(63) = 205.32, *p* < 0.001, mainly resulting from between-study differences, while between-outcome differences were of minor relevance, *QM*(3) = 0.63, *p* = 0.602. Across all outcomes, favorable effects were small ranging from SMD = –0.21, 95% CI [–0.34, –0.08], for anxiety symptoms to SMD = –0.29, 95% CI [–0.46, –0.12], for stress symptoms. Residual heterogeneity was significant, *Q*(60) = 195.00, *p* < 0.001, and substantial across all outcome types (61.0 ≤ *I*^*2*^ ≥ 78.4).

At follow-up assessments, based on 57 effect estimates from 36 studies (see Supplementary Data 12 for forest plot), there was evidence for small favorable effects of digital resilience interventions on *positive mental health*, SMD = 0.19, 95% CI [0.11, 0.26], with substantial heterogeneity, *Q*(56) = 139.66, *p* < 0.001, mainly resulting from between-study differences, while between-outcome differences were of minor relevance, *QM*(7) = 1.68, *p* = 0.352. At single outcome level, no effects emerged for happiness, mental health, stress-related/posttraumatic growth, and quality of life, while small favorable effects were found for other outcomes ranging from SMD = 0.15, 95% CI [0.05, 0.25], for life satisfaction to SMD = 0.27, 95% CI [0.08, 0.46], for wellbeing. Heterogeneity remained significant, *Q*(49) = 117.65, *p* < 0.001, and substantial across all outcome types (56.7 ≤ *I*^*2*^ ≥ 80.7).

Eighteen studies (reporting 25 effect estimates) assessed *resilience factors* at follow-up assessments (see Supplementary Data 13 for forest plot), finding an overall small favorable effect, SMD = 0.19, 95% CI [0.08, 0.30], with substantial heterogeneity, *Q*(24) = 83.62, *p* < 0.001, which derived mostly from between-study differences, while between-outcome differences were non-significant, *QM*(5)= = 1.11, *p* = 0.405. At single outcome level, there were small favorable effects for social support, SMD = 0.15, 95% CI [0.04, 0.25], and self-esteem, SMD = 0.23, 95% CI [0.10, 0.35], while no effects emerged for other outcome types. Residual heterogeneity was significant, *Q*(19) = 62.41, *p* < 0.001, but moderate across all outcome types (40.3 ≤ *I*^*2*^ ≥ 50.4).

Also for follow-up assessments, GRADE ratings^29^ indicated a very low certainty of evidence for all outcome categories (see Supplementary Data 14).

### Stability of intervention effects at follow-up assessment

Overall, there was an almost perfect stability of intervention effects from post-intervention to follow-up assessments, ICC = 0.88, 95% CI [0.86, 0.90]. Effect estimates showed moderate to good stability for mental distress, ICC = 0.63, 95% CI [0.53, 0.72], and resilience factors, ICC = 0.81, 95% CI [0.71, 0.88], and were even more stable for positive mental health, ICC = 0.94, 95% CI [0.92, 0.96].

### Moderator analyses

Based on the substantial to considerable heterogeneity identified by our main analyses mainly resulting from between-study differences, we performed several moderator analyses (see Table 2).

**Table 2:** Results of moderator analyses at post-intervention assessment.

	Mental distress		Positive mental health		Resilience factors	
	*n/k*	*M*(SMD) [95% CI], *p*	*n/k*	*M*(SMD) [95% CI], *p*	*n/k*	*M*(SMD) [95% CI], *p*
***Sociodemographic characteristics***						
Mean age	74/132	*QM*(1) = 1.98, *p* = 0.164	69/110	*QM*(1) = 1.67, *p* = .0.201	40/57	*QM*(1) = 0.00, *p* = 0.950
Gender (% women)	82/144	*QM*(1) = 0.00, *p* = 0.978	74/121	*QM*(1) = 0.17, *p* = .0.681	42/59	*QM*(1) = 0.49, *p* = 0.486
***Population type*** (Military vs. University/College vs. Workplace)						
Omnibus moderator test	55/98	*QM*(2) = 1.88, *p* = 0.163	47/72	*QM*(1) = 0.06, *p* = 0.811	32/41	*QM*(1) = 0.46, *p* = 0.638
***Delivery format*** (eHealth vs. mHealth vs. mixed)						
Omnibus moderator test	85/150	*QM*(2) = 2.18, *p* = 0.120	77/123	*QM*(2) = 2.27, *p* = 0.111	45/64	*QM*(1) = 1.51, *p* = 0.233
***Theoretical foundation*** (CBT vs. Coping Literature vs. Mindfulness vs. Positive Psychology vs. mixed)						
Omnibus moderator test	56/101	*QM*(4) = 1.19, *p* = 0.329	56/95	*QM*(4) = 0.50, *p* = 0.734	37/56	*QM*(4) = 0.84, *p* = .511
***Guidance***						
Unguided						0.24 [0.16, 0.34], *p* < 0.001
Guided						0.44 [0.25, 0.64], *p* < 0.001
Omnibus moderator test	84/148	*QM*(1) = 0.95, *p* = 0.332	77/123	*QM*(1) = 2.72, *p* = 0.103	45/64	*QM*(1) = 3.81, *p* = 0.058
***Intervention type*** (standalone vs. blended interventions)						
Omnibus moderator test	84/149	*QM*(1) = 0.26, *p* = 0.610	76/122	*QM*(1) = 0.02, *p* = 0.897	45/64	*QM*(1) = 0.20, *p* = 0.653
***Degree of individualization*** (individualized vs. standardized)						
Omnibus moderator test	85/150	*QM*(1) = 2.43, *p* = 0.123	77/123	*QM*(1) = 2.25, *p* = 0.138	45/64	*QM*(1) = 0.09, *p* = 0.769
***Intervention intensity***						
in weeks	82/142	*QM*(1) = 0.12, *p* = 0.729	77/123	*QM*(1) = 0.98, *p* = 0.325	45/64	*QM*(1) = 1.71, *p* = 0.198
***Improvement over time***						
Publication year	85/150	*QM*(1) = 0.86, *p* = 0.355	77/123	*QM*(1) = 3.23, *p* = 0.076	45/64	*QM*(1) = 0.39, *p* = 0.538
***Type of control group***						
No intervention/ waitlist		–0.30 [–0.39, –0.21], *p* < 0.001		0.38 [0.15, 0.60], *p* = 0.001		
Low-intensity active control		–0.31 [–0.45, –0.17], *p* < 0.001		0.22 [0.04, 0.40], *p* = 0.019		
High-intensity active control		–0.08 [–0.18, 0.01], *p* = 0.086		0.07 [–0.01, 0.15], *p* = 0.102		
Omnibus moderator test	85/150	*QM*(2) = 6.51, *p* = 0.002*	77/123	*QM*(2) = 4.16, *p* = 0.019*	45/64	*QM*(2) = 0.18, *p* = 0.835
***COVID-19 context*** (before COVID-19 vs. during COVID-19)						
Omnibus moderator test	85/150	*QM*(1) = 0.49, *p* = 0.488	77/123	*QM*(1) = 1.12, *p* = 0.293	45/64	*QM*(1) = 0.09, *p* = 0.763
***Small digital component*** (small digital component vs. other)						
Omnibus moderator test	84/149	*QM*(1) = 0.51, *p* = 0.479	76/122	*QM*(1) = 0.18, *p* = 0.675	45/64	*QM*(1) = 0.22, *p* = 0.641

*Note*. As results were at high risk of being biased by single studies, we did not report on moderation tests when three or less effect estimates were available per subgroup.

*QM*(*df*) omnibus test for moderators, which follows approximately a χ^2^ distribution, *df* degrees of freedom, *k* number of effect estimates, *SMD* standardized mean difference, *p* value, *95% CI* 95% confidence interval.

* highlights significant results at *p* < .05.

At post-intervention assessment for both mental distress and positive mental health, the type of control condition impacted effect estimates with favorable effects being larger for no intervention/waitlist controls and low-intensity controls than for high-intensity controls. There was a trend towards more favorable intervention effects for guided compared to unguided interventions for resilience factors, but no evidence for other moderator effects.

For follow-up assessments, the results of the moderator analysis are presented in Supplementary Data 15. For samples with higher mean age, there was evidence for more favorable effects on mental distress, *QM*(1) = 4.32, *p* = 0.045, and positive mental health, *QM*(1) = 7.59, *p* = 0.010. Moreover, favorable effects on positive mental health were larger for guided compared to unguided interventions, *QM*(1) = 5.21, *p* = 0.029. For positive mental health, there was a trend towards larger favorable effects when studies employed passive compared to active control groups, *QM*(2) = 2.94, *p* = 0.067. For resilience factors, there was evidence for more favorable effects for standardized compared to individualized interventions, *QM*(1) = 6.60, *p* = 0.021.

### Sensitivity analyses

We examined whether the use of smaller or larger *between-outcome correlations* (*ρ* = 0.40, *ρ* = 0.80) impacted on our results (see Supplementary Data 16). Neither for post- nor follow-up assessments were the results significantly different.

To account for a potential impact of *risk of bias* within studies, we re-ran our analyses limiting included studies to those with low risk of bias for the respective category of RoB2^30^. Neither at post-intervention nor follow-up assessment these analyses yielded contrary results. However, in some cases, previously significant effects were non-significant as those analyses were based on a smaller number of effect estimates (see Supplementary Data 17).

When we excluded one *outlier*^31^ from our analyses on positive mental health at post-intervention, which showed a strong favorable effect on self-reported resilience (SMD = 4.90), results remained largely unchanged, SMD = 0.21, 95% CI [0.13, 0.29], while heterogeneity decreased, *Q*(121) = 467.29, *p* < 0.001.

We found no evidence for differences between studies examining digital resilience interventions during the *COVID-19 pandemic* and pre-pandemic studies, *p* ≥ 0.281 (see Table 2 and Supplementary Data 15).

We examined whether effect estimates were different for studies with *small digital component* and found no evidence for a difference neither at post-intervention nor follow-up assessment, *p* ≥ 0.350 (see Table 2 and Supplementary Data 15).

As we found evidence for a *publication bias* in the analyses on resilience factors at post-intervention, we performed additional sensitivity analyses for this model. When we included only non-affirmative results in our meta-analyses, the analyses still yielded small favorable effects, SMD = 0.10, 95% CI [0.04, 0.15], suggesting that no amount of publication bias under the assumed model would suffice to shift the point estimate to null.

## Discussion

The present systematic review comprehensively summarized evidence on digital resilience interventions. We found small favorable effects of digital resilience interventions over control groups for all outcome types, that is, mental distress, positive mental health, and resilience factors, which remained stable at (mostly short-term) follow-up assessments and robust in sensitivity analyses. At post-intervention assessment, favorable intervention effects for mental distress and positive mental health were larger when studies used no intervention/waitlist controls and low-intensity active comparators than for high-intensity active comparators (i.e., with similar duration and attention). At follow-up assessments, a moderator effect of age indicated more favorable intervention effects on mental distress and positive mental health in older samples.

Our findings tie in with a recent review on online interventions to promote resilience^27^, which found small to moderate favorable effects, SMD = 0.54, 95% CI [0.28, 0.78], on self-reported resilience, but contradict results from Díaz-García et al. ^21^, who found no evidence for favorable effects on self-reported resilience, SMD = 0.12, 95% CI [–0.14, 0.38]. Beyond previous reviews, our systematic review relied on a much larger number of studies (101 in our analyses vs. 11^21^ vs. 22^27^) including more participants (20,010 participants in our analyses vs. 1174^21^ vs. 2876^27^). Moreover, by applying a state-of-the-art definition of resilience^1,5,9^, we examined a broader range of outcomes showing small favorable effects on mental distress, positive mental health (including self-reports of resilience), and resilience factors. Thereby, we provide a proof of concept showing that digital resilience interventions promote not only mental health but also resilience factors and likely, in turn, resilient outcomes (i.e., stable good mental health or a quick regain of mental health during or after stressor exposure). However, future high-quality effectiveness studies will have to further examine this mediating mechanism and also study why favorable effects were absent for active coping and self-efficacy as well-established resilience factors^1,32,33^.

Intervention effects were comparable in size to those (mostly) found for in-person interventions. For example, Kunzler et al. ^13,15^ reported small to moderate effects of primarily in-person resilience interventions in healthcare professionals (44 studies) and healthcare students (22 studies) on self-reported resilience and mental distress, while there were little to no effects on positive mental health. Similarly, Joyce et al. ^34^ reported small to moderate favorable effects of mostly in-person resilience interventions based on 11 studies. Numerically, effects on self-reported resilience in previous systematic reviews were larger (range of SMDs: 0.43–0.45)^13,15,34^ than those obtained by our analyses (SMDs = 0.22–0.23). However, based on Wald tests no significant differences emerged for both, self-reported resilience and mental distress. By contrast, effects on positive mental health were even larger and found more consistently than in previous reviews^13,15^. Due to the large number of studies included in our review, we were able to perform more analyses on follow-up effects than previous reviews^13,15^. For short to medium follow-up intervals (≤ 12 months), we found evidence for stable effects (ICC = 0.88) across all outcome categories. However, for single outcome types (e.g., mindfulness, mental health), effect estimates were smaller at follow-up assessment. A potential decrease of intervention effects over time should therefore be examined in future studies with longer follow-up periods. In sum, the current review provides evidence for digital resilience interventions having the potential to effectively promote resilience - potentially even in the long-term.

However, we also found that favorable effects at post-intervention assessments were smaller (and non-significant) for mental distress and positive mental health in the subgroup of studies using high-intensity active comparators (e.g., intense psychoeducation^35^, comparable unspecific narratives^36^). Thereby, our analyses were more nuanced than those of previous reviews that—at the most—contrasted passive and active controls^13,15^. This finding suggests that the favorable effects of the included interventions may (at least to some degree) result from unspecific effects of attention and/or engagement—an effect well-known from face-to-face interventions^9^. At the same time, one may ask for suitable comparators for digital resilience interventions. Even though active controls can be seen as the gold standard of intervention evaluation^37^, some of those comparators may have been (unintendedly) less intense resilience interventions. For example, many studies^38,39^ used psychoeducation as key component of their interventions, while other studies^35,40^ used psychoeducation as an active comparator. Moreover, in real-world settings, the most realistic comparator to digital resilience intervention is no intervention as most people have no access to resilience interventions (but see for a critical reflection on the validity of waitlist controls: Cuijpers et al.^37^). Thus, our finding of reduced intervention effects in studies using high-intensity comparators may also point to vague definition of resilience-promoting interventions, which might have also negatively impacted on the choice of comparators.

Interestingly, we found evidence for more favorable intervention effects on mental distress and positive mental health in older samples at follow-up assessment. This may reflect a larger proportion of older adults going on to use intervention components in their everyday life after the end of the intervention period (e.g., they use another meditation app after being included in a RCT on a mindfulness app). So far, there is only little evidence on the determinants of continuance intention for digital health interventions, however, previous studies showed that older adults use digital interventions more consequently^41^ and that the link between satisfaction with mHealth interventions and continuance intentions is particularly strong in older adults^42^. Moreover, older adults may be less ‘overdosed’ by the constant use of mobile and web-based services or take their participation in the study more seriously than younger samples (e.g., students who participate in a study to receive course credit^43^). Older adults may also, somewhat counterintuitively, make better use of digital tools and offers compared to younger people, as shown in the COVID-19 pandemic^44^. To note, our meta-analyses do not include many older people (highest mean age: 57.6 years^45^) and our findings apply to the age range from young to middle-aged adulthood. Future studies will have to examine age as effect modulator in greater detail also including older adults (≥ 60 years) and may determine additional variables that impact on age-related differences in intervention effects (e.g., eHealth literacy^46^, attitudes towards e/mHealth services^47^).

We found more favorable effects on resilience factors for standardized compared to individualized interventions at follow-up assessments. This finding was surprising at first sight and may partly be accounted for by the small number of studies included in our analyses employing individualized interventions. Moreover, individualized interventions that adapt intervention intensity, delivery and/or contents based on participants’ responses and behaviors may be more complex in terms of design and delivery. Thus, this finding may rather point to the potentials of individualized interventions being not used yet in the research on digital resilience interventions. Therefore, the result should not be misinterpreted as a valid evaluation of the impact of individualization on intervention effects. More high-quality studies examining the add-on effects of individualized interventions on a broad range of outcomes including acceptability^48^ and user engagement^49^, which may be more sensitive to individualization, are strongly needed.

Other moderator effects did not emerge consistently across outcomes. For example, our analyses on resilience factors at post-intervention assessment and on positive mental health outcomes at follow-up assessments pointed to an impact of guidance favoring guided over unguided interventions, however, the effect was only close-to-significance for resilience factors and did not emerge consistently between outcome types and over time. Future studies will have to explore these effects in greater detail.

Although included studies were rather heterogeneous with respect to intervention content, intervention delivery, formats and intensity, we found no evidence for moderator effects of these intervention characteristics. For some of these variables, low reporting standards in primary studies did not allow for more in-depth analyses. Future studies complying with higher reporting standards^50^ will help to shed light on these potential effect modulators. Such studies will allow us to derive more concrete recommendations on ideal intervention design and delivery.

The findings of the present review have to be interpreted in the light of their limitations, which arise from both, the included studies and the review process itself.

A major shortcoming in the field is that a precise definition of resilience interventions is still missing^9^. In line with two recent Cochrane reviews^13,15^, we included studies that either explicitly state to promote resilience (or resilience-related concepts like hardiness and stress-related/posttraumatic growth) or that refer to resilience as a key background of their intervention. However, even between two studies examining the same intervention^36,51^, the theoretical framing may differ, with interventions being referred to as either resilience or (mental) health promoting or focusing on the treatment of mental distress. Thus, there is a huge need for a more elaborated definition of resilience-promoting interventions which goes beyond authors’ labeling. As for most in-person resilience interventions^13,15^, overall risk of bias was moderate to high, and certainty of evidence was very low across all outcomes. This may reflect that high-quality research requires resources that are often not available for researchers designing and evaluating resilience interventions. However, such research is needed to validly examine intervention effects and tailor interventions to participants’ (likely) heterogeneous needs.

Other limitations arise from our review process. We searched five databases from 2019 to 2022 and identified studies published before 2019 by means of systematic reviews on resilience and health-promoting interventions (see Supplementary Data 2 for our search rationale). Moreover, we performed extensive citation searching. However, we cannot exclude that we have missed relevant studies. Moreover, we made minor changes from the preregistration of the review, which are described in Supplementary Data 1. In line with recommendations of the Cochrane collaboration^52^, we refrained from studying pre-to-post changes as pre-to-post value correlations were only available for a very small number of studies and the reliance on pre-to-post changes may also unintendedly ‘correct’ flaws in study design (like unsuccessful randomization)^53^. However, analyses based on the between-group comparison of pre-to-post changes may have provided divergent results (see Liu et al. ^26^ for a review on pre-to-post changes during resilience interventions). Moreover, we were not able to perform (component) network meta-analysis. Such analyses are highly needed to rank intervention components according to their efficacy and to derive recommendations for ideal interventions. In the case of our review, the requirements for network meta-analyses^54^ were not met (e.g., we found non-random differences between effect modifiers). However, future reviews based on more homogeneous studies may use the potential of network meta-analysis to shed further light on the relative importance of intervention components and to identify the “active ingredients” of those interventions^55^. These may include the promotion of higher-level resilience mechanisms (e.g., positive appraisal style^11^; regulatory flexibility^12^) or competencies like self-reflection^56^, with preliminary evidence suggesting that intervention effects of health-promoting interventions are mediated via positive appraisal style^57^. For regulatory flexibility, first training programs are about to be tested empirically^58,59^. Future studies will have to address these research gaps and compare the relative importance of different mechanisms.

Moreover, some of the included studies also aimed at targeting other outcomes than those included in the present review (e.g., fatigue and pain^45^, parental acceptance^60^, work engagement^61^). These outcomes were not considered in our analyses. Thus, our results only allow for conclusions on mental health and resilience factors and should not be misinterpreted as overall evaluation of the included interventions as they may be (more or less) effective in targeting other outcomes.

The present review provides preliminary evidence for the efficacy of digital interventions to enhance resilience. Evidence for favorable intervention effects was comparably strong (or weak) as for mostly non-digital interventions^13,15,26,34^. Favorable effects were found to be stable during short- and medium-time follow-up periods.

At the same time, we found substantial to considerable heterogeneity mostly coming from between-study differences and no strong evidence in favor of any specific digital resilience intervention. By contrast, most interventions were only examined in single trials with limited statistical power, which need replications. Thus, the current review should not be misunderstood as a prediction of concrete intervention effects of any resilience intervention, which is also indicated by wide prediction intervals that consistently included null effects.

Preparing for future crises in terms of resilience interventions would require more coordinated international research effort. One may learn from recent advances in the field of transdiagnostic psychosocial interventions for the treatment of mental distress in stress-exposed populations^62,63^. Initiated by the World Health Organization, a series of scalable psychosocial interventions (e.g., Problem Management Plus^64^, Step-by-Step^65^) was developed to address the high care need in stress-exposed populations. Most importantly, those interventions are examined in a series of high-quality RCTs for their feasibility and effectiveness^63,66^. Such an approach may also be useful for the development and evaluation of digital resilience interventions. The present review may provide an evidence base for intervention development, and later, individual-participant-data meta-analyses based on international effectiveness RCTs may help to shed light on the effectiveness of those interventions and participant-level effect modulators.

Digital interventions may also have the potential to improve health promotion and prevention in low-resource settings^67^, which resulted in a very optimistic initial view of digital interventions as potential ‘game changers’ in global health care^68^. So far, evidence on digital resilience interventions in low-resource settings is still rare with only 10.9% of the studies included in our review being conducted in middle-income countries and no intervention being delivered in a low-income country. While our review does not allow for strong conclusions on low-resource settings, implementation studies of other digital interventions in those settings pointed to substantial barriers^69^ (e.g., problems due to non-participative intervention development and delivery). Future research needs to examine whether and how digital resilience interventions can help to deliver mental health promotion and prevention in settings with limited resources^70^.

Moreover, the current review focused on digital resilience interventions in mostly middle-aged adult populations. However, preparing for future crises will need a lifespan approach also including children and adolescents as well as older people. Evidence on resilience interventions for those age groups is still rare^18,71,72^. Especially for children and adolescents, that were found to be particularly burdened by increases in stressor exposure^73,74^, digital resilience interventions may constitute an important component of stressor preparedness^75^. Future studies will have to examine whether resilience promotion in those age groups requires interventions that are more sensitive to developmental processes^76^.

The present review found small favorable effects of digital resilience interventions on mental distress, positive mental health, and resilience factors, which remained stable at least at short-term follow-up assessments. Those effects were comparable between online and mobile interventions and to those found for in-person interventions. For some but not all outcomes, we found older age to be associated with more favorable effects at follow-up assessments. So far, only a small number of studies made use of potential advantages of digital interventions (i.e., flexible time schedules, individualization) with mixed results. Digital resilience interventions have the potential to contribute to preparedness for future major disruptions. However, there is no strong evidence for any particular intervention, with the majority of interventions being only examined in single studies. Future research should focus on the evidence-based development of digital resilience interventions, which should be examined in fully powered effectiveness studies in both low- and high-resource settings. These studies may pave the way for digital resilience interventions being used to prepare and manage major disruptions at a societal level.

## Methods

This systematic review adheres to the standards of the Cochrane Collaboration^52^ and is reported according to the Preferred Reporting Items for Systematic Reviews and Meta-Analyses^77^ (PRISMA). Differences between the preregistration of the review (PROSPERO preregistration-ID: CRD42021286780) and the final review are presented as Supplementary Data 1.

### Search strategy

The search strategy was developed based on two previous Cochrane reviews^13,15,28^. First, we searched for systematic reviews examining health- and resilience-promoting interventions irrespective of their delivery mode. This approach was chosen to efficiently identify randomized-controlled trials (RCTs) that had been published between 2000 and 2018. Second, we searched for primary studies to cover the period from January 1, 2019 to August 15, 2022 (see Supplementary Data 2 for the rationale of this two-step search strategy). Searches were performed in the Cochrane Central Register of Controlled Trials (CENTRAL), Embase (incl. Pubmed and Medline), PsycINFO and PsycArticles via EbscoHost, Scopus, and Web of Science. For both searches, search terms comprised three clusters that were searched in title, abstract, and keywords: terms related to i) resilience and adaptation processes (e.g., “resilien*”, hardiness), ii) interventions (e.g., “psycho* intervention*”), and in case of our search for systematic reviews iii) terms related to systematic reviews (e.g., review*, meta-analys*), while our search for primary studies included iii) terms related to digital delivery mode (e.g., online, mobil*). Terms within one cluster were linked using the Boolean operator *OR*, while clusters were linked using the operator *AND*. If applicable, we used Medical Subject Headings (MeSH) and Emtree terms (for Embase). Search strategies are presented in Supplementary Data 2. Moreover, we checked the reference lists of included studies for potentially eligible primary studies.

### Search criteria

Eligible studies were (cluster) randomized controlled trails ([c]RCT) examining resilience-promoting interventions in adult non-clinical populations, that is, samples without a diagnosis of mental or developmental disorders, intellectual disability, or physical health conditions; or samples at risk for mental disorders or physical health conditions but scoring below clinical cut-off scores. Resilience-promoting interventions were psychosocial interventions that either aimed at promoting psychological resilience, hardiness, or stressor-related growth, mentioned those concepts as relevant theoretical background, or addressed those concepts as primary or secondary outcomes. Interventions were eligible irrespective of their content, duration, and setting. At least parts of the intervention were delivered digitally (i.e., online or mobile applications, computer-based interventions). In some cases, interventions were delivered offline (e.g., at a university computer) to ensure a highly standardized delivery. As these interventions could also been delivered using a personal computer or a mobile device, those studies were also eligible for inclusion. All types of comparators were eligible, that is, waitlist controls, care as usual, attention and active controls. Eligible studies needed to assess at least one outcome of the categories mental distress (i.e., general distress, symptoms of anxiety, depression and stress, posttraumatic stress), positive mental health (i.e., happiness, life satisfaction, mental health, stressor-related/posttraumatic growth, quality of life, positive emotions/affect, self-reported resilience, vitality, wellbeing), or resilience factors (i.e., active coping, mindfulness, optimism, self-compassion, self-efficacy, self-esteem, social support).

### Study selection

For both searches deduplication was performed in Zotero^78^. Systematic reviews were assessed independently by two reviewers (LvB, SKS) in Rayyan^79^ (*kappa* = 0.88). From eligible systematic reviews, all studies included in relevant systematic reviews were checked at full-text level independently by two raters (LvB, SKS) for eligibility using a standardized Excel form (*kappa* = 0.89). For our update search on primary studies, titles/abstracts and full texts were screened by two reviewers (LvB, LS, SKS) independently in Rayyan^79^. Interrater reliability was almost perfect at title/abstract level (*kappa* = 0.89) and full-text level (*kappa* = 0.85). At both stages of screening, disagreements were resolved through discussion or by consulting a third reviewer (AK, SKS).

### Data extraction

We developed a customized data extraction sheet for this review. All descriptive data of eligible primary studies were extracted by one reviewer and checked by a second (LvB, LS, SKS). Outcomes were extracted independently by two reviewers (LvB, LS, SKS). Any disagreements were resolved through discussion or consultation of a third reviewer (AK, SKS).

### Risk of bias

Two team members (LvB, LS) independently assessed the risk of bias of included primary studies using the Cochrane risk-of-bias tool for randomized trials (RoB2)^30^. RoB2 assesses the following bias domains: i) randomization process, ii) deviations from the intended interventions, iii) missing outcome data, iv) outcome measurement, and v) selection of reported results. For cRCTs, we additionally assessed risk of bias due to identification/recruitment of participants. Bias ratings were assessed at single outcome level and overall study level. Judgements could be “low” or “high” or express “some concerns”^30^.

### Publication bias

We examined a potential publication bias using visual inspections of (contour-enhanced) funnel plots^80^ and statistically by approximating rank correlation tests^81^. Those tests are available for multilevel models by including sampling error as moderator to the main analyses. If the sampling error significantly predicts effect estimates, this can be interpreted as evidence for a publication bias.

### Data synthesis

Included studies were summarized narratively and in tabular form. Pairwise meta-analyses were performed for primary outcomes in cases where more than three effect estimates were available per outcome type (e.g., depressive symptoms), and these were sufficiently homogeneous in terms of interventions and outcome assessment. Studies with more than two groups were rare in our analyses. In cases of multiple intervention arms, we determined which group was relevant for our review. Groups were averaged when two or more intervention groups were supposed to show equal intervention effects. In cases where add-ons were examined (e.g., including personalized feedback) and add-ons were supposed to be more effective, we included the group which was hypothesized to have the most favorable effect. In our main analyses, we included the control group showing the highest intensity for a more conservative approach (i.e., when a waitlist control and an active control group were available, we included the active control group in our main analyses). In cases where data needed for calculation of effect estimates were missing or unclear, primary study authors were contacted by the review team via email and sent one reminder email when we did not receive a response.

Meta-analyses were performed in *R* version 4.2.3^82^ using the packages *metafor*^83^, *clubSandwich*^84^, and *PublicationBias*^85^. All analyses used random-effects models and maximum likelihood estimations with an inverse variance method. Standardized mean differences (SMDs, Hedges’ *g*) at post-intervention and follow-up assessments were used as effect estimates and their 95% confidence intervals (CIs) as indicators of significance. SMDs were calculated based on means (*M*) and standard deviations (*SDs*), with positive SMDs indicating favorable intervention effects for positive mental health and resilience factors and unfavorable intervention effects for mental distress. To account for uncertainty of meta-analytical findings, we calculated 95% prediction intervals (PIs) as an estimate of the range in which 95% of future observations will fall^86^, when more than 10 effect estimates were available. Effect estimates of cRCTs were corrected for clustering effects^52^. As most cRCTs did not report on corrected standard errors, we used the formula 1 + (M -1)•ICC to estimate the design effect, with M being the average cluster size, and ICC the intra-cluster correlation^52^. In cases where no ICC was available for cRCTs, we used ICC = .05 as a mild conservative estimate^87^.

Our main analyses aimed at answering the question of whether there is an effect of digital resilience interventions on mental distress, positive mental health, and resilience factors. Due to qualitative differences between outcome categories, we calculated separate models for mental distress, positive mental health, and resilience factors at both post-intervention and follow-up assessments. Exploratively, we examined the stability of intervention effects between post-intervention and follow-up assessments by means of two-way random-effects intraclass correlations (ICCs)^88^. For our main analyses, we used a multilevel model nesting effect estimates within studies (outer factor) and outcome types (inner factor)^89^. As little information was available for between-outcome correlations within studies, covariances were imputed based on a correlation of *ρ* = 0.60, with other associations being used for sensitivity analyses^90^. For each model, we examined whether the use of an unstructured variance-covariance matrix improved model fit compared to structured matrices. As this was not the case for any model, symmetric matrices were used. Additionally, cluster-robust tests and CIs are reported to account for dependent effect estimates. Moreover, model fit and parameterization were examined by visual inspection of log-likelihood profiles, which were supposed to show single peaks.

Statistical heterogeneity was assessed using Cochran’s Q statistic^91^, with a significant Q statistic indicating the presence of heterogeneity. To quantify the amount of heterogeneity in our analyses, we used the *I*^*2*^ statistic (range: 0–100%) at a single outcome level, with values of 50% and above indicating substantial between-study heterogeneity^52^.

In our protocol, we planned several moderator analyses (PROSPERO preregistration-ID: CRD42021286780). Due to the substantial between-study heterogeneity in our primary analyses on all outcome categories, these analyses were performed for the main analyses on mental distress, positive mental health, and resilience factors. For categorical variables (e.g., type of control group) we used subgroup analyses, while meta-regressions were used for omnibus moderation tests and continuous moderators (e.g., publication year), with a significant *QM* statistic indicating the presence of a moderator effect. Moderator analyses were performed for sociodemographic sample characteristics (i.e., age, gender balance per sample, population type), delivery format (eHealth vs. mHealth vs. mixed), theoretical foundation (CBT vs. coping literature vs. mindfulness vs. positive psychology vs. mixed), availability of guidance (unguided vs. guided [i.e., availability of human guides or coaches who support intervention delivery]), intervention type (standalone vs. blended), individualization (standardized vs. individualized), intervention intensity in weeks, publication year (as proxy of improvements over time), and type of control group (passive controls [i.e., no intervention/waitlist] vs. low-intensity active controls vs. high-intensity active controls).

Sensitivity analyses were performed for other between-outcome correlations (*ρ* = 0.40, *ρ* = 0.80), risk of bias (only for bias domains with relevant between-study variation), exclusion of outliers, the presence of COVID-19 as a societal-level macro-stressor and the impact of interventions only comprising a relatively small digital component. Moreover, for analyses with evidence for a publication bias, we performed sensitivity analyses using an inverse probability weighting and robust estimations^92^. For this purpose, we calculated a worst-case meta-analysis with affirmative results (i.e., significant and positive results) being infinitely more likely to be published than non-affirmative findings.

The certainty of evidence for primary outcome categories at post-intervention and follow-up assessments was assessed in duplicate using Grading of Recommendations, Assessment, Development and Evaluations (GRADE)^29^.

### Supplementary information

Supplementary Material

## Data Availability

The corresponding author can be contacted to request access to the data supporting the findings of this study.

## Code availability

Code for all statistical analyses may be available upon reasonable request from the corresponding author.
