# Supplementary material for: Digital interventions to promote psychological resilience: a systematic review and meta-analysis
Source: NPJ Digit Med. 2024 Feb 8;7:30. doi: 10.1038/s41746-024-01017-8 (PMC10853230; doi:10.1038/s41746-024-01017-8)
Supplement: Supplementary file 1 — Supplementary Material [file 41746_2024_1017_MOESM1_ESM.docx]

**Supplementary Material**

related to ‘**Digital interventions to promote resilience: A systematic review and meta-analysis’**

Sarah K. Schäfer^1,2^, Lisa von Boros^1^, Lea M. Thomas^1^, Angela M. Kunzler^1,3^, Saskia Lindner^4^,
Friederike Koehler^1,5,6^, Tabea Werner^1,5^, Federico Zappalà^1^, Isabelle Helmreich^1^,
Michèle Wessa^1,5^, Klaus Lieb^1,4,^*, and Oliver Tüscher^1,4,7^*

^1^ Leibniz Institute for Resilience Research, Mainz, Germany

^2^ Department of Clinical Child and Adolescent Psychology and Psychotherapy, Technische Universität Braunschweig, Braunschweig, Germany

^3^  Institute for Evidence in Medicine, Medical Center – University of Freiburg, Faculty of Medicine, University of Freiburg, Freiburg, Germany

^4^ Department of Psychiatry and Psychotherapy, University Medical Center of Johannes Gutenberg University, Mainz, Germany

^5^  Department of Clinical Psychology and Neuropsychology, Institute for Psychology, Johannes Gutenberg University Mainz, Mainz, Germany

^6^ Centre of Excellence in Music, Mind, Body and Brain, University of Jyväskylä, Finland

^7^ Institute for Molecular Biology, Johannes Gutenberg University Mainz, Mainz, Germany

Correspondence related to this Supplementary Material to sarah.schaefer@lir-mainz.de.

Table of Contents

[Supplementary Data 1. Differences between protocol and review (PROSPERO ID: CRD42021286780) 2](#_Toc155802077)

[Supplementary Data 2. Search strategies per database 5](#_Toc155802078)

[Supplementary Data 3. Characteristics of included studies 12](#_Toc155802079)

[Supplementary Data 4. Risk of bias ratings 24](#_Toc155802080)

[Supplementary Data 5. Publication bias analyses – Funnel plots at post-intervention assessments 33](#_Toc155802081)

[Supplementary Data 6. Publication bias analyses – Funnel plots at follow-up assessments 34](#_Toc155802082)

[Supplementary Data 7. Forest plots for mental distress at post-intervention assessment 35](#_Toc155802083)

[Supplementary Data 8. Forest plots for positive mental health at post-intervention assessment 36](#_Toc155802084)

[Supplementary Data 9. Forest plots for resilience factors at post-intervention assessment 37](#_Toc155802085)

[Supplementary Data 10. GRADE ratings at post-intervention assessments 38](#_Toc155802086)

[Supplementary Data 11. Forest plots for mental distress at follow-up assessment 40](#_Toc155802087)

[Supplementary Data 12. Forest plots for positive mental health at follow-up assessment 41](#_Toc155802088)

[Supplementary Data 13. Forest plots for resilience factors at follow-up assessment 42](#_Toc155802089)

[Supplementary Data 14. GRADE ratings at follow-up assessments 43](#_Toc155802090)

[Supplementary Data 15. Moderator analyses for follow-up assessments 45](#_Toc155802091)

[Supplementary Data 16. Sensitivity analyses – part I: Other between-outcome correlations 47](#_Toc155802092)

[Supplementary Data 17. Sensitivity analyses – part II: Risk of bias 49](#_Toc155802093)

[Supplementary Data 18. References of studies included in this review 54](#_Toc155802094)

### Supplementary Data 1. Differences between protocol and review (PROSPERO ID: CRD42021286780)

**Table 1** Differences between protocol and final review

|  | **Protocol** | **Final review** |
| --- | --- | --- |
| Review design | no changes | |
| Review question | no changes | |
| Searches and search strategies | Search for systematic reviews and primary studies as two-step approach. | There were no changes with respect to our search strategy. In our preregistration, we solely uploaded exemplary search strategies for two databases. We now present the full search strategy for primary studies (see SM2). |
| Types of included studies | No changes with respect to population, exposure, comparisons | |
| Eligible outcomes | **Main outcomes**:  - Resilience, as measured by changes on specific resilience scales such as the Brief Resilience Scale (Smith et al., 2008)  - Mental health and well-being as measured by changes in the respective assessment scales (e.g., Patient Health Questionnaire 9, Kroenke et al., 2001)  - General psychological distress or general mental health  - Anxiety symptoms  - Depressive symptoms  - Stress or stress perceptions  - Well-being or quality of life (e.g., well-being, life satisfaction, (health-related) quality of life, vitality, vigor)  - Adverse events  **Additional outcomes:**  - Social support  - Optimism  - Sense of coherence  - Self-efficacy  - Active coping  - (Re-)appraisal  - Mindfulness  - Acceptance  - Self-compassion  - Self-esteem  - Hardiness  - Positive emotions | In our final manuscript,we no longer differentiate between main and additional outcomes (this has only been done in the first place due to the constrains imposed by PROSPERO). Based on our findings in primary studies, we derived three larger outcome categories, that is, mental distress, positive mental health and resilience factors. Outcomes that had been preregistered have been assigned to these categories.  **Mental distress:**  - Anxiety symptom  - Depressive symptoms  - General distress  - PTSD symptoms  - Stress symptoms  **Positive mental health:**  - Happiness  - Life satisfaction  - Mental health  - Positive emotions/affect  - Stress-related/posttraumatic growth  - Quality of life  - Resilience  - Vitality  - Wellbeing  **Resilience factors:**  - Active coping  - Mindfulness  - Optimism  - Self-compassion  - Self-efficacy  - Self-esteem  - Social support  Adverse events, (re-)appraisal and hardiness were examined in a small number of studies *k* ≥ 3) and were thus not included in our analyses. |
| Measure of effects | Standardized mean differences (SMD) or standardized mean changes (SMC) will be used as effect size measures. | We used SMDs at post-intervention and follow-up assessments as SMC could not be calculated due to missing information on pre-to-post-test correlations. Moreover, we refrained from analyzing SMC as these have been discussed to provide biased results for the meta-analysis of RCTs (Cuijpers et al., 2017). |
| Screening and data extraction | Screening was performed in EPPI-Reviewer. | Due to practical reasons (i.e., fees to obtain liscenses for team members), screening was performed in Rayyan. |
| Risk of bias (quality) assessment | no changes | |
| Data synthesis | Meta-analysis on change scores | Could not be performed due to missing data and methodological concerns (Cuijpers et al., 2017). |
|  | The statistical analyses will be performed in RStudio using metafor. | This has been done, we used the packages metafor (Viechtbauer, 2010) and clubSandwich (Pustejovsky & Pustejovsky, 2020). |
|  | Missing data on continuous covariates will be handled using multiple imputations. | Missing data on continuous moderators was very rare and we decided to employ a complete cases analysis in these cases. |
|  | - | There was no further specification of our meta-analysis models. Based on our data, we decided to use a multilevel model accounting for multiple outcomes being assessed within one study. Moreover, we account for between-outcome correlations and report on cluster-robust estimates to account for non-independent effect estimates. |
| Assessment of heterogeneity | First, we will visually inspect forest plots with effect sizes and their 95% CIs. Second, we will examine heterogeneity statistically using I², τ², χ² test, and 95% CI prediction intervals. | We visually inspected forest plots with effect estimates and their 95% CIs. Moreover, statistical heterogeneity was assessed using Cochran’s Q statistic. To quantify the amount of heterogeneity in our analyses, we used the *I^2^* statistic (range: 0–100%) at single outcome level , with values of 50% and above indicating substantial between-study heterogeneity. |
| Subgroup analyses | **Study level:**  - Year of publication   **Participant level:**  - Age (young adults, middle-aged adults, old adults)  - Gender balance (percentage of female participants)  - Participants’ psychological distress  **Intervention level:**  - Resilience only interventions vs. multicomponent interventions  - Intervention context  - Intervention setting (e.g., individual vs. group vs. combined)  - Type of eHealth/mHealth intervention (web-based, mobile-based, blended intervention)  - Theoretical background (e.g., cognitive-behavioural therapy vs. mindfulness-based vs. combined)  - Intervention providers (e.g., non-guided self-help vs. guided self-help vs. therapist delivered vs. combined)  - Training length (one vs. multiple session interventions) | We examined the following moderators:  - Mean age  - Gender  - Population type (military vs. university/college vs. workplace)  - Delivery format (eHealth vs. mHealth vs. mixed)  - Theoretical foundation (CBT vs. Coping Literature vs. Mindfulness vs. Positive Psychology vs. mixed)  - Guidance (guided vs. unguided)  - Intervention type (standalone vs. blended intervention)  - Degree of individualization (individualized vs. standardized)  - Intervention intensity in weeks  - Publication year  - Type of control group (no intervention/waitlist vs. low-intensity active control vs. high intensity active control)  Age groups could not be examined due to highly restricted age ranges. Also for psychological distress, data was insufficient to allow for a respective analysis. Moreover, we could not examine training length in terms of session number as session numbers were insufficiently reported. |
| Assessment of  publication bias | Possible asymmetry in funnel plots will be inspected visually and by means of Egger’s regression test. Moreover, we will use contour-enhanced funnel plots to investigate in which area potentially missing effect sizes will fall. | We examined a potential publication bias using visual inspections of (contour-enhanced) funnel plots and statistically by approximating rank correlation tests. Those tests are available for multilevel models by including sampling error as moderator to the main analyses. This approach has been chosen due to the multilevel structure of our data. |
| Sensitivity analyses | Not specified. | Analyses on:  - Other between-outcome correlations  - Risk of bias  - Outliers  - COVID-19 context  - Small digital component |

### Supplementary Data 2. Search strategies per database

**Rational for our search strategy**

For this review, we searched both systematic reviews and meta-analyses (2000–2018) on resilience- and health-promoting interventions as well as standard databases (from 2019 onwards) for eligible primary studies. This decision was based on preliminary literature searches performed for this review project. Those showed that especially in earlier publications, many titles, abstracts and keywords did not contain information on digital intervention delivery. Thus, a search in databases for primary studies might have resulted in a relevant number of missed eligible primary studies in case we had included a search cluster related to digital intervention delivery. However, not including such a cluster would have resulted in substantial problems as a previous Cochrane review of our group using a similar search strategy results in 24,000 hits after deduplication in 2016 (Kunzler et al., 2020a, 2020b). Hits per database had doubled since then. Thus, resources would have been insufficient to screen such a large number of studies. Therefore, we decided to combine a search for eligible primary studies based on systematic review and meta-analyses with a search for primary studies in databases for more recent publication years. Moreover, we performed extensive citation search. For systematic reviews, we applied a maximal sensitive search strategy to minimize the risk of missing eligible primary studies.

**Search strategy for primary studies**

**1. APA PsycNet (PsycInfo via EbsciHost)**

| **#** | **Query** |
| --- | --- |
| 1 | resilience, psychological |
| 2 | adaptability |
| 3 | emotional adjustment |
| 4 | coping behavior OR coping behaviour |
| 5 | posttraumatic growth |
| 6 | protective factors |
| 7 | TI ( post-traumatic growth or posttraumatic growth or stress-related growth ) OR AB (  post-traumatic growth or posttraumatic growth or stress-related growth ) |
| 8 | TI ( ( positiv* W1 (adapt* or adjust*) ) ) OR AB ( ( positiv* W1 (adapt* or adjust*) ) ) |
| 9 | TI ( ( (psychol* W1 (adapt* or adjust*)) ) ) OR AB ( ( (psychol* W1 (adapt* or adjust*)) ) ) |
| 10 | TI ( ( (resilien* or hardiness*) ) ) OR AB ( ( (resilien* or hardiness*) ) ) |
| 11 | TI ( ((cope or coping)) ) OR AB ( ((cope or coping)) ) |
| 12 | TI ( ((withstand* or overcom* or resist* or recover* or thriv* or adapt* or adjust* or bounc* back) W3 (stress* or trauma* or  advers*)) ) ) OR AB ( ((withstand* or overcom* or resist* or recover* or thriv* or adapt* or adjust* or bounc* back) W3 (stress* or trauma* or advers*)) ) ) |
| 13 | (S1 OR S2 OR S3 OR S4 OR S5 OR S6 OR S7 OR S8 OR S9 OR S10 OR S11 OR S12) |
| 14 | cognitive techniques |
| 15 | psychotherapy |
| 16 | psychotherapeutic techniques |
| 17 | relaxation therapy |
| 18 | mindful* |
| 19 | stress management |
| 20 | TI ( (psychotherap* or psycho-therap*) ) OR AB ( (psychotherap* or psycho-therap*) ) |
| 21 | TI ( (behav* W3 (intervention* or program* or therap*)) ) OR AB ( (behav* W3 (intervention*  or program* or therap*)) ) |
| 22 | TI ( (cognit* or cognitive behavior* or CBT) W3 (intervention* or program* or therap*)) )  OR AB ( (cognit* or cognitive behavior* or CBT) W3 (intervention* or program* or therap*)) ) |
| 23 | TI relaxation OR AB relaxation |
| 24 | TI mindful* OR AB mindful* |
| 25 | TI ( ( (counsel?ing or coaching) ) ) OR AB ( ( (counsel?ing or coaching) ) ) |
| 26 | TI ( ( (third wave W1 (psycho* or therap*)) ) ) OR AB ( ( (third wave W1 (psycho* or  therap*)) ) ) |
| 27 | TI cognit* restructur* OR AB cognit* restructur* |
| 28 | TI positive psychology OR AB positive psychology |
| 29 | TI ( (refram* or re-fram* or reapprais*) ) OR AB ( (refram* or re-fram* or reapprais*) ) |
| 30 | TI ( (stress W1 (inoculation or manag* or reduc* or resist*)) ) OR AB ( (stress W1  (inoculation or manag* or reduc* or resist*)) ) |
| 31 | TI (anxiety W3 manage*) OR AB (anxiety W3 manage*) |
| 32 | TI ( ( "acceptance and commitment" ) ) OR AB ( ( "acceptance and commitment" ) ) |
| 33 | multimodal treatment approach |
| 34 | TI ( ( (multimodal* or multi-modal* or combined modal*) ) ) OR AB ( ( (multimodal*  or multi-modal* or combined modal*) ) ) |
| 35 | health promotion |
| 36 | TI ( ( (health W3 (educat* or promot*)) ) ) OR AB ( ( (health W3 (educat* or promot*)) ) ) |
| 37 | TI ( ( (psycho* W3 (intervention* or program* or therap*)) ) ) OR AB ( ( (psycho* W3  (intervention* or program* or therap*)) ) ) |
| 38 | (TI ( ( (psycho* W3 (intervention* or program* or therap*)) ) ) OR AB ( ( (psycho* W3  (intervention* or program* or therap*)) ) )) AND (S14 OR S15 OR S16 OR S17 OR S18 OR S19 OR S20 OR S21 OR S22 OR S23 OR S24 OR S25 OR S26 OR S27 OR S28 OR S29 OR S30 OR S31 OR S32 OR S33 OR S34 OR S35 OR S36 OR S37) |
| 39 | TI ( ( (resilien* W5 (train* or program* or intervention* or promot* or prevent* or  enhanc* or learn* or teach* or educat* or increas* or develop* or manag* or therap* or protocol* or treat*)) ) ) AND AB ( ( (resilien* W5 (train* or program* or intervention* or promot* or prevent* or enhanc* or learn* or teach* or educat* or increas* or develop* or manag* or therap* or protocol* or treat*)) ) ) |
| 40 | TI ( ( (hardiness* W5 (train* or program* or intervention* or promot* or prevent* or  enhanc* or learn* or teach* or educat* or increas* or develop* or manag* or therap* or protocol* or treat*)) ) ) OR AB ( ( (hardiness* W5 (train* or program* or intervention* or promot* or prevent* or enhanc* or learn* or teach* or educat* or increas* or develop* or manag* or therap* or protocol* or treat*)) ) ) |
| 41 | S13 AND S38 |
| 42 | (S13 AND S38)) AND (S39 OR S40 OR S41) |
| 43 | TI "randomi?ed control* trial*" OR AB "randomi?ed control* trial*" |
| 44 | TI "control* clinical trial*" OR AB "control* clinical trial*" |
| 45 | TI randomi?ed |
| 46 | AB placebo* |
| 47 | AB randomly |
| 48 | AB trial |
| 49 | S43 OR S44 OR S45 OR S46 OR S47 |
| 50 | S42 AND S49 |
| 51 | online or internet or mobile or "app" or web or digital or ehealth or mhealth or "e-health" or  "m-health" or computer |
| 52 | S50 AND S51 (years: 2019 – 2022) |

**2. Cochrane Central Register of Controlled Trials (CENTRAL)**

| **#** | **Query** |
| --- | --- |
| 1 | [mh "Resilience, Psychological"] |
| 2 | [mh "social adjustment"] |
| 3 | [mh "Adaptation, Psychological"] |
| 4 | ("post-traumatic growth" or "posttraumatic growth" or "stress-related growth") |
| 5 | (positiv* near/1 (adapt* or adjust*)) |
| 6 | (psychol* near/1 (adapt* or adjust*)) |
| 7 | (resilien* or hardiness*) |
| 8 | (cope or coping) |
| 9 | ((withstand* or overcom* or resist* or recover* or thriv* or adapt* or adjust* or bounc* back) near/5 (stress* or trauma* or adversit*)) |
| 10 | {or #1-#9} |
| 11 | [mh psychotherapy] |
| 12 | MeSH descriptor: [Stress, Psychological] explode all trees and with qualifier(s): [therapy - TH] |
| 13 | (psychotherap* or psycho next therap*) |
| 14 | (behav* near/3 (intervention* or program* or therap*)) |
| 15 | ((cognit* or cognitive next behavior* or CBT) near/3 (intervention* or program* or therap*)) |
| 16 | (psycho* near/3 (intervention* or program* or therap*)) |
| 17 | relaxation |
| 18 | mindful* |
| 19 | (counsel*ing or coaching) |
| 20 | (third next wave next (psycho* or therap*)) |
| 21 | cognit* next restructur* |
| 22 | positive next psychology |
| 23 | (refram* or re next fram* or reapprais*) |
| 24 | (stress near/1 (inoculation or manag* or reduc* or resist*)) |
| 25 | (anxiety near/3 manage*) |
| 26 | "acceptance and commitment" |
| 27 | [mh "Combined Modality Therapy"] |
| 28 | (multimodal* or multi next modal* or combined modal*) |
| 29 | [mh "health promotion"] |
| 30 | (health near/3 (educat* or promot*)) |
| 32 | {or #11-#30} |
| 33 | (online or mobil or "app" or internet or web or digital or ehealth or mhealth or "e-health" or "m-health" or computer) |
| 33 | #10 and #31 |
| 34 | #33 and #32 (with publication year from 2019 to 2022, in Trials) |

**3. Embase.com including PubMed and Medline**

| **#** | **Query** |
| --- | --- |
| 1 | ((resilien*:ab,ti OR hardiness*:ab,ti OR 'post traumatic growth':ab,ti OR 'posttraumatic growth':ab,ti OR 'stress related growth':ab,ti OR ((positiv* NEAR/1 (adapt* OR adjust*)):ab,ti) OR ((psychol* NEAR/1 (adapt* OR adjust*)):ab,ti) OR (((withstand* OR overcom* OR resist* OR recover* OR thriv* OR adapt* OR adjust* OR 'bounc* back') NEAR/1 (stress* OR trauma* OR advers*)):ab,ti)) AND (psychotherap*:ab,ti OR 'psycho therap*':ab,ti OR cbt:ab,ti OR ((behav* NEAR/3 (intervention* OR program* OR therap*)):ab,ti) OR (((cognit* OR 'cognitive behavior*' OR cbt) NEAR/3 (intervention* OR program* OR therap*)):ab,ti) OR ((psycho* NEAR/3 (intervention* OR program* OR therap*)):ab,ti) OR ((stress NEAR/3 (inoculat* OR manag* OR reduc* OR resist*)):ab,ti) OR ((anxiety NEAR/1 manag*):ab,ti) OR relaxation:ab,ti OR mindful*:ab,ti OR counsel*ing:ab,ti OR coaching:ab,ti OR 'third wave':ab,ti OR refram*:ab,ti OR 're fram*':ab,ti OR 'cognitive restructur*':ab,ti OR 'positive psychology':ab,ti OR (((multimodal* OR 'multi modal*' OR 'combined modal*') NEAR/3 (treat* OR therap* OR intervention* OR program*)):ab,ti) OR ((health NEAR/3 (educat* OR promot*)):ab,ti) OR 'acceptance and commitment':ab,ti) OR (((resilience OR hardiness) NEAR/3 (train* OR program* OR intervention* OR promot* OR prevent* OR enhanc* OR learn* OR teach* OR educat* OR increas* OR develop* OR manag* OR therap* OR protocol* OR treat*)):ab,ti)) AND ([embase]/lim OR [embase classic]/lim) |
| 2 | 'randomized controlled trial'/exp OR 'randomized controlled trial' |
| 3 | 'controlled clinical study'/de |
| 4 | #2 OR #3 |
| 5 | random*:ti,ab |
| 6 | 'randomization'/de |
| 7 | 'intermethod comparison'/de |
| 8 | placebo:ti,ab |
| 9 | compare:ti OR compared:ti OR comparison:ti |
| 10 | (evaluated:ab OR evaluate:ab OR evaluating:ab OR assessed:ab OR assess:ab) AND (compare:ab OR compared:ab OR comparing:ab OR comparison:ab) |
| 11 | (open NEXT/1 label):ti,ab |
| 12 | ((double OR single OR doubly OR singly) NEXT/1 (blind OR blinded OR blindly)):ti,ab |
| 13 | 'double blind procedure'/de |
| 14 | (parallel NEXT/1 group*):ti,ab |
| 15 | crossover:ti,ab OR 'cross over':ti,ab |
| 16 | \| ((assign* OR match OR matched OR allocation) NEAR/5 (alternate OR group OR groups OR intervention OR interventions OR patient OR patients OR subject OR subjects OR participant OR participants)):ti,ab \| \| --- \| |
| 17 | assigned:ti,ab OR allocated:ti,ab |
| 18 | (controlled NEAR/7 (study OR design OR trial)):ti,ab |
| 19 | volunteer:ti,ab OR volunteers:ti,ab |
| 20 | 'human experiment'/de |
| 21 | trial:ti |
| 22 | #5 OR #6 OR #7 OR #8 OR #9 OR #10 OR #11 OR #12 OR #13 OR #14 OR #15 OR #16 OR #17 OR #18 OR #19 OR #20 OR #21 |
| 23 | ((random* NEXT/1 sampl* NEAR/7 ('cross section*' OR questionnaire* OR survey OR surveys OR database OR databases)):ti,ab) NOT ('comparative study'/de OR 'controlled study'/de OR 'randomised controlled':ti,ab OR 'randomized controlled':ti,ab OR 'randomly assigned':ti,ab) |
| 24 | 'cross-sectional study'/de NOT ('randomized controlled trial'/de OR 'controlled clinical study'/de OR 'controlled study'/de OR 'randomised controlled':ti,ab OR 'randomized controlled':ti,ab OR 'control group':ti,ab OR 'control groups':ti,ab) |
| 25 | 'case control*':ti,ab AND random*:ti,ab NOT ('randomised controlled':ti,ab OR 'randomized controlled':ti,ab) |
| 26 | 'systematic review':ti NOT (trial:ti OR study:ti) |
| 27 | nonrandom*:ti,ab NOT random*:ti,ab |
| 28 | 'random field*':ti,ab |
| 29 | ('random cluster' NEAR/3 sampl*):ti,ab |
| 30 | review:ab AND review:it NOT trial:ti |
| 31 | 'we searched':ab AND (review:ti OR review:it) |
| 32 | 'update review':ab |
| 33 | (databases NEAR/4 searched):ab |
| 34 | (rat:ti OR rats:ti OR mouse:ti OR mice:ti OR swine:ti OR porcine:ti OR murine:ti OR sheep:ti OR lambs:ti OR pigs:ti OR piglets:ti OR rabbit:ti OR rabbits:ti OR cat:ti OR cats:ti OR dog:ti OR dogs:ti OR cattle:ti OR bovine:ti OR monkey:ti OR monkeys:ti OR trout:ti OR marmoset*:ti) AND 'animal experiment'/de |
| 35 | 'animal experiment'/de NOT ('human experiment'/de OR 'human'/de) |
| 36 | #23 OR #24 OR #25 OR #26 OR #27 OR #28 OR #29 OR #30 OR #31 OR #32 OR #33 OR #34 OR #35 |
| 37 | #22 NOT #36 |
| 38 | #1 AND #37 |
| 39 | online OR 'app' OR internet OR mobil* OR web OR digital OR ehealth OR mhealth OR 'e-health' OR 'm-health' OR computer |
| 40 | #38 AND #39 AND (2019:py OR 2020:py OR 2021:py) |

**4. Scopus.com**

| **#** | **Query** |
| --- | --- |
| 1 | ( TITLE-ABS-KEY ( online OR internet OR "app" OR mobile OR web OR digital OR ehealth OR mhealth OR "e-health" OR "m-health" OR computer ) ) AND ( ( ( TITLE-ABS-KEY ( ( ( resilience OR hardiness ) AND ( train* OR program* OR intervention* OR promot* OR prevent* OR enhanc* OR learn* OR teach* OR educat* OR increas* OR develop* OR manag* AND ortherap* OR protocol* OR treat* ) ) ) ) OR ( ( ( TITLE-ABS- KEY ( ( resilien* OR hardiness* ) ) ) OR ( TITLE-ABS-KEY ( ( "post traumatic growth" OR "posttraumatic growth" OR "stress related growth" ) ) ) OR  ( TITLE-ABS-KEY ( ( positiv* W/1 ( adapt* OR adjust* ) ) ) ) OR ( TITLE-ABS- KEY ( ( psychol* W/1 ( adapt* OR adjust* ) ) ) ) OR ( TITLE-ABS- KEY ( ( ( withstand* AND overcom* OR resist* OR recover* OR thriv* OR adapt* OR adjust* OR "bounc* back" ) AND ( stress* OR trauma* OR advers* ) ) ) ) ) AND ( ( TITLE-ABS-KEY ( ( ( psychotherap* OR "psycho therap*" ) OR cbt OR mindful* OR ( behav* W/3 ( intervention* OR program* OR therap* ) ) OR ( ( cognit* OR "cognitive behavior*" OR cbt ) W/3 ( intervention* OR program* OR therap* ) ) OR ( psycho* W/3  ( intervention* OR program* OR therap* ) ) ) ) ) OR ( TITLE-ABS- KEY ( ( stress W/1 ( inoculat* OR manag* OR reduc* OR resist* ) ) ) ) OR ( TITLE-ABS-KEY ( ( ( anxiety AND n/1 AND manag* ) OR relaxation OR mindful* OR counsel*ing OR coaching OR "third wave" OR refram* OR "re fram*" OR "cognitive restructur*" OR "positive psychology" ) ) ) OR ( TITLE- ABS-KEY ( ( "acceptance and commitment" ) ) ) OR ( TITLE-ABS- KEY ( ( ( multimodal* OR "multi modal*" OR "combined modal*" ) W/3 ( treat* OR therap* OR intervention* OR program* ) ) ) ) OR ( TITLE-ABS- KEY((health W/3 (educat* OR promot*))))))) AND ( ( INDEXTERMS ( "clinical trials" OR "clinical trials as a topic" OR "randomized controlled trial" OR "Randomized Controlled Trials as Topic" OR "controlled clinical trial" OR "Controlled Clinical Trials" OR "random allocation" OR "Double-Blind Method" OR "Single-Blind Method" OR "Cross-Over Studies" OR "Placebos" OR "multicenter study" OR "double blind procedure" OR "single blind procedure" OR "crossover procedure" OR "clinical trial" OR "controlled study" OR "randomization" OR "placebo" ) ) OR ( TITLE-ABS-KEY ( ( "clinical trials" OR "clinical trials as a topic" OR "randomized controlled trial" OR "Randomized Controlled Trials as Topic" OR "controlled clinical trial" OR "Controlled Clinical Trials as Topic" OR "random allocation" OR "randomly allocated" OR "allocated randomly" OR "Double- Blind Method" OR "Single-Blind Method" OR "Cross-Over Studies" OR "Placebos" OR "cross-over trial" OR "single blind" OR "double blind" OR "factorial design" OR "factorial trial" ) ) ) OR ( TITLE-ABS ( clinical AND trial* OR trial* OR rct* OR random* OR blind* ) ) ) ) AND ( LIMIT- TO ( PUBYEAR , 2019 ) OR LIMIT-TO ( PUBYEAR , 2022 ) ) |

**5. Web of Science**

| **#** | **Query** |
| --- | --- |
| 1 | TS=(resilien* or hardiness*) |
| 2 | TS=(("post traumatic growth" or "posttraumatic growth" or "stress related growth")) |
| 3 | TS=((psychol* near/1 (adapt* or adjust*))) |
| 4 | TS=((positiv* near/1 (adapt* or adjust*))) |
| 5 | TS=((withstand* or overcom* or resist* or recover* or thriv* or adapt* or adjust* or "bounc* back" ) near/1 (stress* or trauma* or advers*)) |
| 6 | #1 OR #2 OR #3 OR #4 OR #5 |
| 7 | TS =((psychotherap* or "psycho therap*") or CBT or mindful* or (behav* near/3 (intervention* or program* or therap*)) OR ((cognit* or "cognitive behavior*" or CBT) near/3 (intervention* or program* or therap*)) OR (psycho* Edit Edit near/3 (intervention* or program* or therap*))) |
| 8 | TS=(stress near/3 (inoculat* or manag* or reduc* or resist*)) |
| 9 | TS=((anxiety near/1 manag*) or relaxation or mindful* or counsel*ing or coaching or "third wave" or refram* or "re fram*" or "cognitive restructur*" or "positive psychology") |
| 10 | TS= ((multimodal* or "multi modal*" or "combined modal*") NEAR/3 (treat* or therap* or intervention* or program*)) |
| 11 | TS=(health near/3 (educat* or promot*)) |
| 12 | TS=("acceptance and commitment") |
| 13 | #12 OR #11 OR #10 OR #9 OR #8 OR #7 |
| 14 | #13 AND #6 |
| 15 | TS=((resilience or hardiness) near/3 (train* or program* or intervention* or promot* or prevent* or enhanc* or learn* or teach* or educat* or increas* or develop* or manag* or therap* or protocol* or treat*)) |
| 16 | #15 AND #14 |
| 17 | TS=(randomised OR randomized OR randomisation OR randomisation OR placebo* OR (random* AND (allocat* OR assign*)) OR (blind* AND (single OR double OR treble OR triple))) |
| 18 | #16 and #17 |
| 19 | TS=((online or "app" or internet or mobil* or web or digital or ehealth or mhealth or "e-health" or "m-health" or computer) ) |
| 20 | #19 and #18 |
| 21 | #20 AND and #18 and 2021 or 2020 or 2019 (Publication Years) |

**Search strategy for systematic reviews on resilience-promoting interventions** (exemplary for Web of Science, all other search strings were added accordingly):

| **#** | **Query** |
| --- | --- |
| 1 | TS=(resilien* or hardiness*) |
| 2 | TS=(("post traumatic growth" or "posttraumatic growth" or "stress related growth")) |
| 3 | TS=((psychol* near/1 (adapt* or adjust*))) |
| 4 | TS=((positiv* near/1 (adapt* or adjust*))) |
| 5 | TS=((withstand* or overcom* or resist* or recover* or thriv* or adapt* or adjust* or "bounc* back" ) near/1 (stress* or trauma* or advers*)) |
| 6 | #1 OR #2 OR #3 OR #4 OR #5 |
| 7 | TS =((psychotherap* or "psycho therap*") or CBT or mindful* or (behav* near/3 (intervention* or program* or therap*)) OR ((cognit* or "cognitive behavior*" or CBT) near/3 (intervention* or program* or therap*)) OR (psycho* Edit Edit near/3 (intervention* or program* or therap*))) |
| 8 | TS=(stress near/3 (inoculat* or manag* or reduc* or resist*)) |
| 9 | TS=((anxiety near/1 manag*) or relaxation or mindful* or counsel*ing or coaching or "third wave" or refram* or "re fram*" or "cognitive restructur*" or "positive psychology") |
| 10 | TS= ((multimodal* or "multi modal*" or "combined modal*") NEAR/3 (treat* or therap* or intervention* or program*)) |
| 11 | TS=(health near/3 (educat* or promot*)) |
| 12 | TS=("acceptance and commitment") |
| 13 | #12 OR #11 OR #10 OR #9 OR #8 OR #7 |
| 14 | #13 AND #6 |
| 15 | TS=((resilience or hardiness) near/3 (train* or program* or intervention* or promot* or prevent* or enhanc* or learn* or teach* or educat* or increas* or develop* or manag* or therap* or protocol* or treat*)) |
| 16 | #15 AND #14 |
| 17 | TS=(meta-analys* OR systematic OR review*) |
| 18 | #16 and #17 |
| 19 | #17 AND #16 and Review Articles (Exclude – Document Types) and 1999 or 1998 or 1997 or 1996 or 1995 or 1994 or 1993 or 1992 or 1991 or 1990 or 1989 (Exclude – Publication Years) |
| 20 | #19 and and Psychiatry or Public Environmental Occupational Health or Psychology Clinical or Psychology Multidisciplinary or Psychology or Psychology Developmental or Health Care Sciences Services or Edit Social Sciences Interdisciplinary or Social Work or Nursing or Education Educational Research |

### Supplementary Data 3. Characteristics of included studies

**Table 2**. Characteristics of included studies

| **Study ID** | **Country** | **Population [type; age (M ± SD); % women]** | **n_randomized_; n_IG_; n_CG_** | **Intervention name** | **Theoretical basis** | **Delivery** | **Intensity of intervention** | **Comparator** | **Outcomes eligible for review** |
| --- | --- | --- | --- | --- | --- | --- | --- | --- | --- |
| Abbott 2009 | Australia^HI^ | sales manager; 40.5 yrs ± 9.45; 13.21% | 53; IG: 26; CG: 27 | resilience online (ROL program) | not specified | online | 7 modules | waitlist | quality of life, depressive symptoms, anxiety symptoms, stress symptoms |
| Aboody 2020^FU^ | Israel^HI^ | students; 23.51 yrs ± 1.45; 100% | 90; IG: 48; CG: 42 | GGtude Body Image (GGBI) | CBT | mobile (and lab visits) | 14 days with 4 levels per day | waitlist | depressive symptoms, self-esteem |
| Aikens 2014 | USA^HI^ | Dow Chemical Company employees; 18-65 yrs; no information | 89; IG: 44; CG: 45 | online mindfulness intervention | mindfulness | online | 7 sessions of 1 hour | waitlist | mindfulness, perceived stress, trait-resilience, vigor |
| Al-Refae 2021 | Canada^HI^ | no specific population; 25.24 yrs ± 8.74; 78.8% | 245; IG: 127; CG: 118 | Serene | mixed (CBT; mindfulness) | mobile | 4 weeks | waitlist | depressive symptoms, anxiety symptoms, stress symptoms, self-compassion, mindfulness, self-acceptance |
| Aminoff 2021 | Sweden^HI^ | no specific population; 42.7 yrs ± 17.4; 71.2% | 52; IG: 26; CG: 26 | iCBT Group (internet-delivered CBT) | CBT | online | 7 weeks with module per week | waitlist | depressive symptoms, quality of life, generalized anxiety symptoms, posttraumatic stress, perceived stress |
| Atad 2021 | Israel^HI^ | Israeli undergraduate Students; 26.5 yrs ± 5.8; 80.9% | 178; IG: 88; CG: 90 | solution–focused cognitive–behavioral (SFCB) coaching plus lecture | CBT | online | 13 weeks in-person lecture plus 12 weeks online coaching | active control | resilience, general distress |
| Auyeung 2019 | China^UMI^ | students; 22.82 yrs ± 3.36; 73% | 139; IG: 70; CG: 69 | Positive Psychological Intervention (PPI) | positive psychology | online | 6 days with 10-15-min sessions per day | active control | positive emotions/affect, depressive symptoms |
| Ayers 2015 | UK^HI^ | postnatal mothers; no information; 100% | 80; IG: 40; CG: 40 | brief online self-help exercise | CBT | online | 1 session | active control | positive emotions/affect |
| Ahmad 2021 | Turkey^UMI^ | students; 20.02 yrs ± 1.15; 68.3% | 60; IG: 30; CG: 30 | psychoeducational stress management program | Roy's adaptation model | online | 7 sessions in 6 weeks | waitlist | stress symptoms, active coping |
| Behrendt 2023^a^ | Germany^HI^ | working adults with elevated stress; 40.1 yrs ± 12.9; 73.30% | 30; IG: 15; CG: 15 | RESIST (online and app-supported resilience training) | CBT | online and mobile | 6 weeks with 1 module per week | waitlist | stress symptoms, depressive symptoms, resilience, self-efficacy, internal control, optimism, self-compassion, social support |
| Ben-Avraham 2022 | Israel^HI^ | Israeli Defense Force (IDF) soldiers; 19.06 yrs ± 0.58; 62.09% | 153; IG: 74; CG: 79 | mobile cognitive control training (CCT) | cognitive control | mobile | 14 days with 1 session per day | active control | resilience, self-efficacy, general distress, anxiety symptoms, quality of life |
| Beyer 2010 | USA^HI^ | students; 21.9 yrs ± 6.6; 83.4% | 163; IG: 123; CG: 40 | Emotional disclosure writing | written emotional disclosure | online | 3 sessions over 7-10 days | active control | general distress, PTSD symptoms, posttraumatic growth |
| Birk 2019 | Canada^HI^ | no specific population; 35.3 yrs ± 11.5; 48.65% | for analysis: 259; IG: 124; CG: 135 | attention bias modification training | attention bias modification | online | 1 session | active control | Anxiety symptoms |
| Bolier 2014^FU^ | Netherlands^HI^ | healthcare: nurses and allied HCP; 40 yrs ± 11.9; 79.8% | 1140; IG: 579; CG: 561 | Workers' health surveillance (WHS) module | mixed (CBT, mindfulness, positive psychology) | online | depending on intervention: between 4 and 12 weeks, in most cases 30mins per week | waitlist | mental health, wellbeing, depressive symptoms, anxiety symptoms |
| Bouchard 2012 | Canada^HI^ | soldiers; 24.9 yrs ± 5.55; 0% | 59; IG: 29; CG: 30 | ImPACT | CBT | online | brief introduction/refresher to stress management training plus 3 sessions over 5 days | no intervention | self-efficacy |
| Brog 2021 | German-speaking countries^HI^ | German speaking adults having at least mild depressive symptoms; 40.36 yrs ± 14.59; 81.3% | 107; IG: 53; CG: 54 | resilience and optimism during COVID-19 (ROCO) | CBT | online | 3 weeks with 6 modules à 40-80 min (2 modules / week) | care as usual | depressive symptoms, general distress, quality of life, optimism, resilience, self-efficacy |
| Burger 2015 | USA^HI^ | nursing students; NR; 82.7% | 52; IG: 28; CG: 24 | mindfulness meditation | mindfulness | online | introduction + 4 weeks, 10 min daily | waitlist | stress symptoms, mindfulness |
| Catuara-Solarz 2022 | UK^HI^ | adults with elevated mental distress; 39.99 yrs ± 6.109; 53.68% | 190; IG: 95; CG: 95 | Foundations | mixed (CBT; mindfulness) | mobile | 4 weeks (2 weeks of structured activities, 2 weeks of free use) | waitlist | anxiety symptoms, resilience, wellbeing, stress symptoms |
| Cavanagh 2018 | UK^HI^ | university staff and students; 31.03 yrs ± 11.64; 80% | 103; IG: 53; CG: 50 | mindfulness meditation | mixed (CBT; mindfulness) | online | 2 weeks | waitlist | mindfulness, perceived stress symptoms, general distress |
| Champion 2018^FU^ | UK^HI^ | no specific population; 39.13 yrs ± 5.02; 59.7% | 74; IG: 38; CG: 36 | Headspace | mindfulness | online | 30 days with daily 10-20-min sessions | waitlist | life satisfaction, perceived stress, trait-resilience |
| Chang 2022 | USA^HI^ | college students; 21.3 yrs ± 4.8; 71.6% | 678; IG: 326; CG: 352 | Isha Upa Yoga | mindfulness | online | 12 weeks of daily Yoga practice | waitlist | stress symptoms, wellbeing, anxiety symptoms, depressive symptoms, resilience, positive emotions/affect |
| Chilver 2021^FU^ | Australia^HI^ | students; 19.7 yrs ± 3.2; 70% | 409; IG: 205; CG: 204 | positive psychological intervention | mixed (positive psychology, mindfulness) | mobile | 7 weeks with 3 modules for 2 weeks each | active control | Mental wellbeing, life satisfaction, stress, depressive symptoms, anxiety symptoms, self-compassion, resilience |
| Cieslak 2016 | Poland^HI^ | health and human service professionals; 37.49 yrs ± 10.39; 78% | 168; IG: 87; CG: 81 | Self-efficacy intervention | CBT | online | 4 weeks x 1 module | active control | (secondary) PTSD symptoms, (secondary) posttraumatic growth, self-efficacy |
| Decker 2020 | Kenya^LMI^ | women at risk for or experiencing intimate partner violence; 26.57 yrs ± 4.7; 100% | 312; IG: 157; CG: 155 | MyPlan Kenya app | social cognitive theory, empowerment, trauma-informed care | mobile | unclear | care as usual | resilience, depressive symptoms, self-efficacy |
| Ebert 2021 | Germany^HI^ | working population in small and medium sized companies; 41.76 yrs ± 10.09; 76.3% | 396; IG: 198; CG: 198 | GET.ON Stress | CBT | mobile | 7 weeks with 1 session per week (plus optional modules), after 4 weeks: 1 booster session | care as usual | stress symptoms, depressive symptoms, resilience, well-Being |
| Enrique 2018^FU^ | Spain^HI^ | mostly students and administrative personnel from two universities; 23.8 yrs ± 3.85; 65.40% | 81; IG: 40; CG: 41 | Best Possible Self (e-BPS) | positive psychology, positive future thinking | computer-based and online | 1 session in laboratory, then online usage at least 5 min a day for one month | active control | optimism, positive emotions/affect, depressive symptoms |
| Eriksson 2018 | Sweden^HI^ | Psychologists; 36.2 yrs ± 8.2; 96.04% | 101; IG: 52; CG: 49 | mindful self-compassion training | mindfulness | online | 6 weeks with 6 15-min sessions per week | waitlist | mindfulness, self-compassion, perceived stress |
| Fassnacht 2022 | Australia^HI^ | students; 30.65 yrs ± 10.1; 81.1% | 215; IG: 126; CG: 89 | be well plan program | mindfulness, CBT | online | 5 weeks x one 2-hour session per week | waitlist | wellbeing, resilience, depressive symptoms, anxiety symptoms, self-efficacy |
| Feinberg 2020 | USA^HI^ | heterosexual military couples expecting their first child; 31.11 yrs ± 5.37; 50% | 112 (56 couples); IG: 58 (29 couples); CG: 54 (27 couples) | family foundations | group discussion approach | online | unclear | no intervention | depressive symptoms |
| Flett 2020^FU^ | New Zealand^HI^ | students; 17.87 yrs ± 0.47; 67.6% | 250; IG: 124; CG: 126 | Headspace | mindfulness | mobile | 3 months of app access with 6 reminder messages | waitlist | stress symptoms, resilience, self-efficacy, mindfulness |
| Flett 2019^FU^ | New Zealand^HI^ | students; 20.08 yrs ± 2.8; not reported | 210; IG: 135 (72 + 63); CG: 75 | Headspace/ Smiling Mind | mindfulness | mobile | 10 min each day for 10 days | active control | depressive symptoms, anxiety symptoms, stress, resilience, mindfulness |
| Gollwitzer 2018 | Germany^HI^ | healthcare providers; 40.22 yrs ± 10.18; 82% | 129; IG: 82 (41 + 41); CG: 47 | mental contrasting intervention | mental contrasting | online | 1 session, 3 weeks on daily basis | no intervention | stress symptoms |
| Grabbe 2020^FU^ | USA^HI^ | nurses; 45.24 yrs ± 13.19; not reported | 196; IG: 99; CG: 97 | community resiliency model (CRM) training plus “ichill” app | mindfulness | blended | 1 3-hrs session plus one year app use | active control | wellbeing, resilience, (secondary) PTSD symptoms |
| Hannibal | Germany^HI^ | working adults | 200; IG: 100. CG: 100 | Resilience training | CBT | online | 6 modules x 60-90 min | waitlist with active component | stress symptoms, depressive symptoms, resilience, optimism, self-efficacy, social support |
| Harrer 2018^FU^ | Germany^HI^ | students; 24.1 yrs ± 4.1; 74.7% | 150; IG: 75; CG: 75 | StudiCare Stress from Get.On | CBT | online and mobile | 5-7 weeks with 8 modules (30 to 90 minutes per module) | waitlist | stress symptoms, depressive symptoms, anxiety symptoms, wellbeing, resilience, self-compassion, self-esteem |
| Harrer 2021^FU^ | Germany^HI^ | students of a large German distance-learning university; 36.97 yrs ± 9.52; 85% | 200; IG: 100; CG: 100 | StudiCare distance-learning | mixed | online and mobile | 7 modules & 1 booster module à 30-90 min & optional mini-modules; 1-2 modules / week | active control | depressive symptoms, stress symptoms, anxiety symptoms, resilience, self-compassion, self-esteem |
| Heckendorf 2022 | Germany^HI^ | general population during COVID; 42.6 yrs ± 14.3; 82.3% | 351; IG: 175; CG: 176 | Get Calm and Move On (GCMO) | CBT | online | 10 modules (30-45 min each) | active and waitlist control | resilience, depressive symptoms, anxiety symptoms, self-efficacy, wellbeing |
| Heckendorf 2019^FU^ | Germany^HI^ | no specific population; 42.4 yrs ± 10.9; 58.8% | 262; IG: 132; CG: 130 | GET.ON (e/mHealth gratitude training) | mixed (positive psychology, CBT) | online and mobile | 5 weekly 45-60-min sessions and daily app use | waitlist | depressive symptoms, anxiety symptoms, resilience, social support, optimism |
| Hersch 2016 | USA^HI^ | nurses; 41 yrs; 87.5% | 104; IG: 52; CG: 52 | BREATHE: stress management for nurses | CBT | online | 3 months access, seven modules, one additional for nursing managers | waitlist | general distress, active coping |
| Hoorelbeke 2015^FU^ | Belgium^HI^ | students; 20.66 yrs ± 2.12; 91.5% | complete cases: 47; IG: 25; CG: 22 | online cognitive control training (CCT) | cognitive control | online | 10 sessions | active control | depressive symptoms, general distress, resilience, positive affect |
| Hoorelbeke 2017^FU^ | Belgium^HI^ | remitted depressed patients; 46.97 yrs ± 11.47; 66.18% | 68; IG: 34; CG: 34 | adaptive version of the paced auditory serial addition task (PASAT) | cognitive control | online | 10 sessions over 2 weeks | active control | depressive symptoms, quality of life, resilience |
| Hsieh 2020 | Taiwan^HI^ | nurses; 33.75 yrs ± 7.05; 77.91% | complete cases: IG: 47; CG: 39 | smartphone biofeedback | biofeedback literature | mobile | 2-hrs resilience course (2 hrs) plus 6 smartphone-delivered BT session | waitlist | depressive symptoms, resilience |
| Jackson 2019 | USA^HI^ | students; 18-22 yrs; 55.4% | Completed baseline: 121; IG: 68; CG: 53 | not specified | CBT | online | 4 weeks with 1 module per week | no intervention | active coping |
| Kahn 2016 | USA^HI^ | military families; 33 yrs ± 6.78; 49.7% | 160 (80 dyads); IG: 80 (40 dyads); CG: 80 (40 dyads) | mission reconnect (MR) | mixed (mindfulness, positive psychology) | online and mobile | 11 individual activities over 8 weeks | waitlist | stress symptoms, depressive symptoms, PTSD symptoms, self-compassion, social support |
| Kim 2018 | Korea^HI^ | employees; 40.29 yrs ± 10.82; 93.05% | 53; IG: 25; CG: 28 | videoconferencing group | mixed (CBT, positive psychology) | mobile | 1 session a week over 4 weeks | active control | stress symptoms, resilience |
| Kloos 2019 | Netherlands^HI^ | nurses; 41.6 yrs ± 12.1; 95% | 165; IG: 88; CG: 77 | this is your life intervention | positive psychology | online | 8 weeks with 1 module per week, 12 weeks access | no intervention | wellbeing |
| Köhle 2021 | Netherlands^HI^ | partners of cancer patients; 55.89 yrs ± 10.72; 70.4% | 203; IG: 137; CG: 66 | hold on for each other | mindfulness | online | 6 modules (2 optional modules, 1-1.5 hrs per week) | waitlist | general distress, mental health, self-compassion, resilience, posttraumatic growth |
| Koydemir 2016 | Turkey^UMI^ | students; 18.75 yrs ± 1.03; 4090% | 92; IG: 48; CG: 44 | first year on campus seminar | positive psychology | online | 5 modules over 8 weeks | care as usual | life satisfaction, happiness, wellbeing, quality of life, mental health |
| Krifa 2021 | Tunisia^LMI^ | healthcare students; 20.74 yrs ± 1.64; 94% | 366; IG: 183; CG: 183 | coherence, attention, relationship, and engagement (CARE) | positive psychology | online | 8 modules (45 min each) | waitlist | stress symptoms, anxiety symptoms, depressive symptoms, optimism, wellbeing |
| Kubo 2019 | USA^HI^ | informal caregivers of cancer patients; 57.6 yrs ± 17.66; 58.06% | 31; IG: 17; CG: 14 | Headspace | mindfulness | mobile | 8-week daily app use | care as usual | stress symptoms, anxiety symptoms, depressive symptoms, quality of life, posttraumatic growth, mindfulness |
| Kuhlthau 2020 | USA^HI^ | parents of children with autism; 45 yrs ± 7.6; 96.1% | 51; IG: 25; CG: 26 | stress management and resiliency training - relaxation response resiliency program (SMART-3RP) | mixed (CBT, mindfulness, positive psychology) | online | 8 weekly sessions with 1.5 h each | waitlist | stress symptoms, resilience, stress coping, depressive symptoms, anxiety symptoms, social support, positive affect, mindfulness |
| Litvin 2020 | UK^HI^ | employees; NR; 38.39% | 491; IG: 222; CG: 269 | eQuoo | mixed (CBT, positive psychology) | mobile | 5 levels over 5 weeks (10-15 min a week) | active control | resilience, wellbeing, anxiety symptoms |
| Liu 2016^FU^ | China^UMI^ | working adults; 34.99 yrs ± 9.37 | 321; IG: 162; CG: 159 | Happy@Work | positive psychology | online | 4 weeks x 1 module per week | waitlist | resilience, depressive symptoms, wellbeing |
| Liu 2018^FU^ | USA^HI^ | students; 20.7 yrs; 77% | 39; IG: 18; CG: 21 | self-affirmation intervention | self-affirmation | online | 5 to 10 minutes over 4 days | active control | (coping) self-efficacy, posttraumatic growth, PTSD symptoms |
| Luo 2021^FU^ | China^UMI^ | parents of children with cancer; 33.6 yrs ± 5.2; 69.9% | 103; IG: 52; CG: 51 | resilience training program | resilience framework | mobile | 8 weeks with 1 tweet / week à 15 min | active control | resilience, depressive symptoms, quality of life |
| Luthans 2008 | USA^HI^ | employees; 32.2 yrs; not reported | 364; IG: 187; CG: 177 | psychological capital intervention | positive psychology | online | 2 sessions with 45 min | active control | resilience |
| Mayor-Silva 2021 | Spain^HI^ | nursing and physical therapy students; 19.7 yrs ± 4.52; 77.1% | 178; IG: 94; CG: 84 | resilience gym | CBT | online | 1 session | no intervention | positive emotions/affect, active coping, resilience |
| Medisauskaite 2019 | UK^HI^ | healthcare providers; not reported; 46.2% | 150; IG: 75; CG: 75 | not specified | job demand models | online | 1 week with 4 modules | no intervention | anxiety symptoms, stress symptoms |
| Mistretta 2018^FU^ | USA^HI^ | healthcare workers; 44.65 yrs ± 13.1; 86.7% | 38; IG: 23; CG: 15 | smartphone resiliency training | not specified | mobile | prompted to select a topic every 7 to 10 days | active control group | depressive symptoms, anxiety symptoms, stress symptoms, wellbeing, self-compassion |
| Mogil 2022^FU^ | USA^HI^ | military-related families with young children; 32.95 yrs ± 5.29; 55.59% | 349; IG: 172; CG: 177 | families overcoming under stress-early childhood (FOCUS-EC) | trauma-informed narrative approach | online | 6 modules typically delivered over 4-10 meetings lasting 60-90 min each | active control | anxiety symptoms, depressive symptoms, PTSD symptoms |
| Mongrain 2016^FU^ | Canada^HI^ | no specific population; 32.64 yrs ± 11.39; 64.5% | 741; IG: 500; CG: 241 | positivity & mindfulness meditation | mixed (positive psychology, mindfulness) | online | daily for 3 weeks | active control | depressive symptoms, life satisfaction, happiness |
| Morledge 2013^FU^ | USA^HI^ | adults; not reported; 88% | 368; IG: 184; CG: 184 | internet-based stress management program plus (ISM+) | mindfulness | online | 8 weeks with weekly sessions | no intervention | mental health, mindfulness, stress symptoms, vitality, wellbeing |
| Mueller 2018 | USA^HI^ | physical therapy students; 26.83 yrs ± 3.31; 25% | 37; IG: 19; CG: 18 | called to care curriculum (CTCC) | positive psychology | online | 11 modules in 10 weeks | waitlist | resilience |
| Mulligan 2022^FU^ | UK^HI^ | parents of children with juvenile idiopathic arthritis; 36.5 yrs ± 6.5; 83.2% | 220; IG: 106; CG: 114 | WebParC intervention | CBT | online and mobile | Unclear (free unlimited access to the website) | care as usual | anxiety symptoms, depressive symptoms |
| Nadler 2020 | USA^HI^ | diverse workplaces; not reported; 73.53% | 275; IG: 138; CG: 137 | online workplace-based mindfulness training | mixed (mindfulness, CBT) | online | 8-week program, 6 out of 7 days a week | waitlist | mindfulness, stress symptoms, resilience, positive emotions/affect |
| Nichols 2015^FU^ | USA^HI^ | spouses of soldiers; 36.55 yrs ± 8.6; 99.34% | 152; IG: 76; CG: 76 | education webinars | CBT | online | 12 30-min sessions | care as usual | depressive symptoms, anxiety symptoms, active coping, resilience, PTSD symptoms |
| Nielsen 2021 | USA^HI^ | legal professionals; 48.61 yrs ± 9.99; 65.63% | 100; IG: 50; CG: 50 | mindful pause | mindfulness | online | 1-hrs webinar with introduction to mindfulness + 30 days of daily emails with guidance for meditation | waitlist | stress symptoms, resilience, depressive symptoms, anxiety symptoms, positive emotions/affect, mindfulness |
| Nixon 2021^FU^ | Germany^HI^ | employees; 42.08 yrs ± 9.16; 77.41% | 270; IG: 135; CG: 135 | adherence-focused group | CBT | online | 8 modules with 45 to 60 min per module | care as usual | stress symptoms, depressive symptoms, resilience |
| Oehler 2019 | Sweden^HI^ | no specific population; 32.52 yrs ± 9.41; 70.54% | 112; IG: 57; CG: 55 | repeated mobile security priming | security priming literature | mobile | daily modules for 7 days (2 min each) | waitlist | Perceived stress, self-compassion, resilience |
| Pandya 2021 | India^LMI^ | mothers of children with autism spectrum disorder; 34.85 yrs ± 2.11; 100% | 137; IG: 79; CG: 58 | WhatsApp-based spiritual posts | transcendence and relational consciousness | mobile | 2 webinars plus 50 weeks of posts | waitlist | resilience |
| Park 2020 | USA^HI^ | parents of young children with learning or attention disabilities; 47 yrs ± 5.7; 90.60% | 54; IG: 31; CG: 23 | stress management and resiliency training-relaxation response resiliency program (SMART-3RP) | mixed (CBT; positive psychology) | online | 9 weeks (kick-off session plus 8 weekly 1.5-hrs intervention sessions) | waitlist | general distress, resilience, active coping, social support, positive emotions/affect, mindfulness |
| Pauls 2016 | Germany^HI^ | employees; 41 yrs ± 11.63; 64% | 113; IG: 57; CG: 56 | web-based mindfulness intervention | mindfulness | online | 4 sessions within 4 workdays (10 min per session) | active control | mindfulness, resilience |
| Pogrebtsova 2018 | Canada^HI^ | undergraduate students enrolled in a psychology class; 18.44 yrs ± 1.36; 79.17% | 89; IG: 46; CG: 43 | mindful reappraisal | mindfulness, emotion regulation | blended | 1-hr in-person introductory session plus 5 days of daily online interventions | active control | positive emotions/affect |
| Profit 2021 | USA^HI^ | healthcare workers; NR; 82.87% | Initiating intervention: 481; IG: 182; CG: 299 | web-based implementation for the science of enhancing resilience (WISER) | positive psychology | online | 6 modules (≤ 10 min) and nightly messages, interaction by site champion | waitlist | depressive symptoms, happiness |
| Proyer 2014^FU^ | Switzerland^HI^ | people aged 50-79 yrs; 55.58 yrs +/- 5.18; 100% | 510; IG: 403; CG: 107 | gratitude visit, three good things, three funny things, signature strengths | positive psychology | online | daily for one week | active control | depressive symptoms, happiness |
| Puertas-Gonzalez 2022 | Spain^HI^ | pregnant women; 35.01 yrs ± 4.02; 100% | 139; IG: 70; CG: 69 | online cognitive behavioral therapy (o-CBT) | CBT | online | 8 weekly 1.5-2-hrs sessions | active control | stress symptoms, resilience, general distress |
| Sutarto 2012 | Malaysia^UMI^ | operators from an electronic manufacturing factory; 36.3 yrs ± 10.14; 100% | 40; IG: 20; CG: 20 | resonant breathing biofeedback training | heartrate variability biofeedback | computer-based | 5 30-50 min sessions, daily practice at home | no intervention | depressive symptoms, anxiety symptoms, stress symptoms |
| Pyne 2019^FU^ | USA^HI^ | Soldiers; 28.7 yrs ± 8.4; 8.2% | 342; IG: 200; CG: 142 | heartrate variability biofeedback plus cognitive bias modification | heartrate variability biofeedback & cognitive bias modification | computer-based and mobile | 1 session plus 1 hrs practice | no intervention | PTSD symptoms |
| Rackoff 2022^FU^ | USA^HI^ | students with elevated distress; 20.44 yrs ± 4.51; 74.02% | 585; IG: 301; CG: 284 | space for resilience & space from COVID-19 | mixed (CBT, positive psychology) | online | 12 modules to choose from at own pace | care as usual | depressive symptoms, anxiety symptoms, stress symptoms |
| Ramey 2017 | USA^HI^ | police officers; 25.7 yrs ± 5.9; 18% | 36; IG: 18; CG: 18 | resilience training program | heartrate variability biofeedback | online and mobile | 2-hr educational session, 4 1-hr mentoring sessions plus home practice | active control | stress symptoms, resilience, PTSD symptoms, general distress, vitality |
| Riello 2021^FU^ | Italy^HI^ | workers of residential nursing and care homes; NR; 88.24% | 238; IG: 119; CG: 119 | self-help plus (SH+) - version: Doing what matters in times of stress | mindfulness | mobile | 5 weeks with access to materials for the five core components of SH+ (1 component per week) | active control | anxiety symptoms, PTSD symptoms, quality of life, resilience, stress symptoms |
| Roepke 2018^FU^ | USA^HI^ | adults after adversity; 43.54 yrs ± 12.86; 88.79% | 116; IG: 61; CG: 55 | prospective writing | expressive writing | online | 4 weeks with 4 15-minute writing sessions | active control | depressive symptoms, PTSD symptoms, posttraumatic growth, life satisfaction |
| Röhr 2021^FU^ | Germany^HI^ | Syrian refugees living in Germany; 33.33 yrs ± 11.2; 38.3% | 133; IG: 65; CG: 68 | Sanadak | CBT | mobile | NR | active control | PTSD symptoms, depressive symptoms, anxiety symptoms, quality of life, self-efficacy, social support, posttraumatic growth |
| Roig 2020 | Ireland^HI^ | students; 26 yrs, IQR: 11; 83% | 83; IG: 55; CG: 28 | space for resilience (with human or automated support) | resilience factor literature | online | 8-week period with 7 modules | waitlist | Resilience (2 measures, CD-RISC & BRS), happiness, depressive symptoms, self-esteem, stress |
| Rose 2013 | USA^HI^ | students; 27.32 yrs ± 3.53; 50% | 66; IG: 34; CG: 32 | SMART-OP | CBT | computer-based and online | 6 weeks with 6 modules, between session homework | active control | active coping, stress symptoms |
| Rullo 2021 | USA^HI^ | couples (of females with sexual dysfunction, only partners relevant); 44.1 yrs ± 10.4; 0% | 60 couples; IG: 30 couples; CG: 30 couples | sex stress management and resiliency training (SMART) | mindfulness | online | 12 videos (120 min), 7 30-min sessions on sexual health | care as usual | anxiety symptoms, resilience, stress symptoms, happiness, life satisfaction |
| Schotanus-Dijkstra 2019^FU^ | Netherlands^HI^ | general population; 48 yrs ± 10.9; 85.8% | 275; IG: 137; CG: 138 | this is your life (guided self-help version) | positive psychology | online | 8 modules over 12 weeks | waitlist | wellbeing, depressive symptoms, anxiety symptoms, positive emotions/affect, optimism, self-compassion, resilience, positive relations |
| Seear 2013^FU^ | Australia^HI^ | general population; 34 yrs ± 13.97; 75.36% | 211; IG: 144; CG: 67 | three good things, best possible self | mixed (positive psychology, mindfulness) | online | 1 week with 1 exercise per day | no intervention | wellbeing, positive emotions/affect |
| Seligman 2007 | USA^HI^ | students; NR; 65% | 240; IG: 113; CG: 127 | workshop-group | CBT | blended | 8 weeks with 1 2-hrs session per week plus web-based support and coaching via e-mail for up to 3 yrs | no intervention | depressive symptoms, anxiety symptoms, wellbeing |
| Sim 2020^FU^ | Australia^HI^ | parents and their children (8-11 yrs); 41.34 yrs ± 5.22; 89.9% | 355; IG: 177; CG: 178 | web-based parenting resilient kids (PARK) program | parenting strategies program | online | up to 12 modules over 12 weeks | active control | quality of life |
| Spilg 2022^FU^ | Canada^HI^ | physicians in a tertiary care academic hospital; 45.9 yrs ± 9.49; 35% | 40; IG: 20; CG: 20 | stress management and resilience training (SMART) | attention and interpretation therapy | online | 24 weeks | no intervention | resilience, happiness, stress symptoms |
| Stephens 2012^FU^ | USA^HI^ | adolescent nursing students; 20.9 yrs ± 0.95; 88.6% | 70; IG: 35; CG: 35 | not specified | Ahern's model of adolescent resilience | online | 4 educational messages and/or questions (tweets) for 6 weeks | active control | stress symptoms, social support, resilience |
| Stockton 2014^FU^ | UK^HI^ | adults after traumatic events; 33.18 yrs ± 12.31; 95.83% | 53; IG: 18; CG: 35 | expressive writing | expressive writing literature | online | 1 week of writing exercises on 3 days | active control | PTSD symptoms, posttraumatic growth, wellbeing |
| Tagalidou 2019^FU^ | Austria^HI^ | students (mostly); 24.91 yrs ± 8.22; 85.2% | 182; IG: 133; CG: 49 | coping humor, three funny things, three good things | literature on humorous coping | online | 8 days with 7 days of diary writing in the evening | active control | depressive symptoms |
| van Berkel 2014^FU^ | Netherlands^HI^ | employees of research institutes; 45.55 yrs ± 9.49; 67.32% | 257; IG: 129; CG: 128 | mindfulness VIP group | mindfulness | blended | 8 weeks of 90-min in-person training plus 8 sessions of e-coaching | care as usual | general distress, mindfulness |
| Van der Houwen 2010^FU^ | USA/UK^HI^ | bereaved individuals; 43.22 yrs ± 10.98; 93.50% | 757; IG: 460; CG: 297 | confrontational writing intervention | literature on confrontational writing | online | 5 structured writing assignments (over 7 weeks) | waitlist | social support, depressive symptoms, positive emotions/affect |
| van der Meer 2020^FU^ | Netherlands^HI^ | healthcare professionals; 43.37 yrs ± 9.72; 52.9% | 287; IG: 143; CG: 144 | SUPPORT Coach | CBT | mobile | 1 month of free app usage | no intervention | PTSD symptoms, resilience, social support |
| Villani 2013 | Italy^HI^ | Oncology nurses; 43 yrs ± 8.8; 100% | 30; IG: 15; CG: 15 | mobile stress inoculation training (M-SIT) | CBT | mobile | 8 15-min sessions in 4 weeks | active control | anxiety symptoms, active coping |
| Wijesekera 2019 | USA^HI^ | Caregivers (families) of pediatric heart transplant patients; 82.35% | 17; IG: 9; CG: 8 | families overcoming under stress – pediatric heart transplant (FOCUS-PedsHT) | not specified | online | 8-10 video sessions | care as usual | PTSD symptoms, depressive symptoms, anxiety symptoms |
| Yousefi 2022^FU^ | Iran^LMI^ | cancer patients' caregivers; 44.27 yrs ± 6.28; 66.04% | 53; IG: 28; CG: 25 | online mindfulness-based cancer recovery (E-MBCR) | mindfulness | online | 9 weeks with 1 60-90-min module per week | no intervention | resilience, wellbeing |
| Zahedifar 2021 | Iran^LMI^ | people referring to a healthcare center; 43 yrs ± 10.2; 65.8% | 480; IG: 240; CG: 240 | educational intervention | not specified | online | 5 weeks with 1 120-min group session | no intervention | anxiety symptoms, quality of life |

*Note*. Rating of income group according to the World Bank Atlas method^68^.

FU = follow-up data available; HI = high-income country; LMI = lower middle-income country; UMI = upper middle-income country; NR = not reported.

^a^ Please note that our literature search ended in August 2022. However, in this search, we identified records (e.g., study protocols, registrations) that were potentially eligible, but results had not yet been published. Thus, for those records we also accepted later publications.

### Supplementary Data 4. Risk of bias ratings

**Table 3** Risk of bias for included effect estimates based on the Cochrane risk-of-bias tool for randomized trials (RoB2)

| \|  \|  \| **Domains** \| \| \| \| \| \| \| --- \| --- \| --- \| --- \| --- \| --- \| --- \| --- \| \| **Study ID** \| **Outcome type** \| **Randomization process** \| **Deviations from intended interventions** \| **Missing outcome data** \| **Measurement of outcome** \| **Selection of reported result** \| **Overall rating** \| \| Abbott 2009 \| depressive symptoms \| High \| Low \| High \| High \| Some concerns \| High \| \| Abbott 2009 \| anxiety symptomsd \| High \| Low \| High \| High \| Some concerns \| High \| \| Abbott 2009 \| stress symptoms \| High \| Low \| High \| High \| Some concerns \| High \| \| Abbott 2009 \| quality of life \| High \| Low \| High \| High \| Some concerns \| High \| \| Aboody 2020 \| depressive symptoms \| High \| Low \| Low \| High \| Low \| High \| \| Aboody 2020 \| self-esteem \| High \| Low \| Low \| High \| Low \| High \| \| Aikens 2014 \| mindfulness \| Some concerns \| Low \| High \| High \| Some concerns \| High \| \| Aikens 2014 \| resilience \| Some concerns \| Low \| High \| High \| Some concerns \| High \| \| Aikens 2014 \| vitality \| Some concerns \| Low \| High \| High \| Some concerns \| High \| \| Al-Refae 2021 \| depressive symptoms \| High \| Low \| Low \| High \| Some concerns \| High \| \| Al-Refae 2021 \| anxiety symptoms \| High \| Low \| Low \| High \| Some concerns \| High \| \| Al-Refae 2021 \| stress symptoms \| High \| Low \| Low \| High \| Some concerns \| High \| \| Al-Refae 2021 \| self-compassion \| High \| Low \| Low \| High \| Some concerns \| High \| \| Al-Refae 2021 \| mindfulness \| High \| Low \| Low \| High \| Some concerns \| High \| \| Aminoff 2021 \| depressive symptoms \| Low \| Low \| High \| High \| Low \| High \| \| Aminoff 2021 \| quality of life \| Low \| Low \| High \| High \| Low \| High \| \| Aminoff 2021 \| depressive symptoms \| Low \| Low \| High \| High \| Low \| High \| \| Aminoff 2021 \| anxiety symptoms \| Low \| Low \| High \| High \| Low \| High \| \| Aminoff 2021 \| PTSD symptoms \| Low \| Low \| High \| High \| Low \| High \| \| Aminoff 2021 \| stress symptoms \| Low \| Low \| High \| High \| Low \| High \| \| Atad 2021 \| resilience \| Low \| Low \| High \| Low \| Some concerns \| High \| \| Atad 2021 \| general distress \| Low \| Low \| High \| Low \| Some concerns \| High \| \| Auyeung 2019 \| depressive symptoms \| Low \| Low \| Low \| Low \| Some concerns \| Some concerns \| \| Auyeung 2019 \| positive emotions/affect \| Low \| Low \| Low \| Low \| Some concerns \| Some concerns \| \| Ayers 2015 \| positive emotions/affect \| Low \| Low \| Low \| Low \| Some concerns \| Some concerns \| \| Bani Ahmad 2021 \| stress symptoms \| Some concerns \| Low \| High \| High \| Some concerns \| High \| \| Bani Ahmad 2021 \| active coping \| Some concerns \| Low \| High \| High \| Some concerns \| High \| \| Ben-Avraham 2022 \| resilience \| Low \| Some concerns \| Low \| Low \| Some concerns \| Some concerns \| \| Ben-Avraham 2022 \| self-efficacy \| Low \| Some concerns \| Low \| Low \| Some concerns \| Some concerns \| \| Ben-Avraham 2022 \| quality of life \| Low \| Some concerns \| Low \| Low \| Some concerns \| Some concerns \| \| Ben-Avraham 2022 \| general distress \| Low \| Some concerns \| Low \| Low \| Some concerns \| Some concerns \| \| Ben-Avraham 2022 \| anxiety symptoms \| Low \| Some concerns \| Low \| Low \| Some concerns \| Some concerns \| \| Beyer 2010 \| general distress \| Low \| Some concerns \| Low \| Low \| Some concerns \| Some concerns \| \| Beyer 2010 \| anxiety symptoms \| Low \| Some concerns \| Low \| Low \| Some concerns \| Some concerns \| \| Beyer 2010 \| depressive symptoms \| Low \| Some concerns \| Low \| Low \| Some concerns \| Some concerns \| \| Beyer 2010 \| PTSD symptoms \| Low \| Some concerns \| Low \| Low \| Some concerns \| Some concerns \| \| Beyer 2010 \| posttraumatic growth \| Low \| Some concerns \| Low \| Low \| Some concerns \| Some concerns \| \| Birk 2019 \| anxiety symptoms \| Some concerns \| Low \| High \| Low \| Some concerns \| High \| \| Bolier 2014 \| mental health \| Some concerns \| Low \| High \| High \| Low \| High \| \| Bolier 2014 \| wellbeing \| Some concerns \| Low \| High \| High \| Low \| High \| \| Bolier 2014 \| depression symptoms \| Some concerns \| Low \| High \| High \| Low \| High \| \| Bolier 2014 \| anxiety symptoms \| Some concerns \| Low \| High \| High \| Low \| High \| \| Bouchard 2012 \| sel-efficacy \| Some concerns \| Low \| High \| Some concerns \| Some concerns \| High \| \| Brog 2021 \| depressive symptoms \| Some concerns \| Some concerns \| Low \| High \| Low \| High \| \| Brog 2021 \| general distress \| Some concerns \| Some concerns \| Low \| High \| Low \| High \| \| Brog 2021 \| quality of life \| Some concerns \| Some concerns \| Low \| High \| Low \| High \| \| Brog 2021 \| optimism \| Some concerns \| Some concerns \| Low \| High \| Low \| High \| \| Brog 2021 \| self-efficacy \| Some concerns \| Some concerns \| Low \| High \| Low \| High \| \| Brog 2021 \| resilience \| Some concerns \| Some concerns \| Low \| High \| Low \| High \| \| Burger 2015 \| stress symptoms \| High \| Low \| Low \| High \| Some concerns \| High \| \| Burger 2015 \| mindfulness \| High \| Low \| Low \| High \| Some concerns \| High \| \| Catuara-Solarz 2022 \| anxiety symptoms \| Low \| Low \| High \| High \| Some concerns \| High \| \| Catuara-Solarz 2022 \| life satisfaction \| Low \| Low \| High \| High \| Some concerns \| High \| \| Catuara-Solarz 2022 \| anxiety symptoms \| Low \| Low \| High \| High \| Some concerns \| High \| \| Catuara-Solarz 2022 \| wellbeing \| Low \| Low \| High \| High \| Some concerns \| High \| \| Catuara-Solarz 2022 \| resilience \| Low \| Low \| High \| High \| Some concerns \| High \| \| Catuara-Solarz 2022 \| stress symptoms \| Low \| Low \| High \| High \| Some concerns \| High \| \| Cavanagh 2018 \| stress symptoms \| Low \| Low \| High \| High \| Some concerns \| High \| \| Cavanagh 2018 \| mindfulness \| Low \| Low \| High \| High \| Some concerns \| High \| \| Cavanagh 2018 \| general distress \| Low \| Low \| High \| High \| Some concerns \| High \| \| Champion 2018 \| life satisfaction \| High \| Low \| High \| High \| Low \| High \| \| Champion 2018 \| stress symptoms \| High \| Low \| High \| High \| Low \| High \| \| Champion 2018 \| resilience \| High \| Low \| High \| High \| Low \| High \| \| Chang 2022 \| stress symptoms \| Low \| Low \| High \| High \| Low \| High \| \| Chang 2022 \| wellbeing \| Low \| Low \| High \| High \| Low \| High \| \| Chang 2022 \| positive emotions/affect \| Low \| Low \| High \| High \| Low \| High \| \| Chang 2022 \| anxiety symptoms \| Low \| Low \| High \| High \| Low \| High \| \| Chang 2022 \| depressive symptoms \| Low \| Low \| High \| High \| Low \| High \| \| Chang 2022 \| resilience \| Low \| Low \| High \| High \| Low \| High \| \| Chilver 2021 \| wellbeing \| Low \| Some concerns \| High \| Low \| Some concerns \| High \| \| Chilver 2021 \| resilience \| Low \| Some concerns \| High \| Low \| Some concerns \| High \| \| Chilver 2021 \| self-compassion \| Low \| Some concerns \| High \| Low \| Some concerns \| High \| \| Chilver 2021 \| life satisfaction \| Low \| Some concerns \| High \| Low \| Some concerns \| High \| \| Chilver 2021 \| depressiv symptoms \| Low \| Some concerns \| High \| Low \| Some concerns \| High \| \| Chilver 2021 \| anxiety symptoms \| Low \| Some concerns \| High \| Low \| Some concerns \| High \| \| Chilver 2021 \| stress symptoms \| Low \| Some concerns \| High \| Low \| Some concerns \| High \| \| Cieslak 2016 \| self-efficacy \| Some concerns \| Low \| Low \| Some concerns \| Some concerns \| Some concerns \| \| Cieslak 2016 \| PTSD symptoms (secondary) \| Some concerns \| Low \| Low \| Some concerns \| Some concerns \| Some concerns \| \| Cieslak 2016 \| posttraumatic growth \| Some concerns \| Low \| Low \| Some concerns \| Some concerns \| Some concerns \| \| Decker 2020 \| resilience \| Low \| Low \| Low \| Some concerns \| Some concerns \| High \| \| Decker 2020 \| depressive symptoms \| Low \| Low \| Low \| Some concerns \| Some concerns \| High \| \| Decker 2020 \| self-efficacy \| Low \| Low \| Low \| Some concerns \| Some concerns \| High \| \| Ebert 2021 \| stress symptoms \| Low \| Low \| Low \| High \| Some concerns \| High \| \| Ebert 2021 \| depressive symptoms \| Low \| Low \| Low \| High \| Some concerns \| High \| \| Ebert 2021 \| resilience \| Low \| Low \| Low \| High \| Some concerns \| High \| \| Ebert 2021 \| wellbeing \| Low \| Low \| Low \| High \| Some concerns \| High \| \| Enrique 2018 \| positive emotions/affect \| Low \| Low \| Low \| Low \| Some concerns \| Some concerns \| \| Enrique 2018 \| optimism \| Low \| Low \| Low \| Low \| Some concerns \| Some concerns \| \| Enrique 2018 \| depressive symptoms \| Low \| Low \| Low \| Low \| Some concerns \| Some concerns \| \| Eriksson 2018 \| self-compassion \| High \| Some concerns \| High \| High \| Some concerns \| High \| \| Eriksson 2018 \| mindfulness \| High \| Some concerns \| High \| High \| Some concerns \| High \| \| Eriksson 2018 \| stress symptoms \| High \| Some concerns \| High \| High \| Some concerns \| High \| \| Fassnacht 2022 \| wellbeing \| Some concerns \| Some concerns \| Low \| High \| Low \| High \| \| Fassnacht 2022 \| resilience \| Some concerns \| Some concerns \| Low \| High \| Low \| High \| \| Fassnacht 2022 \| anxiety symptoms \| Some concerns \| Some concerns \| Low \| High \| Low \| High \| \| Fassnacht 2022 \| depressive symptoms \| Some concerns \| Some concerns \| Low \| High \| Low \| High \| \| Fassnacht 2022 \| self-efficacy \| Some concerns \| Some concerns \| Low \| High \| Low \| High \| \| Feinberg 2020 \| depressive symptoms \| Some concerns \| Low \| High \| High \| Some concerns \| High \| \| Flett 2020 \| stress symptoms \| High \| Low \| HIgh \| High \| Low \| High \| \| Flett 2020 \| resilience \| High \| Low \| HIgh \| High \| Low \| High \| \| Flett 2020 \| self-efficacy \| High \| Low \| HIgh \| High \| Low \| High \| \| Flett 2020 \| mindfulness \| High \| Low \| HIgh \| High \| Low \| High \| \| Flett 2019 \| depressive symptoms \| High \| Low \| Low \| Low \| Low \| High \| \| Flett 2019 \| anxiety symptoms \| High \| Low \| Low \| Low \| Low \| High \| \| Flett 2019 \| stress symptoms \| High \| Low \| Low \| Low \| Low \| High \| \| Flett 2019 \| resilience \| High \| Low \| Low \| Low \| Low \| High \| \| Flett 2019 \| mindfulness \| High \| Low \| Low \| Low \| Low \| High \| \| Gollwitzer 2018 \| stress symptoms \| Some concerns \| Some concerns \| Low \| Some concerns \| Some concerns \| High \| \| Grabbe 2020 \| wellbeing \| Some concerns \| Some concerns \| High \| Low \| Some concerns \| High \| \| Grabbe 2020 \| resilience \| Some concerns \| Some concerns \| High \| Low \| Some concerns \| High \| \| Grabbe 2020 \| PTSD symptoms (secondary) \| Some concerns \| Some concerns \| High \| Low \| Some concerns \| High \| \| Hannibal 2022 \| mindfulness \| Some concerns \| Some concerns \| Unclear \| Unclear \| Unclear \| Unclear \| \| Hannibal 2022 \| stress symptoms \| Some concerns \| Some concerns \| Unclear \| Some concerns \| Unclear \| Unclear \| \| Hannibal 2022 \| resilience \| Some concerns \| Some concerns \| Unclear \| Some concerns \| Unclear \| Unclear \| \| Hannibal 2022 \| self-efficacy \| Some concerns \| Some concerns \| Unclear \| Some concerns \| Unclear \| Unclear \| \| Hannibal 2022 \| optimism \| Some concerns \| Some concerns \| Unclear \| Some concerns \| Unclear \| Unclear \| \| Hannibal 2022 \| social support \| Some concerns \| Some concerns \| Unclear \| Some concerns \| Unclear \| Unclear \| \| Hannibal 2022 \| depressive symptoms \| Some concerns \| Some concerns \| Unclear \| Some concerns \| Unclear \| Unclear \| \| Harrer 2018 \| stress symptoms \| Low \| Low \| High \| High \| Low \| High \| \| Harrer 2018 \| depressive symptoms \| Low \| Low \| High \| High \| Low \| High \| \| Harrer 2018 \| anxiety symptoms \| Low \| Low \| High \| High \| Low \| High \| \| Harrer 2018 \| wellbeing \| Low \| Low \| High \| High \| Low \| High \| \| Harrer 2018 \| resilience \| Low \| Low \| High \| High \| Low \| High \| \| Harrer 2018 \| self-compassion \| Low \| Low \| High \| High \| Low \| High \| \| Harrer 2018 \| self-esteem \| Low \| Low \| High \| High \| Low \| High \| \| Harrer 2021 \| depressive symptoms \| Low \| Low \| Some concerns \| Some concerns \| Low \| Some concerns \| \| Harrer 2021 \| stress symptoms \| Low \| Low \| Some concerns \| Some concerns \| Low \| Some concerns \| \| Harrer 2021 \| anxiety symptoms \| Low \| Low \| Some concerns \| Some concerns \| Low \| Some concerns \| \| Harrer 2021 \| resilience \| Low \| Low \| Some concerns \| Some concerns \| Low \| Some concerns \| \| Harrer 2021 \| self-compassion \| Low \| Low \| Some concerns \| Some concerns \| Low \| Some concerns \| \| Harrer 2021 \| self-esteem \| Low \| Low \| Some concerns \| Some concerns \| Low \| Some concerns \| \| Heckendorf 2022 \| depressive symptoms \| Low \| Low \| Low \| Some concerns \| Low \| High \| \| Heckendorf 2022 \| anxiety symptoms \| Low \| Low \| Low \| Some concerns \| Low \| High \| \| Heckendorf 2022 \| resilience \| Low \| Low \| Low \| Some concerns \| Low \| High \| \| Heckendorf 2022 \| self-efficacy \| Low \| Low \| Low \| Some concerns \| Low \| High \| \| Heckendorf 2022 \| wellbeing \| Low \| Low \| Low \| Some concerns \| Low \| High \| \| Heckendorf 2019 \| depressive symptoms \| Low \| Low \| Some concerns \| High \| Low \| High \| \| Heckendorf 2019 \| anxiety symptoms \| Low \| Low \| Some concerns \| High \| Low \| High \| \| Heckendorf 2019 \| resilience \| Low \| Low \| Some concerns \| High \| Low \| High \| \| Heckendorf 2019 \| social support \| Low \| Low \| Some concerns \| High \| Low \| High \| \| Heckendorf 2019 \| optimism \| Low \| Low \| Some concerns \| High \| Low \| High \| \| Hersch 2016 \| general distress \| Some concerns \| Low \| Some concerns \| High \| Some concerns \| High \| \| Hersch 2016 \| active coping \| Some concerns \| Low \| Some concerns \| High \| Some concerns \| High \| \| Hoorelbeke 2015 \| depressive symptoms \| Some concerns \| Low \| Low \| Low \| Some concerns \| Some concerns \| \| Hoorelbeke 2015 \| general distress \| Some concerns \| Low \| Low \| Low \| Some concerns \| Some concerns \| \| Hoorelbeke 2015 \| resilience \| Some concerns \| Low \| Low \| Low \| Some concerns \| Some concerns \| \| Hoorelbeke 2015 \| positive emotions/affect \| Some concerns \| Low \| Low \| Low \| Some concerns \| Some concerns \| \| Hoorelbeke 2017 \| depressive symptoms \| Low \| Low \| Low \| Low \| Low \| Low \| \| Hoorelbeke 2017 \| quality of life \| Low \| Low \| Low \| Low \| Low \| Low \| \| Hoorelbeke 2017 \| resilience \| Low \| Low \| Low \| Low \| Low \| Low \| \| Hsieh 2020 \| depressive symptoms \| High \| Low \| High \| High \| Some concerns \| High \| \| Hsieh 2020 \| resilience \| High \| Low \| High \| High \| Some concerns \| High \| \| Kahn 2016 \| stress symptoms \| High \| Some concerns \| Low \| High \| Low \| High \| \| Kahn 2016 \| depressive symptoms \| High \| Some concerns \| Low \| High \| Low \| High \| \| Kahn 2016 \| PTSD symptoms \| High \| Some concerns \| Low \| High \| Low \| High \| \| Kahn 2016 \| self-compassion \| High \| Some concerns \| Low \| High \| Low \| High \| \| Kahn 2016 \| social support \| High \| Some concerns \| Low \| High \| Low \| High \| \| Kim 2018 \| stress symptoms \| Some concerns \| Low \| Low \| Some concerns \| Low \| Some concerns \| \| Kim 2018 \| resilience \| Some concerns \| Low \| Low \| Some concerns \| Low \| Some concerns \| \| Kloos 2019 \| wellbeing \| High \| Low \| High \| High \| Some concerns \| High \| \| Köhle 2021 \| general distress \| Some concerns \| Low \| Low \| High \| Low \| High \| \| Köhle 2021 \| mental health \| Some concerns \| Low \| Low \| High \| Low \| High \| \| Köhle 2021 \| self-compassion \| Some concerns \| Low \| Low \| High \| Low \| High \| \| Köhle 2021 \| resilience \| Some concerns \| Low \| Low \| High \| Low \| High \| \| Köhle 2021 \| posttraumatic growth \| Some concerns \| Low \| Low \| High \| Low \| High \| \| Koydemir 2016 \| life satisfaction \| Some concerns \| Low \| High \| High \| Some concerns \| High \| \| Koydemir 2016 \| happiness \| Some concerns \| Low \| High \| High \| Some concerns \| High \| \| Koydemir 2016 \| wellbeing \| Some concerns \| Low \| High \| High \| Some concerns \| High \| \| Koydemir 2016 \| social support (relationships) \| Some concerns \| Low \| High \| High \| Some concerns \| High \| \| Koydemir 2016 \| mental health \| Some concerns \| Low \| High \| High \| Some concerns \| High \| \| Krifa 2021 \| stress symptoms \| Low \| Low \| Low \| High \| Some concerns \| High \| \| Krifa 2021 \| depressive symptoms \| Low \| Low \| Low \| High \| Some concerns \| High \| \| Krifa 2021 \| anxiety symptoms \| Low \| Low \| Low \| High \| Some concerns \| High \| \| Krifa 2021 \| optimism \| Low \| Low \| Low \| High \| Some concerns \| High \| \| Krifa 2021 \| wellbeing \| Low \| Low \| Low \| High \| Some concerns \| High \| \| Kubo 2019 \| general distress \| High \| Low \| High \| High \| Low \| High \| \| Kubo 2019 \| anxiety symptoms \| High \| Low \| High \| High \| Low \| High \| \| Kubo 2019 \| depressive symptoms \| High \| Low \| High \| High \| Low \| High \| \| Kubo 2019 \| quality of life \| High \| Low \| High \| High \| Low \| High \| \| Kubo 2019 \| posttraumatic growth \| High \| Low \| High \| High \| Low \| High \| \| Kubo 2019 \| mindfulness \| High \| Low \| High \| High \| Low \| High \| \| Kuhlthau 2020 \| general distress \| Some concerns \| Low \| Low \| High \| Some concerns \| High \| \| Kuhlthau 2020 \| resilience \| Some concerns \| Low \| Low \| High \| Some concerns \| High \| \| Kuhlthau 2020 \| active coping \| Some concerns \| Low \| Low \| High \| Some concerns \| High \| \| Kuhlthau 2020 \| social support \| Some concerns \| Low \| Low \| High \| Some concerns \| High \| \| Kuhlthau 2020 \| positive emotions/affect \| Some concerns \| Low \| Low \| High \| Some concerns \| High \| \| Kuhlthau 2020 \| mindfulness \| Some concerns \| Low \| Low \| High \| Some concerns \| High \| \| Litvin 2020 \| resilience \| Low \| Low \| High \| Low \| Low \| High \| \| Litvin 2020 \| wellbeing \| Low \| Low \| High \| Low \| Low \| High \| \| Litvin 2020 \| anxiety symptoms \| Low \| Low \| High \| Low \| Low \| High \| \| Liu 2016 \| psychological capital \| Low \| Low \| High \| High \| Low \| High \| \| Liu 2016 \| depressive symptoms \| Low \| Low \| High \| High \| Low \| High \| \| Liu 2016 \| wellbeing \| Low \| Low \| High \| High \| Low \| High \| \| Liu 2016 \| wellbeing \| Low \| Low \| High \| High \| Low \| High \| \| Liu 2018 \| self-efficacy \| Some concerns \| Low \| Low \| Low \| Some concerns \| Some concerns \| \| Liu 2018 \| posttraumatic growth \| Some concerns \| Low \| Low \| Low \| Some concerns \| Some concerns \| \| Liu 2018 \| PTSD symptoms \| Some concerns \| Low \| Low \| Low \| Some concerns \| Some concerns \| \| Luo 2021 \| resilience \| Low \| Low \| Low \| Some concerns \| Low \| Some concerns \| \| Luo 2021 \| depressive symptoms \| Low \| Low \| Low \| Some concerns \| Low \| Some concerns \| \| Luo 2021 \| quality of life \| Low \| Low \| Low \| Some concerns \| Low \| Some concerns \| \| Luthans 2008 \| resilience \| Some concerns \| Low \| High \| Low \| Some concerns \| High \| \| Mayor-Silva 2021 \| resilience \| Some concerns \| Low \| Low \| High \| Some concerns \| High \| \| Mayor-Silva 2021 \| active coping \| Some concerns \| Low \| Low \| High \| Some concerns \| High \| \| Mayor-Silva 2021 \| positive emotions/affect \| Some concerns \| Low \| Low \| High \| Some concerns \| High \| \| Medisauskaite 2019 \| anxiety symptoms \| Low \| Low \| Low \| High \| Low \| High \| \| Medisauskaite 2019 \| general distress \| Low \| Low \| Low \| High \| Low \| High \| \| Mistretta 2018 \| stress symptoms \| High \| Low \| Some concerns \| Some concerns \| Low \| High \| \| Mistretta 2018 \| depressive symptoms \| High \| Low \| Some concerns \| Some concerns \| Low \| High \| \| Mistretta 2018 \| anxiety symptoms \| High \| Low \| Some concerns \| Some concerns \| Low \| High \| \| Mistretta 2018 \| wellbeing \| High \| Low \| Some concerns \| Some concerns \| Low \| High \| \| Mistretta 2018 \| self-compassion \| High \| Low \| Some concerns \| Some concerns \| Low \| High \| \| Mogil 2022 \| anxiety symptoms \| High \| Some concerns \| Low \| Some concerns \| Some concerns \| High \| \| Mogil 2022 \| depressive symptoms \| High \| Some concerns \| Low \| Some concerns \| Some concerns \| High \| \| Mogil 2022 \| PTSD symptoms \| High \| Some concerns \| Low \| Some concerns \| Some concerns \| High \| \| Mongrain 2016 \| depressive symptoms \| High \| Low \| High \| Low \| Some concerns \| High \| \| Mongrain 2016 \| life satisfaction \| High \| Low \| High \| Low \| Some concerns \| High \| \| Mongrain 2016 \| happiness \| High \| Low \| High \| Low \| Some concerns \| High \| \| Morledge 2013 \| stress symptoms \| Low \| High \| High \| High \| Some concerns \| High \| \| Morledge 2013 \| mindfulness \| Low \| High \| High \| High \| Some concerns \| High \| \| Morledge 2013 \| wellbeing \| Low \| High \| High \| High \| Some concerns \| High \| \| Morledge 2013 \| vitality \| Low \| High \| High \| High \| Some concerns \| High \| \| Morledge 2013 \| mental health \| Low \| High \| High \| High \| Some concerns \| High \| \| Mueller 2018 \| resilience \| Low \| Low \| Low \| High \| Some concerns \| High \| \| Mulligan 2022 \| anxiety symptoms \| Low \| Low \| Low \| High \| Low \| High \| \| Mulligan 2022 \| depressive symptoms \| Low \| Low \| Low \| High \| Low \| High \| \| Nadler 2020 \| mindfulness \| Some concerns \| Low \| High \| High \| Some concerns \| High \| \| Nadler 2020 \| stress symptoms \| Some concerns \| Low \| High \| High \| Some concerns \| High \| \| Nadler 2020 \| resilience \| Some concerns \| Low \| High \| High \| Some concerns \| High \| \| Nadler 2020 \| positive emotions/affect \| Some concerns \| Low \| High \| High \| Some concerns \| High \| \| Nichols 2015 \| anxiety symptoms \| Some concerns \| Low \| High \| High \| Low \| High \| \| Nichols 2015 \| depressive symptoms \| Some concerns \| Low \| High \| High \| Low \| High \| \| Nichols 2015 \| resilience \| Some concerns \| Low \| High \| High \| Low \| High \| \| Nichols 2015 \| active coping \| Some concerns \| Low \| High \| High \| Low \| High \| \| Nielsen 2021 \| stress symptoms \| Some concerns \| Low \| High \| High \| Low \| High \| \| Nielsen 2021 \| positive emotions/ affect \| Some concerns \| Low \| High \| High \| Low \| High \| \| Nielsen 2021 \| resilience \| Some concerns \| Low \| High \| High \| Low \| High \| \| Nielsen 2021 \| mindfulness \| Some concerns \| Low \| High \| High \| Low \| High \| \| Nielsen 2021 \| depressive symptoms \| Some concerns \| Low \| High \| High \| Low \| High \| \| Nielsen 2021 \| anxiety symptoms \| Some concerns \| Low \| High \| High \| Low \| High \| \| Nielsen 2021 \| stress symptoms \| Some concerns \| Low \| High \| High \| Low \| High \| \| Nixon 2021 \| depressive symptoms \| Some concerns \| Low \| Low \| High \| Low \| High \| \| Nixon 2021 \| stress symptoms \| Some concerns \| Low \| Low \| High \| Low \| High \| \| Nixon 2021 \| resilience \| Some concerns \| Low \| Low \| High \| Low \| High \| \| Oehler 2019 \| stress symptoms \| Some concerns \| Low \| Low \| High \| Some concerns \| High \| \| Oehler 2019 \| self-compassion \| Some concerns \| Low \| Low \| High \| Some concerns \| High \| \| Oehler 2019 \| resilience \| Some concerns \| Low \| Low \| High \| Some concerns \| High \| \| Pandya 2021 \| resilience \| Some concerns \| Low \| Low \| High \| Some concerns \| High \| \| Park 2020 \| resilience \| Some concerns \| Low \| High \| High \| Some concerns \| High \| \| Park 2020 \| active coping \| Some concerns \| Low \| High \| High \| Some concerns \| High \| \| Park 2020 \| social support \| Some concerns \| Low \| High \| High \| Some concerns \| High \| \| Park 2020 \| positive emotions/affect \| Some concerns \| Low \| High \| High \| Some concerns \| High \| \| Park 2020 \| mindfulness \| Some concerns \| Low \| High \| High \| Some concerns \| High \| \| Park 2020 \| general distress \| Some concerns \| Low \| High \| High \| Some concerns \| High \| \| Pauls 2016 \| resilience \| Some concerns \| Low \| Low \| Low \| Some concerns \| Some concerns \| \| Pauls 2016 \| mindfulness \| Some concerns \| Low \| Low \| Low \| Some concerns \| Some concerns \| \| Profit 2021 \| depressive symptoms \| Some concerns \| Some concerns \| Low \| High \| Low \| High \| \| Profit 2021 \| happiness \| Some concerns \| Some concerns \| Low \| High \| Low \| High \| \| Pogrebtsova 2018 \| positive emotions/affect \| Some concerns \| Low \| Low \| High \| Some concerns \| High \| \| Proyer 2014 \| happiness \| Low \| Some concerns \| High \| Low \| Some concerns \| High \| \| Proyer 2014 \| depressive symptoms \| Low \| Some concerns \| High \| Low \| Some concerns \| High \| \| Puertas-Gonzalez 2022 \| stress symptoms \| Low \| Low \| High \| Low \| Low \| High \| \| Puertas-Gonzalez 2022 \| resilience \| Low \| Low \| High \| Low \| Low \| High \| \| Puertas-Gonzalez 2022 \| depressive symptoms \| Low \| Low \| High \| Low \| Low \| High \| \| Puertas-Gonzalez 2022 \| anxiety symptoms \| Low \| Low \| High \| Low \| Low \| High \| \| Sutarto 2012 \| depressive symptoms \| Some concerns \| Low \| Low \| High \| Some concerns \| High \| \| Sutarto 2012 \| anxiety symptoms \| Some concerns \| Low \| Low \| High \| Some concerns \| High \| \| Sutarto 2012 \| stress symptpms \| Some concerns \| Low \| Low \| High \| Some concerns \| High \| \| Pyne 2019 \| PTSD symptoms \| Some concerns \| Low \| High \| High \| Some concerns \| High \| \| Rackoff 2022 \| stress symptpms \| Low \| Low \| High \| High \| Low \| High \| \| Rackoff 2022 \| anxiety symptoms \| Low \| Low \| High \| High \| Low \| High \| \| Rackoff 2022 \| depressive symptoms \| Low \| Low \| High \| High \| Low \| High \| \| Ramey 2017 \| stress symptpms \| High \| Some concerns \| Low \| Some concerns \| Some concerns \| High \| \| Ramey 2017 \| resilience \| High \| Some concerns \| Low \| Some concerns \| Some concerns \| High \| \| Ramey 2017 \| PTSD symptoms \| High \| Some concerns \| Low \| Some concerns \| Some concerns \| High \| \| Ramey 2017 \| vitality \| High \| Some concerns \| Low \| Some concerns \| Some concerns \| High \| \| Ramey 2017 \| general distress \| High \| Some concerns \| Low \| Some concerns \| Some concerns \| High \| \| Riello 2021 \| anxiety symptoms \| Some concerns \| Some concerns \| High \| Low \| Low \| High \| \| Riello 2021 \| PTSD symptoms \| Some concerns \| Some concerns \| High \| Low \| Low \| High \| \| Riello 2021 \| quality of life \| Some concerns \| Some concerns \| High \| Low \| Low \| High \| \| Riello 2021 \| resilience \| Some concerns \| Some concerns \| High \| Low \| Low \| High \| \| Riello 2021 \| stress symptoms \| Some concerns \| Some concerns \| High \| Low \| Low \| High \| \| Roepke 2018 \| depressive symptoms \| Low \| Low \| High \| Low \| Some concerns \| High \| \| Roepke 2018 \| PTSD symptoms \| Low \| Low \| High \| Low \| Some concerns \| High \| \| Roepke 2018 \| posttraumatic growth \| Low \| Low \| High \| Low \| Some concerns \| High \| \| Roepke 2018 \| life satisfaction \| Low \| Low \| High \| Low \| Some concerns \| High \| \| Röhr 2021 \| PTSD symptoms \| Low \| Some concerns \| Low \| Some concerns \| High \| High \| \| Röhr 2021 \| depressive symptoms \| Low \| Some concerns \| Low \| Some concerns \| High \| High \| \| Röhr 2021 \| anxiety symptoms \| Low \| Some concerns \| Low \| Some concerns \| High \| High \| \| Röhr 2021 \| quality of life \| Low \| Some concerns \| Low \| Some concerns \| High \| High \| \| Röhr 2021 \| self-efficacy \| Low \| Some concerns \| Low \| Some concerns \| High \| High \| \| Röhr 2021 \| social support \| Low \| Some concerns \| Low \| Some concerns \| High \| High \| \| Röhr 2021 \| posttraumatic growth \| Low \| Some concerns \| Low \| Some concerns \| High \| High \| \| Roig 2020 \| resilience \| Low \| Low \| Low \| High \| Low \| High \| \| Roig 2020 \| wellbeing \| Low \| Low \| Low \| High \| Low \| High \| \| Roig 2020 \| resilience \| Low \| Low \| Low \| High \| Low \| High \| \| Roig 2020 \| depressive symptoms \| Low \| Low \| Low \| High \| Low \| High \| \| Roig 2020 \| stress symptoms \| Low \| Low \| Low \| High \| Low \| High \| \| Rose 2013 \| active coping \| Some concerns \| Low \| Low \| Low \| Some concerns \| Some concerns \| \| Rose 2013 \| stress symptoms \| Some concerns \| Low \| Low \| Low \| Some concerns \| Some concerns \| \| Rullo 2021 \| anxiety symptoms \| High \| Low \| Low \| Some concerns \| Some concerns \| High \| \| Rullo 2021 \| resilience \| High \| Low \| Low \| Some concerns \| Some concerns \| High \| \| Rullo 2021 \| happiness \| High \| Low \| Low \| Some concerns \| Some concerns \| High \| \| Rullo 2021 \| life satisfaction \| High \| Low \| Low \| Some concerns \| Some concerns \| High \| \| Rullo 2021 \| stress symptoms \| High \| Low \| Low \| Some concerns \| Some concerns \| High \| \| Schotanus-Dijkstra 2019 \| wellbeing \| Some concerns \| Low \| Low \| High \| Some concerns \| High \| \| Schotanus-Dijkstra 2019 \| anxiety symptoms \| Some concerns \| Low \| Low \| High \| Some concerns \| High \| \| Schotanus-Dijkstra 2019 \| depressive symptoms \| Some concerns \| Low \| Low \| High \| Some concerns \| High \| \| Schotanus-Dijkstra 2019 \| positive emotions/affect \| Some concerns \| Low \| Low \| High \| Some concerns \| High \| \| Schotanus-Dijkstra 2019 \| optimism \| Some concerns \| Low \| Low \| High \| Some concerns \| High \| \| Schotanus-Dijkstra 2019 \| self-compassion \| Some concerns \| Low \| Low \| High \| Some concerns \| High \| \| Schotanus-Dijkstra 2019 \| resilience \| Some concerns \| Low \| Low \| High \| Some concerns \| High \| \| Seear 2013 \| wellbeing \| Low \| Some concerns \| High \| High \| Some concerns \| High \| \| Seear 2013 \| positive emotions/affect \| Low \| Some concerns \| High \| High \| Some concerns \| High \| \| Seligman 2007 \| depressive symptoms \| Some concerns \| Some concerns \| Low \| High \| Some concerns \| High \| \| Seligman 2007 \| anxiety symptoms \| Some concerns \| Some concerns \| Low \| High \| Some concerns \| High \| \| Seligman 2007 \| life satisfaction \| Some concerns \| Some concerns \| Low \| High \| Some concerns \| High \| \| Seligman 2007 \| wellbeing \| Some concerns \| Some concerns \| Low \| High \| Some concerns \| High \| \| Sim 2020 \| quality of life \| Low \| Low \| Low \| Some concerns \| Low \| Low \| \| Spilg 2022 \| resilience \| Low \| Some concerns \| Low \| High \| Low \| High \| \| Spilg 2022 \| happiness \| Low \| Some concerns \| Low \| High \| Low \| High \| \| Spilg 2022 \| stress symptoms \| Low \| Some concerns \| Low \| High \| Low \| High \| \| Stephens 2012 \| stress symptoms \| Low \| Some concerns \| Low \| Low \| Some concerns \| Some concerns \| \| Stephens 2012 \| social support \| Low \| Some concerns \| Low \| Low \| Some concerns \| Some concerns \| \| Stephens 2012 \| resilience \| Low \| Some concerns \| Low \| Low \| Some concerns \| Some concerns \| \| Stockton 2014 \| PTSD symptoms \| Some concerns \| Low \| High \| Low \| Some concerns \| High \| \| Stockton 2014 \| posttraumatic growth \| Some concerns \| Low \| High \| Low \| Some concerns \| High \| \| Stockton 2014 \| wellbeing \| Some concerns \| Low \| High \| Low \| Some concerns \| High \| \| Tagalidou 2019 \| depressive symptoms \| Low \| Low \| Some concerns \| Low \| Some concerns \| Some concerns \| \| van Berkel 2014 \| mental health \| High \| Some concerns \| Low \| High \| Low \| High \| \| van Berkel 2014 \| mindfulness \| High \| Some concerns \| Low \| High \| Low \| High \| \| van der Houwen 2010 \| depressive symptoms \| Low \| High \| Some concerns \| High \| High \| High \| \| van der Houwen 2010 \| positive emotions/affect \| Low \| High \| Some concerns \| High \| High \| High \| \| van der Meer 2020 \| PTSD symptoms \| Low \| Low \| Low \| High \| Some concerns \| High \| \| van der Meer 2020 \| resilience \| Low \| Low \| Low \| High \| Some concerns \| High \| \| van der Meer 2020 \| social support \| Low \| Low \| Low \| High \| Some concerns \| High \| \| Villani 2013 \| anxiety symptoms \| Some concerns \| Low \| High \| Low \| Some concerns \| High \| \| Villani 2013 \| active coping \| Some concerns \| Low \| High \| Low \| Some concerns \| High \| \| Wijesekera 2019 \| depressive symptoms \| Some concerns \| Some concerns \| Some concerns \| High \| Some concerns \| High \| \| Wijesekera 2019 \| anxiety symptoms \| Some concerns \| Some concerns \| Some concerns \| High \| Some concerns \| High \| \| Wijesekera 2019 \| PTSD symptoms \| Some concerns \| Some concerns \| Some concerns \| High \| Some concerns \| High \| \| Yousefi 2022 \| resilience \| Low \| Low \| Low \| Some concerns \| Some concerns \| High \| \| Yousefi 2022 \| wellbeing \| Low \| Low \| Low \| Some concerns \| Some concerns \| High \| \| Zahedifar 2021 \| anxiety symptoms \| Some concerns \| Low \| High \| High \| High \| High \| \| Zahedifar 2021 \| quality of life \| Some concerns \| Low \| High \| High \| High \| High \| |
| --- | --- | --- | --- | --- | --- | --- | --- | --- | --- | --- | --- | --- | --- | --- | --- | --- | --- | --- | --- | --- | --- | --- | --- | --- | --- | --- | --- | --- | --- | --- | --- | --- | --- | --- | --- | --- | --- | --- | --- | --- | --- | --- | --- | --- | --- | --- | --- | --- | --- | --- | --- | --- | --- | --- | --- | --- | --- | --- | --- | --- | --- | --- | --- | --- | --- | --- | --- | --- | --- | --- | --- | --- | --- | --- | --- | --- | --- | --- | --- | --- | --- | --- | --- | --- | --- | --- | --- | --- | --- | --- | --- | --- | --- | --- | --- | --- | --- | --- | --- | --- | --- | --- | --- | --- | --- | --- | --- | --- | --- | --- | --- | --- | --- | --- | --- | --- | --- | --- | --- | --- | --- | --- | --- | --- | --- | --- | --- | --- | --- | --- | --- | --- | --- | --- | --- | --- | --- | --- | --- | --- | --- | --- | --- | --- | --- | --- | --- | --- | --- | --- | --- | --- | --- | --- | --- | --- | --- | --- | --- | --- | --- | --- | --- | --- | --- | --- | --- | --- | --- | --- | --- | --- | --- | --- | --- | --- | --- | --- | --- | --- | --- | --- | --- | --- | --- | --- | --- | --- | --- | --- | --- | --- | --- | --- | --- | --- | --- | --- | --- | --- | --- | --- | --- | --- | --- | --- | --- | --- | --- | --- | --- | --- | --- | --- | --- | --- | --- | --- | --- | --- | --- | --- | --- | --- | --- | --- | --- | --- | --- | --- | --- | --- | --- | --- | --- | --- | --- | --- | --- | --- | --- | --- | --- | --- | --- | --- | --- | --- | --- | --- | --- | --- | --- | --- | --- | --- | --- | --- | --- | --- | --- | --- | --- | --- | --- | --- | --- | --- | --- | --- | --- | --- | --- | --- | --- | --- | --- | --- | --- | --- | --- | --- | --- | --- | --- | --- | --- | --- | --- | --- | --- | --- | --- | --- | --- | --- | --- | --- | --- | --- | --- | --- | --- | --- | --- | --- | --- | --- | --- | --- | --- | --- | --- | --- | --- | --- | --- | --- | --- | --- | --- | --- | --- | --- | --- | --- | --- | --- | --- | --- | --- | --- | --- | --- | --- | --- | --- | --- | --- | --- | --- | --- | --- | --- | --- | --- | --- | --- | --- | --- | --- | --- | --- | --- | --- | --- | --- | --- | --- | --- | --- | --- | --- | --- | --- | --- | --- | --- | --- | --- | --- | --- | --- | --- | --- | --- | --- | --- | --- | --- | --- | --- | --- | --- | --- | --- | --- | --- | --- | --- | --- | --- | --- | --- | --- | --- | --- | --- | --- | --- | --- | --- | --- | --- | --- | --- | --- | --- | --- | --- | --- | --- | --- | --- | --- | --- | --- | --- | --- | --- | --- | --- | --- | --- | --- | --- | --- | --- | --- | --- | --- | --- | --- | --- | --- | --- | --- | --- | --- | --- | --- | --- | --- | --- | --- | --- | --- | --- | --- | --- | --- | --- | --- | --- | --- | --- | --- | --- | --- | --- | --- | --- | --- | --- | --- | --- | --- | --- | --- | --- | --- | --- | --- | --- | --- | --- | --- | --- | --- | --- | --- | --- | --- | --- | --- | --- | --- | --- | --- | --- | --- | --- | --- | --- | --- | --- | --- | --- | --- | --- | --- | --- | --- | --- | --- | --- | --- | --- | --- | --- | --- | --- | --- | --- | --- | --- | --- | --- | --- | --- | --- | --- | --- | --- | --- | --- | --- | --- | --- | --- | --- | --- | --- | --- | --- | --- | --- | --- | --- | --- | --- | --- | --- | --- | --- | --- | --- | --- | --- | --- | --- | --- | --- | --- | --- | --- | --- | --- | --- | --- | --- | --- | --- | --- | --- | --- | --- | --- | --- | --- | --- | --- | --- | --- | --- | --- | --- | --- | --- | --- | --- | --- | --- | --- | --- | --- | --- | --- | --- | --- | --- | --- | --- | --- | --- | --- | --- | --- | --- | --- | --- | --- | --- | --- | --- | --- | --- | --- | --- | --- | --- | --- | --- | --- | --- | --- | --- | --- | --- | --- | --- | --- | --- | --- | --- | --- | --- | --- | --- | --- | --- | --- | --- | --- | --- | --- | --- | --- | --- | --- | --- | --- | --- | --- | --- | --- | --- | --- | --- | --- | --- | --- | --- | --- | --- | --- | --- | --- | --- | --- | --- | --- | --- | --- | --- | --- | --- | --- | --- | --- | --- | --- | --- | --- | --- | --- | --- | --- | --- | --- | --- | --- | --- | --- | --- | --- | --- | --- | --- | --- | --- | --- | --- | --- | --- | --- | --- | --- | --- | --- | --- | --- | --- | --- | --- | --- | --- | --- | --- | --- | --- | --- | --- | --- | --- | --- | --- | --- | --- | --- | --- | --- | --- | --- | --- | --- | --- | --- | --- | --- | --- | --- | --- | --- | --- | --- | --- | --- | --- | --- | --- | --- | --- | --- | --- | --- | --- | --- | --- | --- | --- | --- | --- | --- | --- | --- | --- | --- | --- | --- | --- | --- | --- | --- | --- | --- | --- | --- | --- | --- | --- | --- | --- | --- | --- | --- | --- | --- | --- | --- | --- | --- | --- | --- | --- | --- | --- | --- | --- | --- | --- | --- | --- | --- | --- | --- | --- | --- | --- | --- | --- | --- | --- | --- | --- | --- | --- | --- | --- | --- | --- | --- | --- | --- | --- | --- | --- | --- | --- | --- | --- | --- | --- | --- | --- | --- | --- | --- | --- | --- | --- | --- | --- | --- | --- | --- | --- | --- | --- | --- | --- | --- | --- | --- | --- | --- | --- | --- | --- | --- | --- | --- | --- | --- | --- | --- | --- | --- | --- | --- | --- | --- | --- | --- | --- | --- | --- | --- | --- | --- | --- | --- | --- | --- | --- | --- | --- | --- | --- | --- | --- | --- | --- | --- | --- | --- | --- | --- | --- | --- | --- | --- | --- | --- | --- | --- | --- | --- | --- | --- | --- | --- | --- | --- | --- | --- | --- | --- | --- | --- | --- | --- | --- | --- | --- | --- | --- | --- | --- | --- | --- | --- | --- | --- | --- | --- | --- | --- | --- | --- | --- | --- | --- | --- | --- | --- | --- | --- | --- | --- | --- | --- | --- | --- | --- | --- | --- | --- | --- | --- | --- | --- | --- | --- | --- | --- | --- | --- | --- | --- | --- | --- | --- | --- | --- | --- | --- | --- | --- | --- | --- | --- | --- | --- | --- | --- | --- | --- | --- | --- | --- | --- | --- | --- | --- | --- | --- | --- | --- | --- | --- | --- | --- | --- | --- | --- | --- | --- | --- | --- | --- | --- | --- | --- | --- | --- | --- | --- | --- | --- | --- | --- | --- | --- | --- | --- | --- | --- | --- | --- | --- | --- | --- | --- | --- | --- | --- | --- | --- | --- | --- | --- | --- | --- | --- | --- | --- | --- | --- | --- | --- | --- | --- | --- | --- | --- | --- | --- | --- | --- | --- | --- | --- | --- | --- | --- | --- | --- | --- | --- | --- | --- | --- | --- | --- | --- | --- | --- | --- | --- | --- | --- | --- | --- | --- | --- | --- | --- | --- | --- | --- | --- | --- | --- | --- | --- | --- | --- | --- | --- | --- | --- | --- | --- | --- | --- | --- | --- | --- | --- | --- | --- | --- | --- | --- | --- | --- | --- | --- | --- | --- | --- | --- | --- | --- | --- | --- | --- | --- | --- | --- | --- | --- | --- | --- | --- | --- | --- | --- | --- | --- | --- | --- | --- | --- | --- | --- | --- | --- | --- | --- | --- | --- | --- | --- | --- | --- | --- | --- | --- | --- | --- | --- | --- | --- | --- | --- | --- | --- | --- | --- | --- | --- | --- | --- | --- | --- | --- | --- | --- | --- | --- | --- | --- | --- | --- | --- | --- | --- | --- | --- | --- | --- | --- | --- | --- | --- | --- | --- | --- | --- | --- | --- | --- | --- | --- | --- | --- | --- | --- | --- | --- | --- | --- | --- | --- | --- | --- | --- | --- | --- | --- | --- | --- | --- | --- | --- | --- | --- | --- | --- | --- | --- | --- | --- | --- | --- | --- | --- | --- | --- | --- | --- | --- | --- | --- | --- | --- | --- | --- | --- | --- | --- | --- | --- | --- | --- | --- | --- | --- | --- | --- | --- | --- | --- | --- | --- | --- | --- | --- | --- | --- | --- | --- | --- | --- | --- | --- | --- | --- | --- | --- | --- | --- | --- | --- | --- | --- | --- | --- | --- | --- | --- | --- | --- | --- | --- | --- | --- | --- | --- | --- | --- | --- | --- | --- | --- | --- | --- | --- | --- | --- | --- | --- | --- | --- | --- | --- | --- | --- | --- | --- | --- | --- | --- | --- | --- | --- | --- | --- | --- | --- | --- | --- | --- | --- | --- | --- | --- | --- | --- | --- | --- | --- | --- | --- | --- | --- | --- | --- | --- | --- | --- | --- | --- | --- | --- | --- | --- | --- | --- | --- | --- | --- | --- | --- | --- | --- | --- | --- | --- | --- | --- | --- | --- | --- | --- | --- | --- | --- | --- | --- | --- | --- | --- | --- | --- | --- | --- | --- | --- | --- | --- | --- | --- | --- | --- | --- | --- | --- | --- | --- | --- | --- | --- | --- | --- | --- | --- | --- | --- | --- | --- | --- | --- | --- | --- | --- | --- | --- | --- | --- | --- | --- | --- | --- | --- | --- | --- | --- | --- | --- | --- | --- | --- | --- | --- | --- | --- | --- | --- | --- | --- | --- | --- | --- | --- | --- | --- | --- | --- | --- | --- | --- | --- | --- | --- | --- | --- | --- | --- | --- | --- | --- | --- | --- | --- | --- | --- | --- | --- | --- | --- | --- | --- | --- | --- | --- | --- | --- | --- | --- | --- | --- | --- | --- | --- | --- | --- | --- | --- | --- | --- | --- | --- | --- | --- | --- | --- | --- | --- | --- | --- | --- | --- | --- | --- | --- | --- | --- | --- | --- | --- | --- | --- | --- | --- | --- | --- | --- | --- | --- | --- | --- | --- | --- | --- | --- | --- | --- | --- | --- | --- | --- | --- | --- | --- | --- | --- | --- | --- | --- | --- | --- | --- | --- | --- | --- | --- | --- | --- | --- | --- | --- | --- | --- | --- | --- | --- | --- | --- | --- | --- | --- | --- | --- | --- | --- | --- | --- | --- | --- | --- | --- | --- | --- | --- | --- | --- | --- | --- | --- | --- | --- | --- | --- | --- | --- | --- | --- | --- | --- | --- | --- | --- | --- | --- | --- | --- | --- | --- | --- | --- | --- | --- | --- | --- | --- | --- | --- | --- | --- | --- | --- | --- | --- | --- | --- | --- | --- | --- | --- | --- | --- | --- | --- | --- | --- | --- | --- | --- | --- | --- | --- | --- | --- | --- | --- | --- | --- | --- | --- | --- | --- | --- | --- | --- | --- | --- | --- | --- | --- | --- | --- | --- | --- | --- | --- | --- | --- | --- | --- | --- | --- | --- | --- | --- | --- | --- | --- | --- | --- | --- | --- | --- | --- | --- | --- | --- | --- | --- | --- | --- | --- | --- | --- | --- | --- | --- | --- | --- | --- | --- | --- | --- | --- | --- | --- | --- | --- | --- | --- | --- | --- | --- | --- | --- | --- | --- | --- | --- | --- | --- | --- | --- | --- | --- | --- | --- | --- | --- | --- | --- | --- | --- | --- | --- | --- | --- | --- | --- | --- | --- | --- | --- | --- | --- | --- | --- | --- | --- | --- | --- | --- | --- | --- | --- | --- | --- | --- | --- | --- | --- | --- | --- | --- | --- | --- | --- | --- | --- | --- | --- | --- | --- | --- | --- | --- | --- | --- | --- | --- | --- | --- | --- | --- | --- | --- | --- | --- | --- | --- | --- | --- | --- | --- | --- | --- | --- | --- | --- | --- | --- | --- | --- | --- | --- | --- | --- | --- | --- | --- | --- | --- | --- | --- | --- | --- | --- | --- | --- | --- | --- | --- | --- | --- | --- | --- | --- | --- | --- | --- | --- | --- | --- | --- | --- | --- | --- | --- | --- | --- | --- | --- | --- | --- | --- | --- | --- | --- | --- | --- | --- | --- | --- | --- | --- | --- | --- | --- | --- | --- | --- | --- | --- | --- | --- | --- | --- | --- | --- | --- | --- | --- | --- | --- | --- | --- | --- | --- | --- | --- | --- | --- | --- | --- | --- | --- | --- | --- | --- | --- | --- | --- | --- | --- | --- | --- | --- | --- | --- | --- | --- | --- | --- | --- | --- | --- | --- | --- | --- | --- | --- | --- | --- | --- | --- | --- | --- | --- | --- | --- | --- | --- | --- | --- | --- | --- | --- | --- | --- | --- | --- | --- | --- | --- | --- | --- | --- | --- | --- | --- | --- | --- | --- | --- | --- | --- | --- | --- | --- | --- | --- | --- | --- | --- | --- | --- | --- | --- | --- | --- | --- | --- | --- | --- | --- | --- | --- | --- | --- | --- | --- | --- | --- | --- | --- | --- | --- | --- | --- | --- | --- | --- | --- | --- | --- | --- | --- | --- | --- | --- | --- | --- | --- | --- | --- | --- | --- | --- | --- | --- | --- | --- | --- | --- | --- | --- | --- | --- | --- | --- | --- | --- | --- | --- | --- | --- | --- | --- | --- | --- | --- | --- | --- | --- | --- | --- | --- | --- | --- | --- | --- | --- | --- | --- | --- | --- | --- | --- | --- | --- | --- | --- | --- | --- | --- | --- | --- | --- | --- | --- | --- | --- | --- | --- | --- | --- | --- | --- | --- | --- | --- | --- | --- | --- | --- | --- | --- | --- | --- | --- | --- | --- | --- | --- | --- | --- | --- | --- | --- | --- | --- | --- | --- | --- | --- | --- | --- | --- | --- | --- | --- | --- | --- | --- | --- | --- | --- | --- | --- | --- | --- | --- | --- | --- | --- | --- | --- | --- | --- | --- | --- | --- | --- | --- | --- | --- | --- | --- | --- | --- | --- | --- | --- | --- | --- | --- | --- | --- | --- | --- | --- | --- | --- | --- | --- | --- | --- | --- | --- | --- | --- | --- | --- | --- | --- | --- | --- | --- | --- | --- | --- | --- | --- | --- | --- | --- | --- | --- | --- | --- | --- | --- | --- | --- | --- | --- | --- | --- | --- | --- | --- | --- | --- | --- | --- | --- | --- | --- | --- | --- | --- | --- | --- | --- | --- | --- | --- | --- | --- | --- | --- | --- | --- | --- | --- | --- | --- | --- | --- | --- | --- | --- | --- | --- | --- | --- | --- | --- | --- | --- | --- | --- | --- | --- | --- | --- | --- | --- | --- | --- | --- | --- | --- | --- | --- | --- | --- | --- | --- | --- | --- | --- | --- | --- | --- | --- | --- | --- | --- | --- | --- | --- | --- | --- | --- | --- | --- | --- | --- | --- | --- | --- | --- | --- | --- | --- | --- | --- | --- | --- | --- | --- | --- | --- | --- | --- | --- | --- | --- | --- | --- | --- | --- | --- | --- | --- | --- | --- | --- | --- | --- | --- | --- | --- | --- | --- | --- | --- | --- | --- | --- | --- | --- | --- | --- | --- | --- | --- | --- | --- | --- | --- | --- | --- | --- | --- | --- | --- | --- | --- | --- | --- | --- | --- | --- | --- | --- | --- | --- | --- | --- | --- | --- | --- | --- | --- | --- | --- | --- | --- | --- | --- | --- | --- | --- | --- | --- | --- | --- | --- | --- | --- | --- | --- | --- | --- | --- | --- | --- | --- | --- | --- | --- | --- | --- | --- | --- | --- | --- | --- | --- | --- | --- | --- | --- | --- | --- | --- | --- | --- | --- | --- | --- | --- | --- | --- | --- | --- | --- | --- | --- | --- | --- | --- | --- | --- | --- | --- | --- | --- | --- | --- | --- | --- | --- | --- | --- | --- | --- | --- | --- | --- | --- | --- | --- | --- | --- | --- | --- | --- | --- | --- | --- | --- | --- | --- | --- | --- | --- | --- | --- | --- | --- | --- | --- | --- | --- | --- | --- | --- | --- | --- | --- | --- | --- | --- | --- | --- | --- | --- | --- | --- | --- | --- | --- | --- | --- | --- | --- | --- | --- | --- | --- | --- | --- | --- | --- | --- | --- | --- | --- | --- | --- | --- | --- | --- | --- | --- | --- | --- | --- | --- | --- | --- | --- | --- | --- | --- | --- | --- | --- | --- | --- | --- | --- | --- | --- | --- | --- | --- | --- | --- | --- | --- | --- | --- | --- | --- | --- | --- | --- | --- | --- | --- | --- | --- | --- | --- | --- | --- | --- | --- | --- | --- | --- | --- | --- | --- | --- | --- | --- | --- | --- | --- | --- | --- | --- | --- | --- | --- | --- | --- | --- | --- | --- | --- | --- | --- | --- | --- | --- | --- | --- | --- | --- | --- | --- | --- | --- | --- | --- | --- | --- | --- | --- | --- | --- | --- | --- | --- | --- | --- | --- | --- | --- | --- | --- | --- | --- | --- | --- | --- | --- | --- | --- | --- | --- | --- | --- | --- | --- | --- | --- | --- | --- | --- | --- | --- | --- | --- | --- | --- | --- | --- | --- | --- | --- | --- | --- | --- | --- | --- | --- | --- | --- | --- | --- | --- | --- | --- | --- | --- | --- | --- | --- | --- | --- | --- | --- | --- | --- | --- | --- | --- | --- | --- | --- | --- | --- | --- | --- | --- | --- | --- | --- | --- | --- | --- | --- | --- | --- | --- | --- | --- | --- | --- | --- | --- | --- | --- | --- | --- | --- | --- | --- | --- | --- | --- | --- | --- | --- | --- | --- | --- | --- | --- | --- | --- | --- | --- | --- | --- | --- | --- | --- | --- | --- | --- | --- | --- | --- | --- | --- | --- | --- | --- | --- | --- | --- | --- | --- | --- | --- | --- | --- | --- | --- | --- | --- | --- | --- | --- | --- | --- | --- | --- | --- | --- | --- | --- | --- | --- | --- | --- | --- | --- | --- | --- | --- | --- | --- | --- | --- | --- | --- | --- | --- | --- | --- | --- | --- | --- | --- | --- | --- | --- | --- | --- | --- | --- | --- | --- | --- | --- | --- | --- | --- | --- | --- | --- | --- | --- | --- | --- | --- | --- | --- | --- | --- | --- | --- | --- | --- | --- | --- | --- | --- | --- | --- | --- | --- | --- | --- | --- | --- | --- | --- | --- | --- | --- | --- | --- | --- | --- | --- | --- |

### Supplementary Data 5. Publication bias analyses – Funnel plots at post-intervention assessments

**Figure 1** Contour-enhanced funnel plots of the analyses on mental distress, positive mental health and resilience factors at post-intervention assessments

*Note.* Contour-enhanced funnel plots for the analyses on mental distress, positive mental health and resilience factors at post-intervention assessment. Contour-enhanced funnel plots are used to highlight significance levels of each effect size in the plot and allow to examine whether studies are missing in a specific area of the plot, that is, the non-significant area or findings pointing into the opposite effect direction. For the present analyses, none of the plots points to a strong impact of publication bias, with most effect estimates falling at both sides of the significance border areas.

Effect estimates highlighted in red were further examined in sensitivity analyses (see SMX).

### Supplementary Data 6. Publication bias analyses – Funnel plots at follow-up assessments

**Figure 2** Contour-enhanced funnel plots of the analyses on mental distress, positive mental health and resilience factors at follow-up assessments

*Note.* Contour-enhanced funnel plots for the analyses on mental distress, positive mental health and resilience factors at post-intervention assessment. Contour-enhanced funnel plots are used to highlight significance levels of each effect size in the plot and allow to examine whether studies are missing in a specific area of the plot, that is, the non-significant area or findings pointing into the opposite effect direction. For the present analyses, none of the plots points to a strong impact of publication bias, with most effect estimates falling at both sides of the significance border areas.

Effect estimates highlighted in red were further examined in sensitivity analyses (see SMX).

### Supplementary Data 7. Forest plots for mental distress at post-intervention assessment

**Figure 3** Forest plot of the meta-analyses on mental distress at post-intervention assessment

*Note*. CI = confidence interval; df = degree of freedom; Q = Cochran’s *Q* statistic with *p* value; RE = random effects; SMD = standardized mean difference.

### Supplementary Data 8. Forest plots for positive mental health at post-intervention assessment

**Figure 4** Forest plot of the meta-analyses on positive mental health at post-intervention assessment

*Note*. CI = confidence interval; df = degree of freedom; Q = Cochran’s *Q* statistic with *p* value; RE = random effects; SMD = standardized mean difference.

### Supplementary Data 9. Forest plots for resilience factors at post-intervention assessment

**Figure 5** Forest plot of the meta-analyses on resilience factors at post-intervention assessment

*Note*. CI = confidence interval; df = degree of freedom; Q = Cochran’s *Q* statistic with *p* value; RE = random effects; SMD = standardized mean difference.

### Supplementary Data 10. GRADE ratings at post-intervention assessments

**Table 4** GRADE assessment for mental distress (post assessment)

| **GRADE criteria** | **Rating** | **Notes** | **Certainty of evidence** |
| --- | --- | --- | --- |
| **Study design** | **RCT (high quality)**  Non-RCT (low quality) | / | ⊕◯◯◯  Very low |
| **Risk of Bias**  (Cochrane Risk of Bias) | serious (-1)  **very serious (-2)** | Very serious limitations due to lack of blinding of outcome assessors and high overall risk of bias for a large proportion of studies, with only one study being rated at overall low risk |  |
| **Inconsistency** | **serious (-1)**  very serious (-2) | Unexplained inconsistency (*I^2^* ≥ 41.3%) for all outcome types |  |
| **Indirectness** | **serious (-1)**  very serious (-2) | Studies limited to certain participants (young and middle-aged adults) and certain comparators (mostly passive and/or low intensity) |  |
| **Imprecision** | serious (-1)  very serious (-2) | / |  |
| **Publication Bias** | Undetected  Strongly suspected (-1) | / |  |
| **Other**  (upgrading factor) | Large effect (+1 or +2)  Dose response (+1 or +2)  No plausible confounding (+1 or +2) | / |  |

**Source**: Modified after Ryan R, Hill S (2016) How to GRADE the quality of the evidence. Cochrane Consumers and Communication Group, available at http://cccrg.cochrane.org/author-resources. Version 3.0 December 2016

**Table 5** GRADE assessment for positive mental health (post assessment)

| **GRADE criteria** | **Rating** | **Notes** | **Certainty of evidence** |
| --- | --- | --- | --- |
| **Study design** | **RCT (high quality)**  Non-RCT (low quality) | / | ⊕◯◯◯  Very low |
| **Risk of Bias**  (Cochrane Risk of Bias) | serious (-1)  **very serious (-2)** | Very serious limitations due to lack of blinding of outcome assessors and high overall risk of bias for a large proportion of studies, with only one study being rated at overall low risk |  |
| **Inconsistency** | **serious (-1)**  very serious (-2) | Unexplained inconsistency (*I^2^* ≥ 67.5%) for all outcome types |  |
| **Indirectness** | **serious (-1)**  very serious (-2) | Studies limited to certain participants (young and middle-aged adults) and certain comparators (mostly passive and/or low intensity) |  |
| **Imprecision** | serious (-1)  very serious (-2) | / |  |
| **Publication Bias** | Undetected  Strongly suspected (-1) | / |  |
| **Other**  (upgrading factor) | Large effect (+1 or +2)  Dose response (+1 or +2)  No plausible confounding (+1 or +2) | / |  |

**Source**: Modified after Ryan R, Hill S (2016) How to GRADE the quality of the evidence. Cochrane Consumers and Communication Group, available at http://cccrg.cochrane.org/author-resources. Version 3.0 December 2016

**Table 6** GRADE assessment for resilience factors (post assessment)

| **GRADE criteria** | **Rating** | **Notes** | **Certainty of evidence** |
| --- | --- | --- | --- |
| **Study design** | **RCT (high quality)**  Non-RCT (low quality) | / | ⊕◯◯◯  Very low |
| **Risk of Bias**  (Cochrane Risk of Bias) | serious (-1)  **very serious (-2)** | Very serious limitations due to lack of blinding of outcome assessors and high overall risk of bias for a large proportion of studies, with only one study being rated at overall low risk |  |
| **Inconsistency** | **serious (-1)**  very serious (-2) | Unexplained inconsistency (*I^2^* ≥ 48.2%) for all outcome types |  |
| **Indirectness** | **serious (-1)**  very serious (-2) | Studies limited to certain participants (young and middle-aged adults) and certain comparators (mostly passive and/or low intensity) |  |
| **Imprecision** | serious (-1)  very serious (-2) | / |  |
| **Publication Bias** | **Undetected**  Strongly suspected (-1) | / |  |
| **Other**  (upgrading factor) | Large effect (+1 or +2)  Dose response (+1 or +2)  No Plausible confounding (+1 or +2) | / |  |

**Source**: Modified after Ryan R, Hill S (2016) How to GRADE the quality of the evidence. Cochrane Consumers and Communication Group, available at http://cccrg.cochrane.org/author-resources. Version 3.0 December 2016

### Supplementary Data 11. Forest plots for mental distress at follow-up assessment

**Figure 6** Forest plot of the meta-analyses on resilience factors at post-intervention assessment

*Note*. Forest plot of the multilevel meta-analysis on mental distress at follow-up assessment. Negative effect estimates indicate an effect favoring digital over comparators.

CI = confidence interval; df = degree of freedom; Q = Cochran’s *Q* statistic with *p* value; RE = random effects; SMD = standardized mean difference.

### Supplementary Data 12. Forest plots for positive mental health at follow-up assessment

**Figure 7** Forest plot of the meta-analyses on positive mental health at post-intervention assessment

*Note*. Forest plot of the multilevel meta-analysis on mental distress at follow-up assessment. Negative effect estimates indicate an effect favoring digital over comparators.

CI = confidence interval; df = degree of freedom; Q = Cochran’s *Q* statistic with *p* value; RE = random effects; SMD = standardized mean difference.

### Supplementary Data 13. Forest plots for resilience factors at follow-up assessment

**Figure 8** Forest plot of the meta-analyses on resilience factors at post-intervention assessment

*Note*. Forest plot of the multilevel meta-analysis on mental distress at follow-up assessment. Negative effect estimates indicate an effect favoring digital over comparators.

CI = confidence interval; df = degree of freedom; Q = Cochran’s *Q* statistic with *p* value; RE = random effects; SMD = standardized mean difference.

### Supplementary Data 14. GRADE ratings at follow-up assessments

**Table 7** GRADE assessment for mental distress (follow-up)

| **GRADE criteria** | **Rating** | **Notes** | **Certainty of evidence** |
| --- | --- | --- | --- |
| **Study design** | **RCT (high quality)**  Non-RCT (low quality) | / | ⊕◯◯◯  Very low |
| **Risk of Bias**  (Cochrane Risk of Bias) | serious (-1)  **very serious (-2)** | Very serious limitations due to lack of blinding of outcome assessors and high overall risk of bias for a large proportion of studies, with only one study being rated at overall low risk |  |
| **Inconsistency** | **serious (-1)**  very serious (-2) | Unexplained inconsistency (*I^2^* ≥ 61.0%) for all outcome types |  |
| **Indirectness** | **serious (-1)**  very serious (-2) | Studies limited to certain participants (young and middle-aged adults) and certain comparators (mostly passive and/or low intensity) |  |
| **Imprecision** | serious (-1)  very serious (-2) | / |  |
| **Publication Bias** | Undetected  Strongly suspected (-1) | / |  |
| **Other**  (upgrading factor) | Large effect (+1 or +2)  Dose response (+1 or +2)  No Plausible confounding (+1 or +2) | / |  |

**Source**: Modified after Ryan R, Hill S (2016) How to GRADE the quality of the evidence. Cochrane Consumers and Communication Group, available at http://cccrg.cochrane.org/author-resources. Version 3.0 December 2016

**Table 8** GRADE assessment for positive mental health (follow-up)

| **GRADE criteria** | **Rating** | **Notes** | **Certainty of evidence** |
| --- | --- | --- | --- |
| **Study design** | **RCT (high quality)**  Non-RCT (low quality) | / | ⊕◯◯◯  Very low |
| **Risk of Bias**  (Cochrane Risk of Bias) | serious (-1)  **very serious (-2)** | Very serious limitations due to lack of blinding of outcome assessors and high overall risk of bias for a large proportion of studies, with only one study being rated at overall low risk |  |
| **Inconsistency** | **serious (-1)**  very serious (-2) | Unexplained inconsistency (*I^2^* ≥ 56.7%) for all outcome types |  |
| **Indirectness** | **serious (-1)**  very serious (-2) | Studies limited to certain participants (young and middle-aged adults) and certain comparators (mostly passive and/or low intensity) |  |
| **Imprecision** | serious (-1)  very serious (-2) | / |  |
| **Publication Bias** | **Undetected**  Strongly suspected (-1) | / |  |
| **Other**  (upgrading factor) | Large effect (+1 or +2)  Dose response (+1 or +2)  No Plausible confounding (+1 or +2) | / |  |

**Source**: Modified after Ryan R, Hill S (2016) How to GRADE the quality of the evidence. Cochrane Consumers and Communication Group, available at http://cccrg.cochrane.org/author-resources. Version 3.0 December 2016

**Table 9** GRADE assessment for resilience factors (follow-up)

| **GRADE criteria** | **Rating** | **Notes** | **Certainty of evidence** |
| --- | --- | --- | --- |
| **Study design** | **RCT (high quality)**  Non-RCT (low quality) | / | ⊕◯◯◯  Very low |
| **Risk of Bias**  (Cochrane Risk of Bias) | serious (-1)  **very serious (-2)** | Very serious limitations due to lack of blinding of outcome assessors and high overall risk of bias for a large proportion of studies, with only one study being rated at overall low risk |  |
| **Inconsistency** | **serious (-1)**  very serious (-2) | Unexplained inconsistency (*I^2^* ≥ 40.3%) for all outcome types |  |
| **Indirectness** | **serious (-1)**  very serious (-2) | Studies limited to certain participants (young and middle-aged adults) and certain comparators (mostly passive and/or low intensity) |  |
| **Imprecision** | serious (-1)  very serious (-2) | / |  |
| **Publication Bias** | **Undetected**  Strongly suspected (-1) | / |  |
| **Other**  (upgrading factor) | Large effect (+1 or +2)  Dose response (+1 or +2)  No Plausible confounding (+1 or +2) | / |  |

**Source**: Modified after Ryan R, Hill S (2016) How to GRADE the quality of the evidence. Cochrane Consumers and Communication Group, available at http://cccrg.cochrane.org/author-resources. Version 3.0 December 2016

### Supplementary Data 15. Moderator analyses for follow-up assessments

**Table 10** Results of Moderator Analyses at Follow-Up Assessment

|  | **Mental distress** | | **Positive mental health** | | **Resilience factors** | |
| --- | --- | --- | --- | --- | --- | --- |
|  | *n/k* | *M*(SMD) [95% CI], *p* | *n/k* | *M*(SMD) [95% CI], *p* | *n/k* | *M*(SMD) [95% CI], *p* |
| ***Sociodemographic characteristics*** |  |  |  |  |  |  |
| Mean age | 36/60 | *QM*(1) = 4.32, *p* = .045* | 34/53 | *QM*(1) = 7.59, *p* = .010* | 17/24 | *QM*(1) = 0.52, *p* = .484 |
| Gender (% women) | 37/61 | *QM*(1) = 0.63, *p* = .433 | 35/56 | *QM*(1) = 0.29, *p* = .593 | 17/24 | *QM*(1) = 4.38, *p* = .054 |
| ***Population type*** (Military vs. University/College vs. Workplace) | | | | | | |
| Omnibus moderator test | 24/42 | *QM*(2) = 1.53, *p* = .239 | 21/30 | *QM*(2) = 0.04, *p* = .851 | 13/16 | *QM*(1) = 1.09, *p* = .320 |
| ***Delivery format*** (eHealth vs. mHealth vs. mixed) |  |  |  |  |  |  |
| Omnibus moderator test | 37/63 | *QM*(1) = 0.47, *p* = .498 | 20/29 | *QM*(1) = 0.25, *p* = .777 | 19/26 | *QM*(2) = 3.25, *p* = .067 |
| ***Theoretical foundation*** (CBT vs. Coping Literature vs. Mindfulness vs. Positive Psychology vs. mixed) | | | | | | |
| Omnibus moderator test | 25/46 | *QM*(4) = 0.21, *p* = .928 | 23/38 | *QM*(4) = 0.06, *p* = .992 | 12/18 | *QM*(2) = 0.40, *p* = .684 |
| ***Guidance*** |  |  |  |  |  |  |
| Unguided |  |  |  | 0.14 [0.06, 0.21], *p* < .001 |  |  |
| Guided |  |  |  | 0.35 [0.17, 0.53], *p* < .001 |  |  |
| Omnibus moderator test | 38/64 | *QM*(1) = 1.13, *p* = .295 | 36/57 | *QM*(1) = 5.21, *p* = .029* | 18/25 | *QM*(1) = 0.10, *p* = .756 |
| ***Intervention type*** (standalone vs. blended interventions) | | | | | | |
| Omnibus moderator test | 37/63 | *QM*(1) = 0.47, *p* = .498 | 35/56 | *QM*(1) = 0.01, *p* = .941 | 19/26 | *QM*(1) = 1.37, *p* = .258 |
| ***Degree of individualization*** (individualized vs. standardized) | | | | | | |
| Standardized |  |  |  |  |  | 0.22 [0.10, 0.33], *p* = .001 |
| Individualized |  |  |  |  |  | 0.03 [–0.05, 0.12], *p* = .423 |
| Omnibus moderator test | 37/60 | *QM*(1) = 0.12, *p* = .734 | 35/55 | *QM*(1) = 0.27, *p* = .605 | 18/25 | *QM*(1) = 6.60, *p* = .021* |
| ***Intervention intensity*** |  |  |  |  |  |  |
| in weeks | 36/60 | *QM*(1) = 0.26, *p* = .614 | 36/57 | *QM*(1) = 0.17, *p* = .686 | 18/25 | *QM*(1) = 0.03, *p* = .856 |
| ***Improvement over time*** |  |  |  |  |  |  |
| Publication year | 38/64 | *QM*(1) = 0.67, *p* = .419 | 36/57 | *QM*(1) = 1.71, *p* = .199 | 18/25 | *QM*(1) = 0.30, *p* = .591 |
| ***Type of control group*** |  |  |  |  |  |  |
| No intervention/ waitlist |  |  |  | 0.25 [0.13, 0.37], *p* < .001 |  |  |
| Low-intensity control |  |  |  | 0.19 [–0.03, 0.41], *p* = .081 |  |  |
| High-intensity control |  |  |  | 0.08 [0.01, 0.16], *p* = .029 |  |  |
| Omnibus moderator test | 38/64 | *QM*(2) = 1.04, *p* = .365 | 36/57 | *QM*(2) = 2.94, *p* = .067 | 18/25 | *QM*(2) = 0.20, *p* = .821 |
| ***COVID-19 context*** (before COVID-19 vs. during COVID-19) | | | | | | |
| Omnibus moderator test | 38/64 | *QM*(1) = 0.44, *p* = .510 | 36/57 | *QM*(1) = 1.20, *p* = .281 | 18/25 | *QM*(1) = 0.01, *p* = .944 |
| ***Small digital component*** (small e/mHealth component vs. other) | | | | | |  |
| Omnibus moderator test | 37/63 | *QM*(1) = 0.05, *p* = .827 | 35/56 | *QM*(1) = 0.90, *p* = .350 | 18/25 | *QM*(1) = 0.41, *p* = .533 |

*Note. Q_M_*(df) *=* omnibus test for moderators, which follows approximately a χ^2^ distribution (with associated degrees of freedom); *k* = number of effect sizes; SMD = standardized mean difference; *p = p* value; 95% CI = 95% confidence interval.

As results were at high risk to be biased by single studies, we do not report on moderation tests when three or less effect sizes were available per subgroup. In these cases, we only present subgroup results for the level with at least three effect sizes.

### Supplementary Data 16. Sensitivity analyses – part I: Other between-outcome correlations

**Table 11** Results of main analyses for primary outcomes comparing digital resilience interventions with comparators at post-intervention (A) and follow-up (B) – Other between-outcome correlations (*ρ* = .40)

| **Analysis** | ***n*** | ***k*** | ***M*(SMD)** | **95% CI** | **95% PI** | ***p*** | ***Q*** | ***df*** | ***p(Q)*** |
| --- | --- | --- | --- | --- | --- | --- | --- | --- | --- |
| ***A) Post-intervention assessment*** | |  |  |  |  |  |  |  |  |
| Mental distress | 85 | 150 | –0.24 | [–0.31, –0.18] | [–0.71, 0.23] | < .001 | 362.33 | 149 | < .001 |
| Positive mental health | 77 | 123 | 0.27 | [0.13, 0.40] | [–0.85, 1.39] | < .001 | 835.32 | 122 | < .001 |
| Resilience factors | 46 | 65 | 0.31 | [0.21, 0.41] | [–0.22, 0.83] | < .001 | 214.03 | 63 | < .001 |
|  |  |  |  |  |  |  |  |  |  |
| ***B) Follow-up assessment*** | | |  |  |  |  |  |  |  |
| Mental distress | 38 | 64 | –0.24 | [–0.35, –0.13] | [–0.82, 0.35] | < .001 | 204.32 | 63 | < .001 |
| Positive mental health | 36 | 57 | 0.19 | [0.11, 0.26] | [–0.19, 0.56] | < .001 | 121.94 | 56 | < .001 |
| Resilience factors | 18 | 25 | 0.18 | [0.07, 0.29] | [–0.25, 0.62] | .003 | 68.69 | 24 | < .001 |

*Note.* The multilevel meta-analysis on distress indicators included anxiety symptoms, depressive symptoms, general distress, PTSD symptoms, and stress symptoms. Due to qualitative differences, positive mental health and resilience factors were analyzed separately. Positive mental health comprised measures of happiness, life satisfaction, mental health, positive emotions/affect, stress-related/posttraumatic growth, quality of life, resilience, vitality, and wellbeing. Resilience factors comprised active coping, mindfulness, optimism, self-compassion, self-efficacy, self-esteem, and social support. For distress indicators, negative SMDs indicate favorable effects of an intervention [i.e., lower distress in the digital resilience intervention group compared to the control group]. For positive mental health and resilience factors, positive SMDs indicate favorable intervention effects [i.e., higher levels of positive mental health and resilience factors in the digital resilience intervention group compared to the control group]. All tests and reported statistics use cluster-robust estimates to account for non-independent effect estimates within studies. df = degrees of freedom; *k* = number of effect estimates; *n* = number of studies; Q = Cochran’s *Q* statistic with *p* value; SMD = standardized mean difference; 95% CI = 95% confidence interval; 95% PI = 95% prediction interval

**Table 12** Results of main analyses for primary outcomes comparing digital resilience interventions with comparators at post-intervention (A) and follow-up (B) – Other between-outcome correlations (*ρ* = .80)

| **Analysis** | ***n*** | ***k*** | ***M*(SMD)** | **95% CI** | **95% PI** | ***p*** | ***Q*** | ***df*** | ***p(Q)*** |
| --- | --- | --- | --- | --- | --- | --- | --- | --- | --- |
| ***A) Post-intervention assessment*** | |  |  |  |  |  |  |  |  |
| Mental distress | 85 | 150 | –0.24 | [–0.31, –0.18] | [–0.70, 0.21] | < .001 | 447.22 | 149 | < .001 |
| Positive mental health | 77 | 123 | 0.25 | [0.15, 0.35] | [–0.54, 1.05] | < .001 | 589.46 | 122 | < .001 |
| Resilience factors | 46 | 65 | 0.25 | [0.18, 0.32] | [–0.19, 0.68] | < .001 | 228.71 | 64 | < .001 |
|  |  |  |  |  |  |  |  |  |  |
| ***B) Follow-up assessment*** | | |  |  |  |  |  |  |  |
| Mental distress | 38 | 64 | –0.24 | [–0.35, –0.13] | [–0.83, 0.35] | < .001 | 230.21 | 63 | < .001 |
| Positive mental health | 36 | 57 | 0.19 | [0.11, 0.27] | [–0.22, 0.59] | < .001 | 206.16 | 56 | < .001 |
| Resilience factors | 18 | 25 | 0.19 | [0.08, 0.30] | [–0.26, 0.63] | .002 | 132.92 | 24 | < .001 |

*Note.* The multilevel meta-analysis on distress indicators included anxiety symptoms, depressive symptoms, general distress, PTSD symptoms, and stress symptoms. Due to qualitative differences, positive mental health and resilience factors were analyzed separately. Positive mental health comprised measures of happiness, life satisfaction, mental health, positive emotions/affect, stress-related/posttraumatic growth, quality of life, resilience, vitality, and wellbeing. Resilience factors comprised active coping, mindfulness, optimism, self-compassion, self-efficacy, self-esteem, and social support. For distress indicators, negative SMDs indicate favorable effects of an intervention [i.e., lower distress in the digital resilience intervention group compared to the control group]. For positive mental health and resilience factors, positive SMDs indicate favorable intervention effects [i.e., higher levels of positive mental health and resilience factors in the digital resilience intervention group compared to the control group]. All tests and reported statistics use cluster-robust estimates to account for non-independent effect estimates within studies. df = degrees of freedom; *k* = number of effect estimates; *n* = number of studies; Q = Cochran’s *Q* statistic with *p* value; SMD = standardized mean difference; 95% CI = 95% confidence interval; 95% PI = 95% prediction interval

### Supplementary Data 17. Sensitivity analyses – part II: Risk of bias

**Table 13** Results of main analyses for primary outcomes comparing digital resilience interventions with comparators at post-intervention (A) and follow-up (B) – Randomization process

| **Analysis** | ***n*** | ***k*** | ***M*(SMD)** | **95% CI** | **95% PI** | ***p*** | ***Q*** | ***df*** | ***p(Q)*** |
| --- | --- | --- | --- | --- | --- | --- | --- | --- | --- |
| ***A) Post-intervention assessment*** | |  |  |  |  |  |  |  |  |
| Mental distress | 35 | 65 | –0.24 | [–0.33, –0.15] | [–0.70, 0.22] | < .001 | 195.21 | 64 | < .001 |
| Positive mental health | 33 | 52 | 0.25 | [0.17, 0.32] | [–0.06, 0.55] | < .001 | 117.31 | 51 | < .001 |
| Resilience factors | 13 | 16 | 0.16 | [–0.01, 0.34] | [–0.42, 0.75] | .066 | 68.97 | 15 | < .001 |
|  |  |  |  |  |  |  |  |  |  |
| ***B) Follow-up assessment*** | | |  |  |  |  |  |  |  |
| Mental distress | 19 | 32 | –0.21 | [–0.33, –0.10] | [–0.62, 0.19] | .001 | 63.38 | 31 | < .001 |
| Positive mental health | 25 | 39 | 0.20 | [0.11, 0.30] | [–0.20, 0.61] | < .001 | 101.24 | 38 | < .001 |
| Resilience factors | 9 | 13 | 0.16 | [0.02, 0.30] | [–0.31, 0.63] | .030 | 82.57 | 12 | < .001 |

*Note.* The multilevel meta-analysis on distress indicators included anxiety symptoms, depressive symptoms, general distress, PTSD symptoms, and stress symptoms. Due to qualitative differences, positive mental health and resilience factors were analyzed separately. Positive mental health comprised measures of happiness, life satisfaction, mental health, positive emotions/affect, stress-related/posttraumatic growth, quality of life, resilience, vitality, and wellbeing. Resilience factors comprised active coping, mindfulness, optimism, self-compassion, self-efficacy, self-esteem, and social support. For distress indicators, negative SMDs indicate favorable effects of an intervention [i.e., lower distress in the digital resilience intervention group compared to the control group]. For positive mental health and resilience factors, positive SMDs indicate favorable intervention effects [i.e., higher levels of positive mental health and resilience factors in the digital resilience intervention group compared to the control group]. All tests and reported statistics use cluster-robust estimates to account for non-independent effect estimates within studies. df = degrees of freedom; *k* = number of effect estimates; *n* = number of studies; Q = Cochran’s *Q* statistic with *p* value; SMD = standardized mean difference; 95% CI = 95% confidence interval; 95% PI = 95% prediction interval.

**Table 14** Results of main analyses for primary outcomes comparing digital resilience interventions with comparators at post-intervention (A) and follow-up (B) – Deviations from intended interventions

| **Analysis** | ***n*** | ***k*** | ***M*(SMD)** | **95% CI** | **95% PI** | ***p*** | ***Q*** | ***df*** | ***p(Q)*** |
| --- | --- | --- | --- | --- | --- | --- | --- | --- | --- |
| ***A) Post-intervention assessment*** | |  |  |  |  |  |  |  |  |
| Mental distress | 35 | 65 | –0.24 | [–0.33, –0.15] | [–0.70, 0.22] | < .001 | 195.21 | 64 | < .001 |
| Positive mental health | 58 | 92 | 0.31 | [0.12, 0.50] | [–1.07, 1.70] | .002 | 1472.35 | 91 | < .001 |
| Resilience factors | 36 | 49 | 0.33 | [0.21, 0.45] | [–0.23, 0.89] | < .001 | 181.35 | 48 | < .001 |
|  |  |  |  |  |  |  |  |  |  |
| ***B) Follow-up assessment*** | | |  |  |  |  |  |  |  |
| Mental distress | 27 | 44 | –0.30 | [–0.43, –0.16] | [–0.93, 0.34] | < .001 | 163.44 | 43 | < .001 |
| Positive mental health | 17 | 25 | 0.19 | [0.03, 0.36] | [–0.45, 0.84] | .028 | 95.28 | 24 | < .001 |
| Resilience factors | 12 | 17 | 0.23 | [0.10, 0.35] | [–0.10, 0.55] | .002 | 57.24 | 16 | < .001 |

*Note.* The multilevel meta-analysis on distress indicators included anxiety symptoms, depressive symptoms, general distress, PTSD symptoms, and stress symptoms. Due to qualitative differences, positive mental health and resilience factors were analyzed separately. Positive mental health comprised measures of happiness, life satisfaction, mental health, positive emotions/affect, stress-related/posttraumatic growth, quality of life, resilience, vitality, and wellbeing. Resilience factors comprised active coping, mindfulness, optimism, self-compassion, self-efficacy, self-esteem, and social support. For distress indicators, negative SMDs indicate favorable effects of an intervention [i.e., lower distress in the digital resilience intervention group compared to the control group]. For positive mental health and resilience factors, positive SMDs indicate favorable intervention effects [i.e., higher levels of positive mental health and resilience factors in the digital resilience intervention group compared to the control group]. All tests and reported statistics use cluster-robust estimates to account for non-independent effect estimates within studies. df = degrees of freedom; *k* = number of effect estimates; *n* = number of studies; Q = Cochran’s *Q* statistic with *p* value; SMD = standardized mean difference; 95% CI = 95% confidence interval; 95% PI = 95% prediction interval.

**Table 15** Results of main analyses for primary outcomes comparing digital resilience interventions with comparators at post-intervention (A) and follow-up (B) – Missing outcome data

| **Analysis** | ***n*** | ***k*** | ***M*(SMD)** | **95% CI** | **95% PI** | ***p*** | ***Q*** | ***df*** | ***p(Q)*** |
| --- | --- | --- | --- | --- | --- | --- | --- | --- | --- |
| ***A) Post-intervention assessment*** | |  |  |  |  |  |  |  |  |
| Mental distress | 41 | 72 | –0.23 | [–0.33, –0.13] | [–0.73, 0.27] | < .001 | 176.91 | 71 | < .001 |
| Positive mental health | 40 | 61 | 0.30 | [0.04, 0.56] | [–1.32, 1.92] | .027 | 1213.67 | 60 | < .001 |
| Resilience factors | 25 | 32 | 0.22 | [0.11, 0.33] | [–0.20, 0.65] | < .001 | 82.28 | 31 | < .001 |
|  |  |  |  |  |  |  |  |  |  |
| ***B) Follow-up assessment*** | | |  |  |  |  |  |  |  |
| Mental distress | 17 | 27 | –0.26 | [–0.41, –0.10] | [–0.79, 0.28] | .003 | 67.65 | 26 | < .001 |
| Positive mental health | 17 | 25 | 0.19 | [0.03, 0.36] | [–0.45, 0.84] | .024 | 95.28 | 24 | < .001 |
| Resilience factors | 11 | 14 | 0.22 | [0.03, 0.40] | [–0.38, 0.81] | .027 | 87.42 | 13 | < .001 |

*Note.* The multilevel meta-analysis on distress indicators included anxiety symptoms, depressive symptoms, general distress, PTSD symptoms, and stress symptoms. Due to qualitative differences, positive mental health and resilience factors were analyzed separately. Positive mental health comprised measures of happiness, life satisfaction, mental health, positive emotions/affect, stress-related/posttraumatic growth, quality of life, resilience, vitality, and wellbeing. Resilience factors comprised active coping, mindfulness, optimism, self-compassion, self-efficacy, self-esteem, and social support. For distress indicators, negative SMDs indicate favorable effects of an intervention [i.e., lower distress in the digital resilience intervention group compared to the control group]. For positive mental health and resilience factors, positive SMDs indicate favorable intervention effects [i.e., higher levels of positive mental health and resilience factors in the digital resilience intervention group compared to the control group]. All tests and reported statistics use cluster-robust estimates to account for non-independent effect estimates within studies. df = degrees of freedom; *k* = number of effect estimates; *n* = number of studies; Q = Cochran’s *Q* statistic with *p* value; SMD = standardized mean difference; 95% CI = 95% confidence interval; 95% PI = 95% prediction interval

**Table 16** Results of main analyses for primary outcomes comparing digital resilience interventions with comparators at post-intervention (A) and follow-up (B) – Measurement of outcome

| **Analysis** | ***n*** | ***k*** | ***M*(SMD)** | **95% CI** | **95% PI** | ***p*** | ***Q*** | ***df*** | ***p(Q)*** |
| --- | --- | --- | --- | --- | --- | --- | --- | --- | --- |
| ***A) Post-intervention assessment*** | |  |  |  |  |  |  |  |  |
| Mental distress | 30 | 50 | –0.14 | [–0.23, –0.05] | [–0.46, 0.18] | .003 | 66.15 | 49 | .052 |
| Positive mental health | 27 | 36 | 0.06 | [–0.03, 0.15] | [–0.28, 0.41] | .171 | 71.94 | 35 | < .001 |
| Resilience factors | 11 | 13 | 0.20 | [0.01, 0.39] | [–0.28, 0.69] | .037 | 26.77 | 12 | .008 |
|  |  |  |  |  |  |  |  |  |  |
| ***B) Follow-up assessment*** | | |  |  |  |  |  |  |  |
| Mental distress | 20 | 34 | –0.16 | [–0.31, –0.01] | [–0.71, 0.40] | .040 | 87.52 | 33 | < .001 |
| Positive mental health | 20 | 30 | 0.09 | [0.03, 0.16] | [–0.26, 0.44] | .006 | 62.90 | 29 | < .001 |
| Resilience factors | 9 | 11 | 0.14 | [–0.07, 0.36] | [–0.48, 0.77] | .162 | 70.93 | 10 | < .001 |

*Note.* The multilevel meta-analysis on distress indicators included anxiety symptoms, depressive symptoms, general distress, PTSD symptoms, and stress symptoms. Due to qualitative differences, positive mental health and resilience factors were analyzed separately. Positive mental health comprised measures of happiness, life satisfaction, mental health, positive emotions/affect, stress-related/posttraumatic growth, quality of life, resilience, vitality, and wellbeing. Resilience factors comprised active coping, mindfulness, optimism, self-compassion, self-efficacy, self-esteem, and social support. For distress indicators, negative SMDs indicate favorable effects of an intervention [i.e., lower distress in the digital resilience intervention group compared to the control group]. For positive mental health and resilience factors, positive SMDs indicate favorable intervention effects [i.e., higher levels of positive mental health and resilience factors in the digital resilience intervention group compared to the control group]. All tests and reported statistics use cluster-robust estimates to account for non-independent effect estimates within studies. df = degrees of freedom; *k* = number of effect estimates; *n* = number of studies; Q = Cochran’s *Q* statistic with *p* value; SMD = standardized mean difference; 95% CI = 95% confidence interval; 95% PI = 95% prediction interval.

**Table 17** Results of main analyses for primary outcomes comparing digital resilience interventions with comparators at post-intervention (A) and follow-up (B) – Selection of reported results

| **Analysis** | ***n*** | ***k*** | ***M*(SMD)** | **95% CI** | **95% PI** | ***p*** | ***Q*** | ***df*** | ***p(Q)*** |
| --- | --- | --- | --- | --- | --- | --- | --- | --- | --- |
| ***A) Post-intervention assessment*** | |  |  |  |  |  |  |  |  |
| Mental distress | 34 | 71 | –0.25 | [–0.34, –0.16] | [–0.67, 0.17] | < .001 | 163.11 | 70 | < .001 |
| Positive mental health | 28 | 46 | 0.23 | [0.15, 0.30] | [–0.06, 0.51] | < .001 | 98.27 | 45 | < .001 |
| Resilience factors | 16 | 25 | 0.22 | [0.08, 0.36] | [–0.26, 0.71[ | .004 | 97.24 | 24 | < .001 |
|  |  |  |  |  |  |  |  |  |  |
| ***B) Follow-up assessment*** | | |  |  |  |  |  |  |  |
| Mental distress | 20 | 39 | –0.33 | [–0.49, –0.17] | [–0.98, 0.31] | < .001 | 106.06 | 38 | < .001 |
| Positive mental health | 17 | 26 | 0.19 | [0.09, 0.29] | [–0.21, 0.59] | < .001 | 68.63 | 25 | < .001 |
| Resilience factors | 8 | 13 | 0.20 | [0.09, 0.32] | [–0.16, 0.56] | .004 | 65.56 | 12 | < .001 |

*Note.* The multilevel meta-analysis on distress indicators included anxiety symptoms, depressive symptoms, general distress, PTSD symptoms, and stress symptoms. Due to qualitative differences, positive mental health and resilience factors were analyzed separately. Positive mental health comprised measures of happiness, life satisfaction, mental health, positive emotions/affect, stress-related/posttraumatic growth, quality of life, resilience, vitality, and wellbeing. Resilience factors comprised active coping, mindfulness, optimism, self-compassion, self-efficacy, self-esteem, and social support. For distress indicators, negative SMDs indicate favorable effects of an intervention [i.e., lower distress in the digital resilience intervention group compared to the control group]. For positive mental health and resilience factors, positive SMDs indicate favorable intervention effects [i.e., higher levels of positive mental health and resilience factors in the digital resilience intervention group compared to the control group]. All tests and reported statistics use cluster-robust estimates to account for non-independent effect estimates within studies. df = degrees of freedom; *k* = number of effect estimates; *n* = number of studies; Q = Cochran’s *Q* statistic with *p* value; SMD = standardized mean difference; 95% CI = 95% confidence interval; 95% PI = 95% prediction interval.

### Supplementary Data 18. References of studies included in this review

Abbott, J. A., Klein, B., Hamilton, C., & Rosenthal, A. J. (2009). The impact of online resilience training for sales managers on wellbeing and performance. Sensoria: *A Journal of Mind, Brain & Culture, 5*(1), 89–95.

Aboody, D., Siev, J., & Doron, G. (2020). Building resilience to body image triggers using brief cognitive training on a mobile application: A randomized controlled trial. *Behaviour research and therapy*, *134*, 103723. https://doi.org/10.1016/j.brat.2020.103723

Aikens, K. A., Astin, J., Pelletier, K. R., Levanovich, K., Baase, C. M., Park, Y. Y., & Bodnar, C. M. (2014). Mindfulness goes to work: impact of an online workplace intervention. *Journal of occupational and environmental medicine, 56*(7), 721–731. https://doi.org/10.1097/JOM.0000000000000209

Al-Refae, M., Al-Refae, A., Munroe, M., Sardella, N. A., & Ferrari, M. (2021). A Self-Compassion and Mindfulness-Based Cognitive Mobile Intervention (Serene) for Depression, Anxiety, and Stress: Promoting Adaptive Emotional Regulation and Wisdom. *Frontiers in psychology*, *12*, 648087. https://doi.org/10.3389/fpsyg.2021.648087

Aminoff, V., Sellén, M., Sörliden, E., Ludvigsson, M., Berg, M., & Andersson, G. (2021). Internet-Based Cognitive Behavioral Therapy for Psychological Distress Associated With the COVID-19 Pandemic: A Pilot Randomized Controlled Trial. *Frontiers in psychology*, *12*, 684540. https://doi.org/10.3389/fpsyg.2021.684540

Atad, O. I., & Grant, A. M. (2021). Evidence-based coaching as a supplement to traditional lectures: impact on undergraduates' goal attainment and measures of mental well-being. *International Journal of Mentoring and Coaching in Education, 10*(3). 249–266. https://doi.org/10.1108/IJMCE-05-2020-0024

Auyeung, L., & Mo, P. K. H. (2019). The efficacy and mechanism of online positive psychological intervention (PPI) on improving well-being among Chinese university students: A pilot study of the best possible self (BPS) intervention. *Journal of Happiness Studies*, *20*(8), 2525–2550.

Ayers, S., Fitzgerald, G., & Thompson, S. (2015). Brief online self-help exercises for postnatal women to improve mood: a pilot study. *Maternal and child health journal*, *19*, 2375–2383. https://doi.org/10.1007/s10995-015-1755-5

Bani Ahmad, T., & Meriç, M. (2021). The effect of an online psychoeducational stress management program on international students' ability to cope and adapt. *Perspectives in psychiatric care*, *57*(4), 1673–1684. https://doi.org/10.1111/ppc.12735

Behrendt, D., Boß, L., Hannibal, S., Kunzler, A. M., Wessa, M., & Lehr, D. (2023). Feasibility and efficacy of a digital resilience training: A pilot study of the strengths-based training RESIST. *Internet Interventions*, 100649.

Ben-Avraham, R., Afek, A., Berezin Cohen, N., Davidov, A., Van Vleet, T., Jordan, J., Ben Yehudah, A., Gilboa, Y., & Nahum, M. (2021). Feasibility and preliminary effectiveness of mobile cognitive control training during basic combat training in the military. *Military Psychology*, *34*(1), 55–67. https://doi.org/10.1080/08995605.2021.1969162

Beyer, J. A. (2010). *The effects of web-based interactive emotional disclosure on stress and health: A randomized, controlled study*. Dissertation, Wayne State University.

Birk, M. V., & Mandryk, R. L. (2019). Improving the efficacy of cognitive training for digital mental health interventions through avatar customization: crowdsourced quasi-experimental study. *Journal of medical Internet research*, *21*(1), e10133. https://doi.org/10.2196/10133

Bolier, L., Ketelaar, S. M., Nieuwenhuijsen, K., Smeets, O., Gärtner, F. R., & Sluiter, J. K. (2014). Workplace mental health promotion online to enhance well-being of nurses and allied health professionals: A cluster-randomized controlled trial. *Internet interventions, 1*(4), 196-204. https://doi.org/10.1016/j.invent.2014.10.002

Bouchard, S., Bernier, F., Boivin, E., Morin, B., & Robillard, G. (2012). Using biofeedback while immersed in a stressful videogame increases the effectiveness of stress management skills in soldiers. *PloS one*, *7*(4), e36169. https://doi.org/10.1371/journal.pone.0036169

Brog, N. A., Hegy, J. K., Berger, T., & Znoj, H. (2022). Effects of an internet-based self-help intervention for psychological distress due to COVID-19: Results of a randomized controlled trial. *Internet interventions*, *27*, 100492. https://doi.org/10.1016/j.invent.2021.100492

Burger, K. G. (2015). *Examining the use of mindfulness meditation to enhance attention regulation efficiency in nursing students*. Dissertation, Duquesne University.

Catuara-Solarz, S., Skorulski, B., Estella-Aguerri, I., Avella-Garcia, C. B., Shepherd, S., Stott, E., Hemmings, N. R., Ruiz de Villa, A., Schulze, L., & Dix, S. (2022). The efficacy of "foundations," a digital mental health app to improve mental well-being during COVID-19: proof-of-principle randomized controlled trial. *JMIR mHealth and uHealth, 10*(7), e30976. https://doi.org/10.2196/30976

Cavanagh, K., Churchard, A., O'Hanlon, P., Mundy, T., Votolato, P., Jones, F., Gu, J., & Strauss, C. (2018). A Randomised Controlled Trial of a Brief Online Mindfulness-Based Intervention in a Non-clinical Population: Replication and Extension. *Mindfulness*, *9*(4), 1191–1205. https://doi.org/10.1007/s12671-017-0856-1

Champion, L., Economides, M., & Chandler, C. (2018). The efficacy of a brief app-based mindfulness intervention on psychosocial outcomes in healthy adults: A pilot randomised controlled trial. *PloS one*, *13*(12), e0209482. https://doi.org/10.1371/journal.pone.0209482

Chang, T. F. H., Ley, B. L., Ramburn, T. T., Srinivasan, S., Hariri, S., Purandare, P., & Subramaniam, B. (2022). Online Isha Upa Yoga for student mental health and well-being during COVID-19: A randomized control trial. *Applied psychology. Health and well-being*, *14*(4), 1408–1428. https://doi.org/10.1111/aphw.12341

Chilver, M. R., & Gatt, J. M. (2022). Six-Week Online Multi-component Positive Psychology Intervention Improves Subjective Wellbeing in Young Adults. *Journal of happiness studies*, *23*(3), 1267–1288. https://doi.org/10.1007/s10902-021-00449-3

Cieslak, R., Benight, C. C., Rogala, A., Smoktunowicz, E., Kowalska, M., Zukowska, K., Yeager, C., & Luszczynska, A. (2016). Effects of Internet-Based Self-Efficacy Intervention on Secondary Traumatic Stress and Secondary Posttraumatic Growth among Health and Human Services Professionals Exposed to Indirect Trauma. *Frontiers in psychology*, *7*, 1009. https://doi.org/10.3389/fpsyg.2016.01009

Decker, M. R., Wood, S. N., Hameeduddin, Z., Kennedy, S. R., Perrin, N., Tallam, C., Akumu, I., Wanjiru, I., Asira, B., Frankel, A., Omondi, B., Case, J., Clough, A., Otieno, R., Mwiti, M., & Glass, N. (2020). Safety decision-making and planning mobile app for intimate partner violence prevention and response: randomised controlled trial in Kenya. *BMJ global health*, *5*(7), e002091. https://doi.org/10.1136/bmjgh-2019-002091

Ebert, D. D., Franke, M., Zarski, A. C., Berking, M., Riper, H., Cuijpers, P., Funk, B., & Lehr, D. (2021). Effectiveness and Moderators of an Internet-Based Mobile-Supported Stress Management Intervention as a Universal Prevention Approach: Randomized Controlled Trial. *Journal of medical Internet research*, *23*(12), e22107. https://doi.org/10.2196/22107

Enrique, Á., Bretón-López, J., Molinari, G., Baños, R. M., & Botella, C. (2018). Efficacy of an adaptation of the Best Possible Self intervention implemented through positive technology: a randomized control trial. *Applied Research in Quality of Life*, *13*, 671–689. https://doi.org/10.1007/s11482-017-9552-5

Eriksson, T., Germundsjö, L., Åström, E., & Rönnlund, M. (2018). Mindful Self-Compassion Training Reduces Stress and Burnout Symptoms Among Practicing Psychologists: A Randomized Controlled Trial of a Brief Web-Based Intervention. *Frontiers in psychology*, *9*, 2340. https://doi.org/10.3389/fpsyg.2018.02340

Fassnacht, D. B., Ali, K., van Agteren, J., Iasiello, M., Mavrangelos, T., Furber, G., & Kyrios, M. (2022). A Group-Facilitated, Internet-Based Intervention to Promote Mental Health and Well-Being in a Vulnerable Population of University Students: Randomized Controlled Trial of the Be Well Plan Program. *JMIR mental health*, *9*(5), e37292. https://doi.org/10.2196/37292

Feinberg, M. E., Boring, J., Le, Y., Hostetler, M. L., Karre, J., Irvin, J., & Jones, D. E. (2020). Supporting military family resilience at the transition to parenthood: A randomized pilot trial of an online version of family foundations. *Family Relations: An Interdisciplinary Journal of Applied Family Studies, 69*(1), 109–124. https://doi.org/10.1111/fare.12415

Flett, J. A. M., Conner, T. S., Riordan, B. C., Patterson, T., & Hayne, H. (2020). App-based mindfulness meditation for psychological distress and adjustment to college in incoming university students: a pragmatic, randomised, waitlist-controlled trial. *Psychology & health*, *35*(9), 1049–1074. https://doi.org/10.1080/08870446.2019.1711089

Flett, J. A. M., Hayne, H., Riordan, B. C., Thompson, L. M., & Conner, T. S. (2019). Mobile mindfulness meditation: A randomised controlled trial of the effect of two popular apps on mental health. *Mindfulness, 10*(5), 863–876. https://doi.org/10.1007/s12671-018-1050-9

Gollwitzer, P. M., Mayer, D., Frick, C., & Oettingen, G. (2018). Promoting the Self-Regulation of Stress in Health Care Providers: An Internet-Based Intervention. *Frontiers in psychology*, *9*, 838. https://doi.org/10.3389/fpsyg.2018.00838

Grabbe, L., Higgins, M. K., Baird, M., Craven, P. A., & San Fratello, S. (2020). The Community Resiliency Model¬Æ to promote nurse well-being. Nursing outlook, 68(3), 324-336.

Hannibal, S., Behrendt, D., Lehr, D. (2022). Unpublished data on a guided web- and app-based resilience intervention.

Harrer, M., Adam, S. H., Fleischmann, R. J., Baumeister, H., Auerbach, R., Bruffaerts, R., Cuijpers, P., Kessler, R. C., Berking, M., Lehr, D., & Ebert, D. D. (2018). Effectiveness of an Internet- and App-Based Intervention for College Students With Elevated Stress: Randomized Controlled Trial. *Journal of medical Internet research*, *20*(4), e136. https://doi.org/10.2196/jmir.9293

Harrer, M., Apolinário-Hagen, J., Fritsche, L., Salewski, C., Zarski, A. C., Lehr, D., Baumeister, H., Cuijpers, P., & Ebert, D. D. (2021). Effect of an internet- and app-based stress intervention compared to online psychoeducation in university students with depressive symptoms: Results of a randomized controlled trial. *Internet interventions*, *24*, 100374. https://doi.org/10.1016/j.invent.2021.100374

Heckendorf, H., Lehr, D., & Boß, L. (2022). Effectiveness of an Internet-Based Self-Help Intervention versus Public Mental Health Advice to Reduce Worry during the COVID-19 Pandemic: A Pragmatic, Parallel-Group, Randomized Controlled Trial. *Psychotherapy and psychosomatics*, *91*(6), 398–410. https://doi.org/10.1159/000521302

Heckendorf, H., Lehr, D., Ebert, D. D., & Freund, H. (2019). Efficacy of an internet and app-based gratitude intervention in reducing repetitive negative thinking and mechanisms of change in the intervention's effect on anxiety and depression: Results from a randomized controlled trial. *Behaviour research and therapy*, *119*, 103415. https://doi.org/10.1016/j.brat.2019.103415

Hersch, R. K., Cook, R. F., Deitz, D. K., Kaplan, S., Hughes, D., Friesen, M. A., & Vezina, M. (2016). Reducing nurses' stress: A randomized controlled trial of a web-based stress management program for nurses. *Applied nursing research : ANR*, *32*, 18–25. https://doi.org/10.1016/j.apnr.2016.04.003

Hoorelbeke, K., & Koster, E. H. W. (2017). Internet-delivered cognitive control training as a preventive intervention for remitted depressed patients: Evidence from a double-blind randomized controlled trial study. *Journal of consulting and clinical psychology*, *85*(2), 135–146. https://doi.org/10.1037/ccp0000128

Hoorelbeke, K., Koster, E. H., Vanderhasselt, M. A., Callewaert, S., & Demeyer, I. (2015). The influence of cognitive control training on stress reactivity and rumination in response to a lab stressor and naturalistic stress. *Behaviour research and therapy*, *69*, 1–10. https://doi.org/10.1016/j.brat.2015.03.010

Hsieh, H. F., Huang, I. C., Liu, Y., Chen, W. L., Lee, Y. W., & Hsu, H. T. (2020). The Effects of Biofeedback Training and Smartphone-Delivered Biofeedback Training on Resilience, Occupational Stress, and Depressive Symptoms among Abused Psychiatric Nurses. *International journal of environmental research and public health*, *17*(8), 2905. https://doi.org/10.3390/ijerph17082905

Jackson, A. C. (2019). *The efficacy of coaching interventions for undergraduates to increase positive coping behavior: a quantitative quasi-experiment*. Dissertation, Concordia University.

Kahn, J. R., Collinge, W., & Soltysik, R. (2016). Post-9/11 Veterans and Their Partners Improve Mental Health Outcomes with a Self-directed Mobile and Web-based Wellness Training Program: A Randomized Controlled Trial. *Journal of medical Internet research*, *18*(9), e255. https://doi.org/10.2196/jmir.5800

Kim, J. I., Yun, J. Y., Park, H., Park, S. Y., Ahn, Y., Lee, H., Kim, T. K., Yoon, S., Lee, Y. J., Oh, S., Denninger, J. W., Kim, B. N., & Kim, J. H. (2018). A Mobile Videoconference-Based Intervention on Stress Reduction and Resilience Enhancement in Employees: Randomized Controlled Trial. *Journal of medical Internet research*, *20*(10), e10760. https://doi.org/10.2196/10760

Kloos, N., Drossaert, C. H. C., Bohlmeijer, E. T., & Westerhof, G. J. (2019). Online positive psychology intervention for nursing home staff: A cluster-randomized controlled feasibility trial of effectiveness and acceptability. *International journal of nursing studies*, *98*, 48–56. https://doi.org/10.1016/j.ijnurstu.2019.06.004

Köhle, N., Drossaert, C. H. C., Ten Klooster, P. M., Schreurs, K. M. G., Hagedoorn, M., Van Uden-Kraan, C. F., Verdonck-de Leeuw, I. M., & Bohlmeijer, E. T. (2021). Web-based self-help intervention for partners of cancer patients based on acceptance and commitment therapy and self-compassion training: a randomized controlled trial with automated versus personal feedback. *Supportive care in cancer : official journal of the Multinational Association of Supportive Care in Cancer*, *29*(9), 5115–5125. https://doi.org/10.1007/s00520-021-06051-w

Koydemir, S., & Sun-Selışık, Z. E. (2016). Well-being on campus: Testing the effectiveness of an online strengths-based intervention for first year college students. *British Journal of Guidance & Counselling, 44*(4), 434–446. https://doi.org/10.1080/03069885.2015.1110562

Krifa, I., Hallez, Q., van Zyl, L. E., Braham, A., Sahli, J., Ben Nasr, S., & Shankland, R. (2022). Effectiveness of an online positive psychology intervention among Tunisian healthcare students on mental health and study engagement during the Covid-19 pandemic. *Applied psychology. Health and well-being*, *14*(4), 1228–1254. https://doi.org/10.1111/aphw.12332

Kubo, A., Kurtovich, E., McGinnis, M., Aghaee, S., Altschuler, A., Quesenberry, C., Jr, Kolevska, T., & Avins, A. L. (2019). A Randomized Controlled Trial of mHealth Mindfulness Intervention for Cancer Patients and Informal Cancer Caregivers: A Feasibility Study Within an Integrated Health Care Delivery System. *Integrative cancer therapies*, *18*, 1534735419850634. https://doi.org/10.1177/1534735419850634

Kuhlthau, K. A., Luberto, C. M., Traeger, L., Millstein, R. A., Perez, G. K., Lindly, O. J., Chad-Friedman, E., Proszynski, J., & Park, E. R. (2020). A Virtual Resiliency Intervention for Parents of Children with Autism: A Randomized Pilot Trial. *Journal of autism and developmental disorders*, *50*(7), 2513–2526. https://doi.org/10.1007/s10803-019-03976-4

Litvin, S., Saunders, R., Maier, M. A., & Lüttke, S. (2020). Gamification as an approach to improve resilience and reduce attrition in mobile mental health interventions: A randomized controlled trial. *PloS one*, *15*(9), e0237220. https://doi.org/10.1371/journal.pone.0237220

Liu, S. R., & Kia-Keating, M. (2018). Improving coping self-efficacy among distressed students after exposure to university mass violence: A pilot online intervention. *Journal of College Student Psychotherapy, 32*(3), 199–219. https://doi.org/10.1080/87568225.2017.1388754

Liu, S., Yuan, Q., Koon, S. F., Au, W. T., & Mak, W. S. (2016). ‘Happy@Work’: An innovative web-based program to promote positive mental well-being among working population - final report (project No.: 05120045). Available from https://rfs1.healthbureau.gov.hk/search/#/fundedsearch/projectdetail?id=1299&lang=en.

Luo, Y., Xia, W., Cheung, A. T., Ho, L. L. K., Zhang, J., Xie, J., Xiao, P., & Li, H. C. W. (2021). Effectiveness of a mobile device-based resilience training program in reducing depressive symptoms and enhancing resilience and quality of life in parents of children with cancer: randomized controlled trial. *Journal of medical Internet research*, *23*(11), e27639. https://doi.org/10.2196/27639

Luthans, F., Avey, J. B., & Patera, J. L. (2008). Experimental analysis of a web-based training intervention to develop positive psychological capital. *Academy of Management Learning & Education, 7*(2), 209–221. https://doi.org/10.5465/AMLE.2008.32712618

Mayor-Silva, L. I., Romero-Saldaña, M., Moreno-Pimentel, A. G., Álvarez-Melcón, Á., Molina-Luque, R., & Meneses-Monroy, A. (2021). The role of psychological variables in improving resilience: Comparison of an online intervention with a face-to-face intervention. A randomised controlled clinical trial in students of health sciences. *Nurse education today*, *99*, 104778. https://doi.org/10.1016/j.nedt.2021.104778

Medisauskaite, A., & Kamau, C. (2019). Reducing burnout and anxiety among doctors: Randomized controlled trial. *Psychiatry research*, *274*, 383–390. https://doi.org/10.1016/j.psychres.2019.02.075

Mistretta, E. G., Davis, M. C., Temkit, MH., Lorenz, C., Darby, B., & Stonnington, C. M. (2018). Resilience training for work-related stress among health care workers. *Journal of Occupational and Environmental Medicine*, *60*(6), 559-568. https://doi.org/10.1097/JOM.0000000000001285

Mogil, C., Hajal, N., Aralis, H., Paley, B., Milburn, N. G., Barrera, W., Kiff, C., Beardslee, W., & Lester, P. (2022). A trauma-informed, family-centered, virtual home visiting program for young children: one-year outcomes. *Child psychiatry and human development*, *53*(5), 964–979. https://doi.org/10.1007/s10578-021-01181-y

Mongrain, M., Komeylian, Z., & Barnhart, R. (2016). Happiness vs. mindfulness exercises for individuals vulnerable to depression. *The Journal of Positive Psychology, 11*(4), 366–377. https://doi.org/10.1080/17439760.2015.1092569

Morledge, T. J., Allexandre, D., Fox, E., Fu, A. Z., Higashi, M. K., Kruzikas, D. T., Pham, S. V., & Reese, P. R. (2013). Feasibility of an online mindfulness program for stress management--a randomized, controlled trial. *Annals of behavioral medicine : a publication of the Society of Behavioral Medicine*, *46*(2), 137–148. https://doi.org/10.1007/s12160-013-9490-x

Mueller, K., Prins, R., & de Heer, H. D. (2018). An Online Intervention Increases Empathy, Resilience, and Work Engagement Among Physical Therapy Students. *Journal of allied health*, *47*(3), 196–203.

Mulligan, K., Hirani, S. P., Harris, S., Taylor, J., Wedderburn, L. R., Newman, S., & WebParC Investigator group (2022). The Effects of a Web-Based Tool for Parents of Children With Juvenile Idiopathic Arthritis: Randomized Controlled Trial. *Journal of medical Internet research*, *24*(5), e29787. https://doi.org/10.2196/29787

Nadler, R., Carswell, J. J., & Minda, J. P. (2020). Online Mindfulness Training Increases Well-Being, Trait Emotional Intelligence, and Workplace Competency Ratings: A Randomized Waitlist-Controlled Trial. *Frontiers in psychology*, *11*, 255. https://doi.org/10.3389/fpsyg.2020.00255

Nichols, L. O., Martindale-Adams, J., Zuber, J., Graney, M., Burns, R., & Clark, C. (2015). Support for spouses of postdeployment service members. *Military behavioral health, 3*(2), 125-137.

Nielsen, E. G., & Minda, J. P. (2021). The mindful lawyer: investigating the effects of two online mindfulness programs on self-reported well-being in the legal profession. *Journal of occupational and environmental medicine*, *63*(12), e871–e882. https://doi.org/10.1097/JOM.0000000000002393

Nixon, P., Boß, L., Heber, E., Ebert, D. D., & Lehr, D. (2021). A three-armed randomised controlled trial investigating the comparative impact of guidance on the efficacy of a web-based stress management intervention and health impairing and promoting mechanisms of prevention. *BMC public health*, *21*(1), 1511. https://doi.org/10.1186/s12889-021-11504-2

Oehler, M., & Psouni, E. (2019). "Partner in Prime"? Effects of repeated mobile security priming on attachment security and perceived stress in daily life. *Attachment & human development*, *21*(6), 638–657. https://doi.org/10.1080/14616734.2018.1517811

Pandya S. P. (2021). Examining the effectiveness of whatsapp-based spiritual posts on mitigating stress and building resilience, maternal confidence and self-efficacy among mothers of children with ASD. *Journal of autism and developmental disorders*, *51*(5), 1479–1495. https://doi.org/10.1007/s10803-020-04633-x

Park, E. R., Perez, G. K., Millstein, R. A., Luberto, C. M., Traeger, L., Proszynski, J., Chad-Friedman, E., & Kuhlthau, K. A. (2020). A virtual resiliency intervention promoting resiliency for parents of children with learning and attentional disabilities: a randomized pilot trial. *Maternal and child health journal*, *24*(1), 39–53. https://doi.org/10.1007/s10995-019-02815-3

Pauls, N., Schlett, C., Soucek, R., Ziegler, M., & Frank, N. (2016). Resilienz durch Training personaler Ressourcen stärken: Evaluation einer web-basierten Achtsamkeitsintervention. *Gruppe. Interaktion. Organisation. Zeitschrift für Angewandte Organisationspsychologie*, *47*(2), 105–117. https://doi.org/10.1007/s11612-016-0315-9

Pogrebtsova, E., Craig, J., Chris, A., O'Shea, D., & González-Morales, M. G. (2018). Exploring daily affective changes in university students with a mindful positive reappraisal intervention: A daily diary randomized controlled trial. *Stress and health : journal of the International Society for the Investigation of Stress*, *34*(1), 46–58. https://doi.org/10.1002/smi.2759

Profit, J., Adair, K. C., Cui, X., Mitchell, B., Brandon, D., Tawfik, D. S., Rigdon, J., Gould, J. B., Lee, H. C., Timpson, W. L., McCaffrey, M. J., Davis, A. S., Pammi, M., Matthews, M., Stark, A. R., Papile, L. A., Thomas, E., Cotten, M., Khan, A., & Sexton, J. B. (2021). Randomized controlled trial of the "WISER" intervention to reduce healthcare worker burnout. *Journal of perinatology : official journal of the California Perinatal Association*, *41*(9), 2225–2234. https://doi.org/10.1038/s41372-021-01100-y

Proyer, R. T., Gander, F., Wellenzohn, S., & Ruch, W. (2014). Positive psychology interventions in people aged 50-79 years: long-term effects of placebo-controlled online interventions on well-being and depression. *Aging & mental health*, *18*(8), 997–1005. https://doi.org/10.1080/13607863.2014.899978

Puertas-Gonzalez, J. A., Mariño-Narvaez, C., Romero-Gonzalez, B., Sanchez-Perez, G. M., & Peralta-Ramirez, M. I. (2022). Online cognitive behavioural therapy as a psychological vaccine against stress during the COVID-19 pandemic in pregnant women: A randomised controlled trial. *Journal of psychiatric research*, *152*, 397–405. https://doi.org/10.1016/j.jpsychires.2022.07.016

Pyne, J. M., Constans, J. I., Nanney, J. T., Wiederhold, M. D., Gibson, D. P., Kimbrell, T., Kramer, T. L., Pitcock, J. A., Han, X., Williams, D. K., Chartrand, D., Gevirtz, R. N., Spira, J., Wiederhold, B. K., McCraty, R., & McCune, T. R. (2019). Heart Rate Variability and Cognitive Bias Feedback Interventions to Prevent Post-deployment PTSD: Results from a Randomized Controlled Trial. *Military medicine*, *184*(1-2), e124–e132. https://doi.org/10.1093/milmed/usy171

Rackoff, G. N., Fitzsimmons-Craft, E. E., Taylor, C. B., Eisenberg, D., Wilfley, D. E., & Newman, M. G. (2022). A Randomized Controlled Trial of Internet-Based Self-Help for Stress During the COVID-19 Pandemic. *The Journal of adolescent health : official publication of the Society for Adolescent Medicine*, *71*(2), 157–163. https://doi.org/10.1016/j.jadohealth.2022.01.227

Ramey, S. L., Perkhounkova, Y., Hein, M., Bohr, N. L., & Anderson, A. A. (2017). Testing a Resilience Training Program in Police Recruits: A Pilot Study. *Biological research for nursing*, *19*(4), 440–449. https://doi.org/10.1177/1099800417699879

Riello, M., Purgato, M., Bove, C., Tedeschi, F., MacTaggart, D., Barbui, C., & Rusconi, E. (2021). Effectiveness of self-help plus (SH+) in reducing anxiety and post-traumatic symptomatology among care home workers during the COVID-19 pandemic: a randomized controlled trial. *Royal Society open science*, *8*(11), 210219. https://doi.org/10.1098/rsos.210219

Roepke, A. M., Benson, L., Tsukayama, E., & Yaden, D. B. (2018). Prospective writing: Randomized controlled trial of an intervention for facilitating growth after adversity. *The Journal of Positive Psychology, 13*(6), 627–642. https://doi.org/10.1080/17439760.2017.1365161

Röhr, S., Jung, F. U., Pabst, A., Grochtdreis, T., Dams, J., Nagl, M., Renner, A., Hoffmann, R., König, H. H., Kersting, A., & Riedel-Heller, S. G. (2021). A Self-Help App for Syrian Refugees With Posttraumatic Stress (Sanadak): Randomized Controlled Trial. *JMIR mHealth and uHealth*, *9*(1), e24807. https://doi.org/10.2196/24807

Roig, A., Mooney, O., Salamanca-Sanabria, A., Lee, C. T., Farrell, S., & Richards, D. (2020). Assessing the Efficacy and Acceptability of a Web-Based Intervention for Resilience Among College Students: Pilot Randomized Controlled Trial. *JMIR formative research*, *4*(11), e20167. https://doi.org/10.2196/20167

Rose, R. D., Buckey, J. C., Jr., Zbozinek, T. D., Motivala, S. J., Glenn, D. E., Cartreine, J. A., & Craske, M. G. (2013). A randomized controlled trial of a self-guided, multimedia, stress management and resilience training program. *Behaviour Research and Therapy, 51*(2), 106–112. https://doi.org/10.1016/j.brat.2012.11.003

Rullo, J. E., Sood, R., Fokken, S. C., Sood, A., Frohmader, K. S., Croghan, I. T., Schroeder, D. R., & Faubion, S. S. (2021). Couples' Use of Online Stress Management and Resiliency Training for Sexual Health Concerns: A Randomized Controlled Trial. *Sexual medicine*, *9*(4), 100404. https://doi.org/10.1016/j.esxm.2021.100404

Sanchez-Lopez, A., De Raedt, R., van Put, J., & Koster, E. H. W. (2019). A novel process-based approach to improve resilience: Effects of computerized mouse-based (gaze)contingent attention training (MCAT) on reappraisal and rumination. *Behaviour research and therapy*, *118*, 110–120. https://doi.org/10.1016/j.brat.2019.04.005

Schotanus-Dijkstra, M., Pieterse, M. E., Drossaert, C. H. C., Walburg, J. A., & Bohlmeijer, E. T. (2019). Possible mechanisms in a multicomponent email guided positive psychology intervention to improve mental well-being, anxiety and depression: A multiple mediation model. *The Journal of Positive Psychology, 14*(2), 141–155. https://doi.org/10.1080/17439760.2017.1388430

Seear, K. H., & Vella-Brodrick, D. A. (2013). Efficacy of positive psychology interventions to increase well-being: Examining the role of dispositional mindfulness. *Social Indicators Research, 114*(3), 1125–1141. https://doi.org/10.1007/s11205-012-0193-7

Seligman, M. E., Schulman, P., & Tryon, A. M. (2007). Group prevention of depression and anxiety symptoms. *Behaviour research and therapy*, *45*(6), 1111–1126. https://doi.org/10.1016/j.brat.2006.09.010

Sim, W. H., Fernando, L. M. N., Jorm, A. F., Rapee, R. M., Lawrence, K. A., Mackinnon, A. J., & Yap, M. B. H. (2020). A tailored online intervention to improve parenting risk and protective factors for child anxiety and depression: Medium-term findings from a randomized controlled trial. *Journal of affective disorders*, *277*, 814–824. https://doi.org/10.1016/j.jad.2020.09.019

Spilg, E. G., Kuk, H., Ananny, L., McNeill, K., LeBlanc, V., Bauer, B. A., Sood, A., & Wells, P. S. (2022). The impact of Stress Management and Resailience Training (SMART) on academic physicians during the implementation of a new Health Information System: An exploratory randomized controlled trial. *PloS one*, *17*(4), e0267240. https://doi.org/10.1371/journal.pone.0267240

Stephens, T. M. (2012). Increasing resilience in adolescent nursing students. Dissertation, University of Tennessee.

Stockton, H., Joseph, S., & Hunt, N. (2014). Expressive writing and posttraumatic growth: An Internet-based study. *Traumatology: An International Journal, 20*(2), 75–83. https://doi.org/10.1037/h0099377

Sutarto, A. P., Wahab, M. N., & Zin, N. M. (2012). Resonant breathing biofeedback training for stress reduction among manufacturing operators. *International journal of occupational safety and ergonomics : JOSE*, *18*(4), 549–561. https://doi.org/10.1080/10803548.2012.11076959

Tagalidou, N., Baier, J., & Laireiter, A. R. (2019). The effects of three positive psychology interventions using online diaries: A randomized-placebo controlled trial. *Internet interventions*, *17*, 100242. https://doi.org/10.1016/j.invent.2019.100242

van Berkel, J., Boot, C. R. L., Proper, K. I., Bongers, P. M., & van der Beek, A. J. (2014). Effectiveness of a worksite mindfulness-related multi-component health promotion intervention on work engagement and mental health: Results of a randomized controlled trial. *PLoS ONE, 9*(1), Article e84118. https://doi.org/10.1371/journal.pone.0084118

van der Houwen, K., Schut, H., van den Bout, J., Stroebe, M., & Stroebe, W. (2010). The efficacy of a brief internet-based self-help intervention for the bereaved. *Behaviour research and therapy*, *48*(5), 359–367. https://doi.org/10.1016/j.brat.2009.12.009

van der Meer, C. A. I., Bakker, A., van Zuiden, M., Lok, A., & Olff, M. (2020). Help in hand after traumatic events: a randomized controlled trial in health care professionals on the efficacy, usability, and user satisfaction of a self-help app to reduce trauma-related symptoms. *European journal of psychotraumatology*, *11*(1), 1717155. https://doi.org/10.1080/20008198.2020.1717155

Villani, D., Grassi, A., Cognetta, C., Toniolo, D., Cipresso, P., & Riva, G. (2013). Self-help stress management training through mobile phones: an experience with oncology nurses. *Psychological services*, *10*(3), 315–322. https://doi.org/10.1037/a0026459

Wijesekera, K., Aralis, H., Sinclair, M., Cosanella, T., Alejos, J., & Lester, P. (2019). Innovative behavioral health screening and resilience building intervention for pediatric heart transplant youth and families: The focus-PEDSHT program. *Pediatric Transplantation*, 22.

Yousefi, A., Naeimijoo, P., Ghadiany, M., Lighvan, M. A., Bakhtiyari, M., & Arani, A. M. (2022). A randomized control trial of the effectiveness of online Mindfulness-Based Cancer recovery program on psychological well-being, caregiver burden and resilience in cancer patients’ caregivers. *Medical Science, 26*(123), 1. https://doi.org/10.54905/disssi/v26i123/ms181e2098

Zahedifar, F., Nejatifar, Z., Rafiei, S., & Hashemi, F. (2021). The effect of educational intervention on anxiety and quality of life among individuals referring to healthcare centers in the face of COVID-19. Acta Medica Iranica. https://doi.org/10.18502/acta.v59i8.7252
